# Depth-resolved transcriptional activity of antibiotic resistance genes in deep permafrost of the Qinghai–Tibet Plateau

**DOI:** 10.1038/s42003-026-10593-2

**Published:** 2026-06-30

**Authors:** Wasim Sajjad, Shichang Kang, Xiufeng Yin, Xiaodong Wu, Fei Deng, Christopher Williamson, Muhammad Rafiq

**Affiliations:** 1 State Key Laboratory of Cryospheric Science and Frozen Soil Engineering, Northwest Institute of Eco-Environment and Resources, Chinese Academy of Sciences, Lanzhou, China; 2 Institute of Mountain Hazards and Environment (IMHE), Chinese Academy of Sciences, Chengdu, China; 3 Key Laboratory of Virology and Biosafety and National Virus Resource Center, Wuhan Institute of Virology, Chinese Academy of Sciences, Wuhan, China; 4 Bristol Glaciology Centre, School of Geographical Sciences, University of Bristol, Bristol, UK; 5 Guizhou Key Laboratory of Microbiome and Infectious Disease Prevention and Control & Key Laboratory of Environmental Pollution Monitoring and Disease Control, Ministry of Education of Guizhou & School of basic Medical Sciences & Institution of One health research, Guizhou Medical University, Guiyang, China; 6 Department of Microbiology, Faculty of Life Sciences and Informatics, Balochistan University of Information Technology, Engineering and Management Sciences, Quetta, Pakistan

**Keywords:** Microbial ecology, Soil microbiology

## Abstract

The Qinghai–Tibet Plateau harbors permafrost ecosystems containing diverse antibiotic resistance genes (ARGs), yet their transcriptional activity and depth-resolved patterns remain poorly understood. Here, we performed meta-transcriptomic profiling of two permafrost cores (40-m and 100-m) from the Tuotuo River Basin. Within each core, permafrost layers exhibited higher relative ARG transcript abundance and diversity than overlying active layers. β-Lactam, multidrug, and puromycin resistance transcripts were enriched in permafrost, whereas aminoglycoside resistance predominated in the active layer. Antibiotic inactivation mechanisms were more prevalent in permafrost, while target modification dominated in the active layer within the analyzed cores. Plasmid-associated ARGs were detected, and mobile genetic elements (MGEs) showed stratified distributions, with integrative and conjugative elements more abundant in active layers and integrons enriched in permafrost strata. ARG transcript levels were associated with microbial community composition, MGEs, metal tolerance genes, and trace metal concentrations. Neutral community modeling suggested that ARG assemblages are largely consistent with stochastic processes, indicating long-term persistence under environmental filtering. As the analysis is limited to two cores, these observations reflect site-specific transcriptional patterns rather than generalizable regional trends. Overall, this study provides evidence of ARG transcripts in permafrost layers, offering a baseline for future investigations of resistome dynamics under thaw conditions.

## Abstract (web summary)

Meta-transcriptomic profiling of deep Qinghai–Tibet Plateau permafrost reveals depth-stratified antibiotic resistance transcripts, microbial associations, and ecological patterns suggesting persistent resistome signatures in frozen environments.

## Introduction

Antibiotic resistance genes (ARGs) are widespread across environmental and host-associated microbiomes and represent a recognized challenge to public health by reducing antimicrobial efficacy^1–5^. While ARG distribution and evolution have been widely studied in clinical, agricultural, and aquatic systems^6–8^, their transcriptional activity and persistence in extreme, low-energy ecosystems, particularly pristine permafrost, remain poorly understood.

Permafrost underlies approximately 20% of Earth’s terrestrial surface and functions as a cryogenic repository that can preserve microbial communities and genetic material over long timescales^9–15^. The Qinghai–Tibet Plateau (QTP), colloquially referred to as “The Third Pole,” hosts vast and climate-sensitive permafrost zones that harbor exceptional microbial diversity and evidence of long-term evolutionary adaptation^16–18^. Detection of ARGs in these frozen sediments suggests that resistance traits can exist independently of recent anthropogenic influence, supporting hypotheses of ancient origins^2,19^. Natural antibiotics are thought to have emerged between 2 billion and 40 million years ago^20,21^, indicating that corresponding resistance mechanisms may have co-evolved with microbial lineages over geological timescales^19^.

Although microbial biomass and diversity typically decline with soil depth^22–24^, permafrost environments preserve ancient microbial assemblages^14,17^, providing a window into evolutionary dynamics in cold, low-energy environments. The QTP permafrost, representing the largest continuous high-altitude frozen biome, plays a vital role in regional hydrology, carbon flux, and microbial ecology^16,25–28^. Climate-driven deepening of the active layer^29,30^ raises questions about whether permafrost-hosted ARGs could be mobilized upon thaw^14,31,32^. However, empirical data on active ARG transcription in permafrost are limited, and the biological significance of such activity remains to be clarified.

Permafrost has historically been considered biologically inert due to subzero temperatures and nutrient limitation. However, growing evidence demonstrates that microbial activity, including genome replication, can occur at subzero temperatures in permafrost environments^33^. In this context, deeper permafrost may harbor distinct resistome signatures shaped by long-term isolation and environmental filtering. Meta-transcriptomics provides a powerful approach to assess in situ functional resistance by capturing real-time gene expression, surpassing the static view offered by DNA-based metagenomics^34,35^. In cryogenic ecosystems, where thaw can reactivate dormant life^14,31^, this approach enables the detection of expressed ARGs, associated mobile elements, and potential regulatory or horizontal gene transfer (HGT) activity responsive to environmental stimuli^36,37^.

To our knowledge, no previous study has employed a meta-transcriptomic framework to characterize ARG transcripts in deep permafrost. Here, we present a depth-resolved meta-transcriptomic analysis of ARGs in two permafrost cores (TTH-04, 40 m; TTH-07, 100 m) from the Tuotuo River basin, the headwaters of the Yangtze River and an ecologically and hydrologically significant region. Using high-throughput sequencing, integrated environmental metadata, and bioinformatics analyses, we examine ARG diversity, expression patterns, and co-occurrence with mobile genetic elements (MGEs) and metal tolerance genes (MTGs), and explore potential ecological drivers. By focusing on transcriptionally active genes, rather than genomic reservoirs, this study provides site-specific insights into the functional ecology and long-term persistence of resistance determinants in permafrost and sub-permafrost layers. Given the limited number of cores, the findings are interpreted as site-specific patterns, providing a foundation for future investigations of resistome dynamics under permafrost thaw in the Anthropocene.

## Results

### Characterization of sequencing data

Approximately 270 GB of sequence data (~1.8 billion paired-end reads) were generated across all samples, yielding an average of ~72 million raw and ~66 million clean reads per sample after quality filtering (Supplementary Table 1). Relative transcript abundance peaked at ~20 m depth in both TTH-04 (sub-permafrost) and TTH-07 (permafrost), indicating elevated microbial activity in geochemically stable zones. Distinct stratified expression patterns were observed for ARGs, MGEs, MTGs, and core metabolic pathways.

### Stratification and diversity of ARGs

**Fig. 1:**
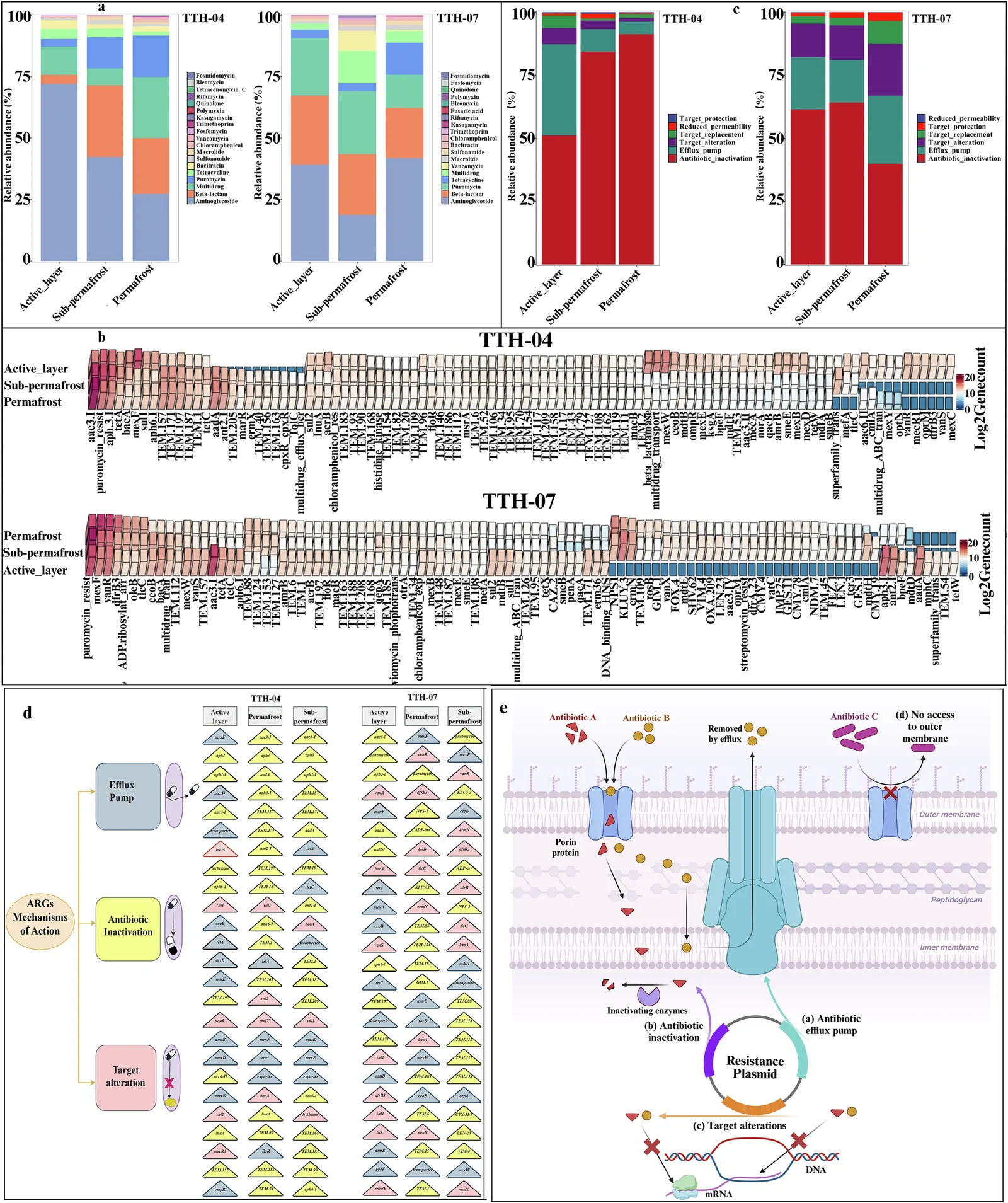
Depth-resolved expression profiles and functional characterization of antibiotic resistance genes (ARGs) in permafrost cores TTH-04 and TTH-07. **a** Relative transcript abundance (%) of 19 ARG classes detected across depth-stratified samples from cores TTH-04 (40 m; *n* = 10 subsamples) and TTH-07 (100 m; *n* = 15 subsamples). Subsamples represent depth-defined horizons (active layer, permafrost, and sub-permafrost) within each core. **b** Three-dimensional heatmap showing expression patterns (TPM-normalized) of the top 100 most abundantly expressed ARGs across sampled depths in both cores. **c** Distribution of ARG transcripts grouped by resistance mechanism, based on functional annotation of meta-transcriptomic assemblies. **d** Relative contribution of the top 25 expressed ARGs and their associated resistance mechanisms across permafrost layers. **e** Conceptual schematic summarizing the principal antibiotic resistance mechanisms represented by the expressed ARGs detected in this study, including antibiotic inactivation, efflux, target modification, and target protection. The schematic is provided to facilitate interpretation of the resistance mechanisms represented by the expressed ARGs identified in this study. Illustration created with BioRender.com.

Across both cores, 19 ARG classes comprising 380 subtypes displayed depth-associated stratification in relative expression patterns. In TTH-04, aminoglycoside resistance dominated the active layer (72%), whereas multidrug (25%), β-lactam (23%), and puromycin (17%) transcripts were enriched in permafrost strata. Sub-permafrost layers sustained aminoglycoside dominance (42%) with elevated β-lactam (29%) and puromycin (13%) expression. TTH-07 showed similar trends, with aminoglycoside genes enriched in both active (39%) and permafrost (42%) layers, while puromycin resistance peaked in sub-permafrost strata (26%) (Fig. 1a).

Expression profiles varied across depth intervals in both cores. In TTH-04, aminoglycoside resistance peaked at 1–2 m and again at 15 m, coinciding with changes in redox and nutrient conditions, suggesting depth-specific differences in relative transcript representation, potentially reflecting microbial activity. β-lactam, puromycin, and multidrug ARGs were concentrated in discrete zones, consistent with associations with local microbial communities or geochemical microenvironments (Supplementary Fig. 1a). TTH-07 displayed more uniform aminoglycoside expression, with reductions at the shallowest and deepest depths, where puromycin and β-lactam expression increased, potentially reflecting specialized stress-adapted microbial assemblages. Tetracycline resistance peaked at intermediate depths (5–10 m and ~50 m), consistent with zones of elevated microbial turnover or metal-associated co-selection.

ARG diversity also varied by depth. TTH-04 displayed relatively stable diversity in the active layer but greater variability in deeper horizons, consistent with strong stratification of microbial activity. TTH-07 showed more heterogeneous diversity across depths, suggesting a more complex or heterogeneous community structure (Supplementary Fig. 1b).

**Fig. 2:**
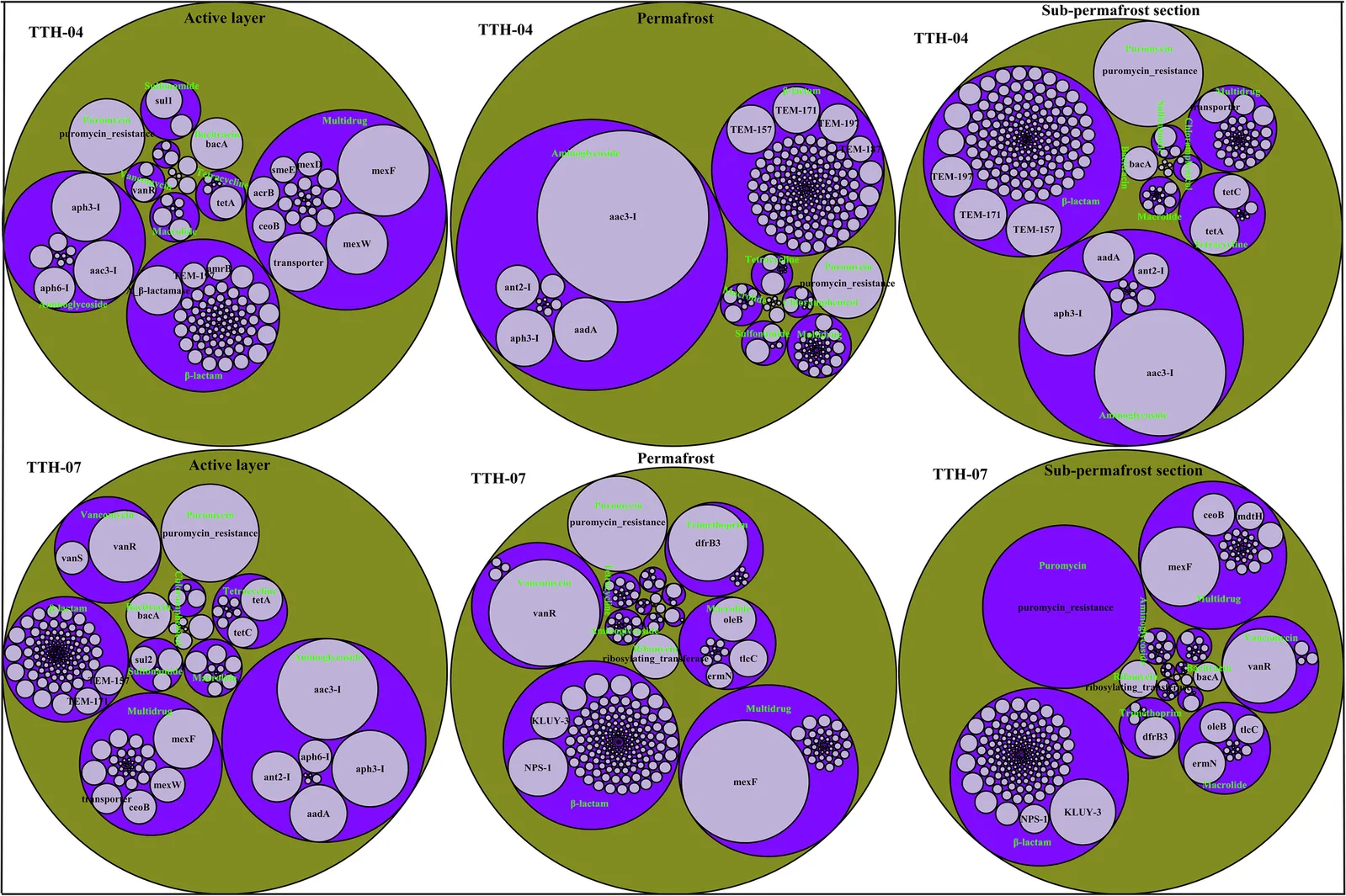
Depth-resolved composition and distribution of expressed antibiotic resistance genes (ARGs) in permafrost cores TTH-04 and TTH-07. Circular plots summarize the composition and relative transcript abundance of ARGs across depth-defined horizons in the two permafrost cores. The diagram is read from the outer ring inward. The outer ring (dark yellow) indicates stratigraphic layers (active layer, permafrost, and sub-permafrost). The second ring (violet) represents ARG classes detected within each layer. Within each class, individual ARGs are shown as light-indigo circles, with circle size proportional to normalized transcript abundance (TPM), enabling comparison of relative expression among genes and depths. ARG labels are displayed only for genes contributing >1% of the total ARG transcript abundance within a given layer to improve readability. The category “macrolide” includes macrolide–lincosamide–streptogramin (MLS) resistance genes. This visualization integrates ARG class structure, gene-level contributions, and depth-specific expression patterns to facilitate comparison across stratigraphic horizons and between cores.

ARG subtypes displayed clear layer-specific distributions. At TTH-04, *mexF*, *aph3-I*, and *puromycin resistance* were prominent in the active layer, while *aac3-I*, *puromycin resistance* and *TEM-157* were enriched beneath the active layer. At TTH-07, the active layer harbored *aac3-I*; *puromycin resistance; vanR; and mexF*, while the permafrost layer was dominated by *mexF; vanR; puromycin resistance; dfrB3*; *NPS-1*; *oleB;* and *ADP-ribosylating transferase*. Sub-permafrost layers featured enrichment of *puromycin resistance*, *mexF*, *KLUY-3*, *vanR*, *ermN*, and *dfrB3* (Fig. 2).

The top 100 ARGs exhibited layer-specific absence patterns. In TTH-04, 11, 9, and 11 ARGs were undetected in the active, permafrost, and sub-permafrost layers, respectively; TTH-07 displayed more exclusions, with 31 ARGs absent or reduced in the active layer (Fig. 1b). Resistance mechanisms also varied with depth (Fig. 1c). In TTH-04, antibiotic inactivation predominated in permafrost (91.2%) and sub-permafrost (84.3%), while the active layer displayed a more balanced mechanistic profile (inactivation: 51.1%; efflux: 36.2%; target alteration: 11.8%). TTH-07 exhibited broader mechanistic diversity in permafrost (inactivation: 40%; efflux: 27%; target alteration: 20.4%; replacement: 9.2%). The top 25 ARGs and associated mechanisms are shown in Fig. 1d, e. Overall, ARG composition and resistance mechanisms differed systematically across soil horizons, with the active layer exhibiting greater heterogeneity and deeper layers showing more constrained but distinct profiles.

### Genomic context of ARGs and distribution of MGEs/MTGs

**Fig. 3:**
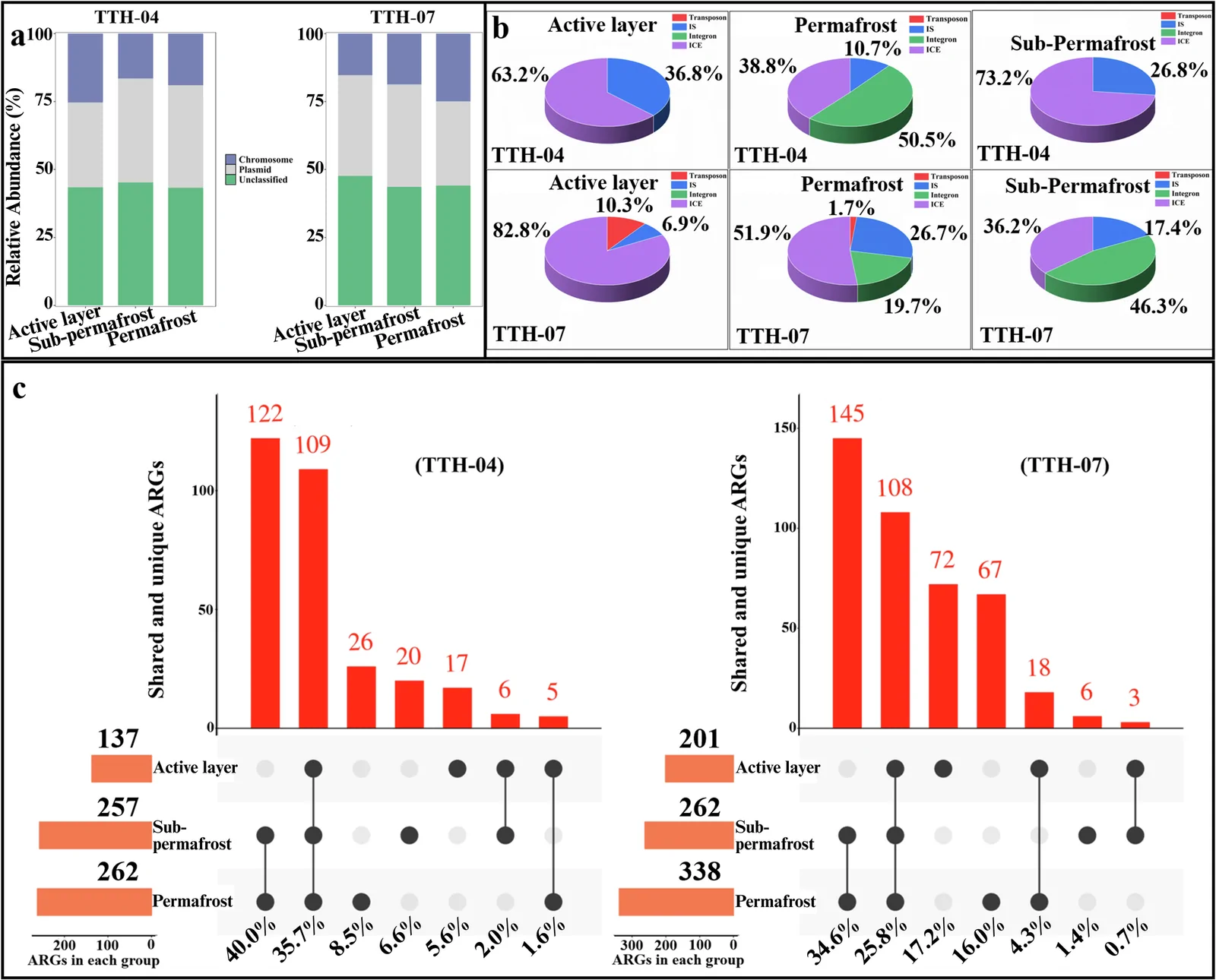
Depth-resolved distribution of ARG genomic context, mobile genetic elements, and ARG uniqueness across permafrost layers. **a** Relative abundance (%) of ARG transcripts assigned to putative plasmid, chromosomal, or unclassified genetic contexts across the active layer, permafrost, and sub-permafrost. Genomic context was inferred from contig-level annotations and reflects putative rather than resolved localization. **b** Relative abundance of mobile genetic element (MGE)-associated transcripts, including integrative and conjugative elements (ICEs), integrons, insertion sequences (IS), and transposons, across permafrost layers. **c** UpSet plots showing the proportion of unique and shared ARG subtypes across depth-defined layers (active layer, permafrost, and sub-permafrost) within each core. Vertical bars indicate the proportion of ARG subtypes associated with each intersection of depth layers. Single-layer intersections represent ARGs unique to a given layer, whereas multi-layer intersections indicate ARGs shared among two or more layers. The proportion of ARG subtypes was significantly higher in permafrost and sub-permafrost layers than in the active layer (Kruskal–Wallis test with Dunn’s post hoc correction, *p* < 0.05), except for the comparison between permafrost and sub-permafrost at TTH-04 (*p* > 0.05). Detailed ARG subtype lists and statistical outputs are provided in Supplementary Data 3.

ARGs were differentially assigned to chromosomes, plasmids, and unclassified contigs. In TTH-04, a larger proportion of ARG transcripts mapped to plasmid-assigned contigs in sub-permafrost layers (38.3%), whereas chromosomal assignments dominated the active layer (25.3%). TTH-07 showed increased chromosomal ARG prevalence in permafrost (25.0%) (Fig. 3a).

MGEs, including integrative and conjugative elements (ICEs), were prevalent in active layers (TTH-04: 63.2%; TTH-07: 82.8%), while integrons were enriched in permafrost (TTH-04: 50.5%; TTH-07: 19.7%) and sub-permafrost (TTH-07: 46.3%). Transposons were largely restricted to active and permafrost layers in TTH-07 (Fig. 3b). These annotations indicate potential activity, but do not confirm active excision, integration, or horizontal transfer. Details characteristics of MGEs are provided in (Supplementary Data 1 and 2).

MTGs were enriched below the active layer, notably mercury (*merR*, *merT*) and copper (*copZ*, *copC*) resistance genes, while *cmtR* and *arsC* displayed site- and depth-specific patterns (Supplementary Fig. 2). Copper concentrations varied across layers (Supplementary Data 3; also reported in ref. ^38^), whereas mercury was not measured. Consequently, interpretations linking MTG enrichment to metal exposure remain speculative and may also reflect intrinsic microbial adaptation or historical selection processes.

### ARG spatial dynamics and correlates

**Fig. 4:**
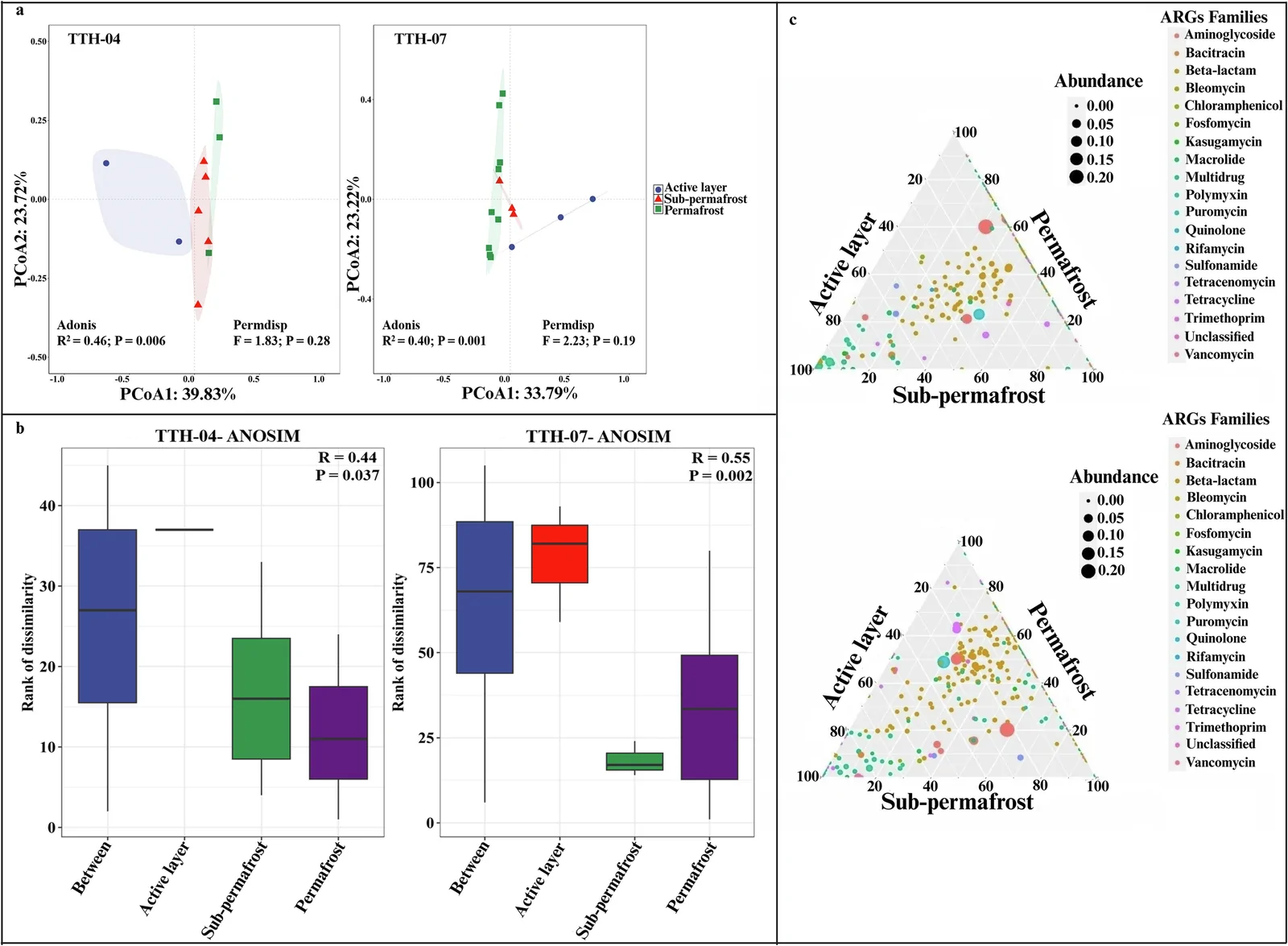
Depth-related structuring of expressed ARG communities across permafrost layers. **a** Principal coordinate analysis (PCoA) of ARG expression profiles based on Bray–Curtis dissimilarities, showing depth-resolved patterns across the active layer, permafrost, and sub-permafrost. Each point represents an individual stratified sample. Group differences were evaluated using permutational multivariate analysis of variance (PERMANOVA; Adonis), and dispersion homogeneity was assessed using permutation-based multivariate dispersion (Permidisp). **b** Analysis of similarity (ANOSIM) results comparing ARG community composition among permafrost layers, presented as box plots of R statistics. **c** Ternary plots illustrating the relative distribution of ARG transcripts across the three depth-defined layers. Each point represents an individual ARG, grouped by antibiotic resistance class, highlighting depth-associated expression preferences rather than exclusive localization.

ARG diversity peaked in permafrost and sub-permafrost strata. Diversity indices differed significantly among strata at TTH-04 (*P* < 0.05) but not at TTH-07 (Supplementary Fig. 3). ARG were significantly more diverse in permafrost and sub-permafrost layers than in the active layer (*P* < 0.05), though no significant distinction was found between permafrost and sub-permafrost at TTH-04 (*P* = 0.63) (Supplementary Data 3). Shared ARG subtypes between permafrost and sub-permafrost layers accounted for 40.0% (122/305) in TTH-04 and 34.6% (145/419) in TTH-07 within each core, while unique ARGs were enriched below the active layer (Fig. 3c). Beta-diversity analyses (PCoA based on Bray–Curtis dissimilarity) revealed depth-stratified ARG assemblages in both cores. This pattern was supported by PERMANOVA (Adonis; TTH-04: *P* = 0.006; TTH-07: *P* = 0.001) and ANOSIM (TTH-04: *R* = 0.44, *P* = 0.037; TTH-07: *R* = 0.55, *P* = 0.002), while PERMDISP analysis (TTH-04: *P* = 0.28; TTH-07: *P* = 0.19) indicated that these differences were not driven by dispersion heterogeneity (Fig. 4a, b). ARG composition and abundance exhibited clear stratification across layers, indicating depth-associated differences in resistome structure based on relative transcript composition (Fig. 4c; Supplementary Fig. 4).

**Fig. 5:**
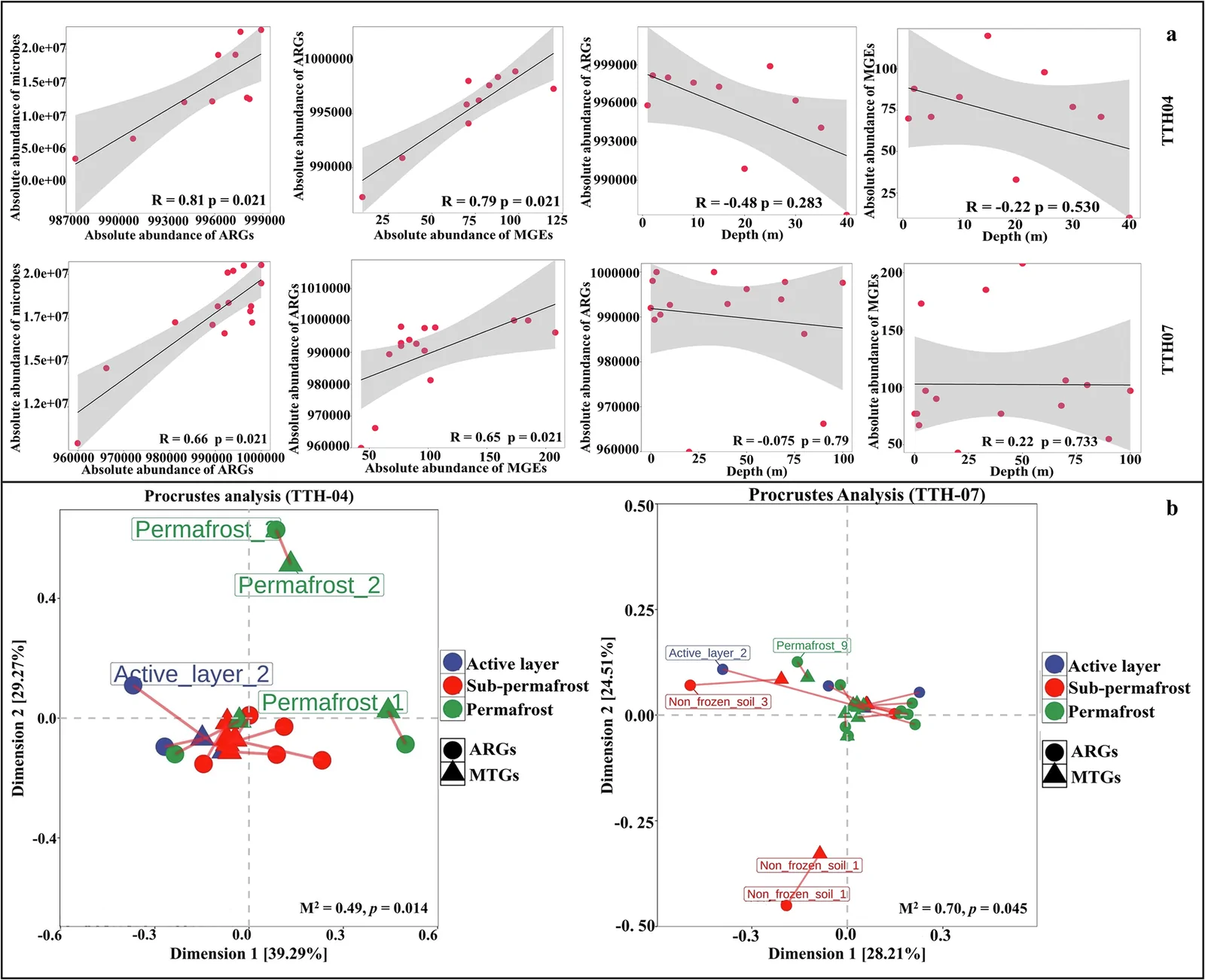
Depth-resolved associations among antibiotic resistance genes (ARGs), mobile genetic elements (MGEs), metal tolerance genes (MTGs), and microbial communities. **a** Spearman correlation analysis showing relationships between the relative transcript abundance of expressed ARGs, MGEs, dominant microbial taxa, and depth across stratified permafrost samples. Correlations reflect co-variation patterns rather than causal interactions. *P*-values (corrected) were adjusted for multiple testing using the Benjamini–Hochberg false discovery rate (FDR) correction, and significance is reported based on FDR-adjusted values. Exact correlation coefficients and adjusted *P*-values are provided in Supplementary Table 2 and Supplementary Data 4 for Fig. 5a. Reported *P*-values are rounded to three decimal places. **b** Procrustes analysis based on Bray–Curtis dissimilarities illustrating the concordance between ARG and MTG expression profiles across samples. Points represent individual samples, with shapes distinguishing ARGs (circles) and MTGs (triangles), and colors indicating permafrost layers (active layer, permafrost, and sub-permafrost). Shorter connecting vectors indicate stronger similarity between ARG and MTG community structures.

ARG expression was positively correlated with microbial abundance (TTH-04: *R* = 0.81, *P* = 0.021; TTH-07: *R* = 0.66, *P* = 0.021) and MGEs (TTH-04: *R* = 0.79, *P* = 0.021; TTH-07: *R* = 0.65, *P* = 0.021), whereas depth alone showed no significant relationship with ARG transcripts (TTH-04: *R* = −0.48, *P* = 0.283; TTH-07: *R* = −0.075, *P* = 0.79) and MGEs (TTH-04: *R* = −0.22, *P* = 0.530; TTH-07: *R* = 0.22, *P* = 0.733) (Fig. 5a; Supplementary Fig. 5a). Procrustes analysis indicated stronger ARG-MTG associations at TTH-04 (*M*² = 0.49, *P* = 0.014) than TTH-07 (*M*² = 0.70, *P* = 0.045) (Fig. 5b). RDA highlighted metal-specific ARG associations, e.g., Zn/Ni/Zr (TTH-04 sub-permafrost) and As/Si/Rb (TTH-04 permafrost) (Supplementary Fig. 5b). ARG families were positively associated with dominant microbial phyla (Supplementary Fig. 6), suggesting taxonomic patterns in expressed transcripts. These results indicate that variation in relative ARG expression is likely associated with the active microbial community and environmental gradients, while no significant relationship with depth alone was observed.

### Ecological assembly and network dynamics

**Fig. 6:**
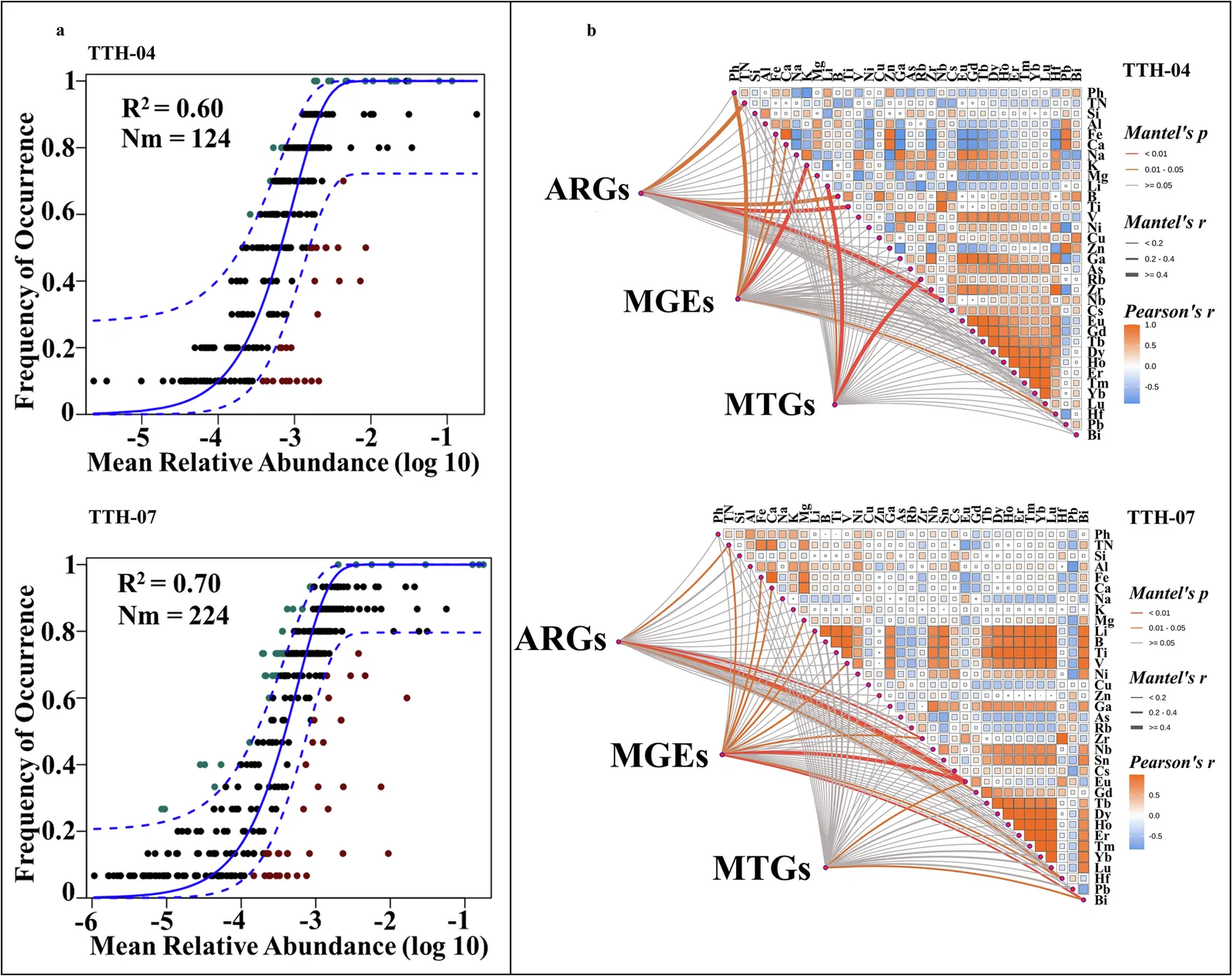
Community assembly patterns of expressed ARGs and their associations with environmental factors across permafrost layers. **a** Fit of the neutral community model (NCM) for ARG transcript communities across depth-resolved permafrost samples. The model evaluates the extent to which ARG occurrence frequencies conform to neutral expectations. *R* indicates the goodness-of-fit of the model, and *Nm* represents the estimated migration parameter. The solid blue line denotes the predicted neutral relationship, and dashed blue lines indicate the 95% confidence intervals. ARGs whose occurrence frequencies fall within the confidence interval (black points) are consistent with predominantly stochastic assembly processes, whereas ARGs deviating below the neutral prediction (red points) may reflect the influence of non-neutral factors such as environmental filtering or selective constraints. **b** Mantel and partial Mantel tests examining associations between community dissimilarities of ARGs, MGEs, and MTGs and measured environmental variables. Bray–Curtis distance matrices were used for biological communities, and each environmental factor was tested independently while controlling for other variables. Line width corresponds to the Mantel *r* statistic, and line color indicates statistical significance based on 999 permutations. Statistical significance shown in the figure is based on unadjusted *p*-values for exploratory visualization, whereas FDR-adjusted significance (Benjamini–Hochberg correction) is reported in the Supplementary Data 4 for Fig. 6b and used for inference in the main text. No adjustment for multiple testing was applied in the figure to preserve the interpretability of correlation patterns across environmental gradients.

Neutral community models (NCM) attributed 60–70% of ARG variation to stochastic processes (Fig. 6a). TTH-07 exhibited higher immigration rates (Nm = 224) than TTH-04 (Nm = 124), suggesting greater ARG exchange and community dynamism. Nonetheless, deterministic forces, particularly trace metal concentrations, were critical in ARG selection. Wilcoxon tests showed no significant differences in ARG class abundance between sites, suggesting functional convergence (Supplementary Fig. 7b).

Trace element associations were observed under nominal significance thresholds (Mantel *r* ≥ 0.3, *P* ≤ 0.05; Fig. 6b); however, after false discovery rate correction (FDR; *q* < 0.05), only a limited subset remained significant. At TTH-04, ARGs were associated with Ti and Nb, MGEs with K, and MTGs with Rb and Li. At TTH-07, only ARG and MGE associations involving Eu remained significant (Supplementary Table 2 and Supplementary Data 4 for Fig. 6b). Because correlations were pre-filtered before correction, retained associations reflect relatively strong effect sizes. Other elements showed moderate but non-significant effects, indicating weaker or context-dependent influences.

**Fig. 7:**
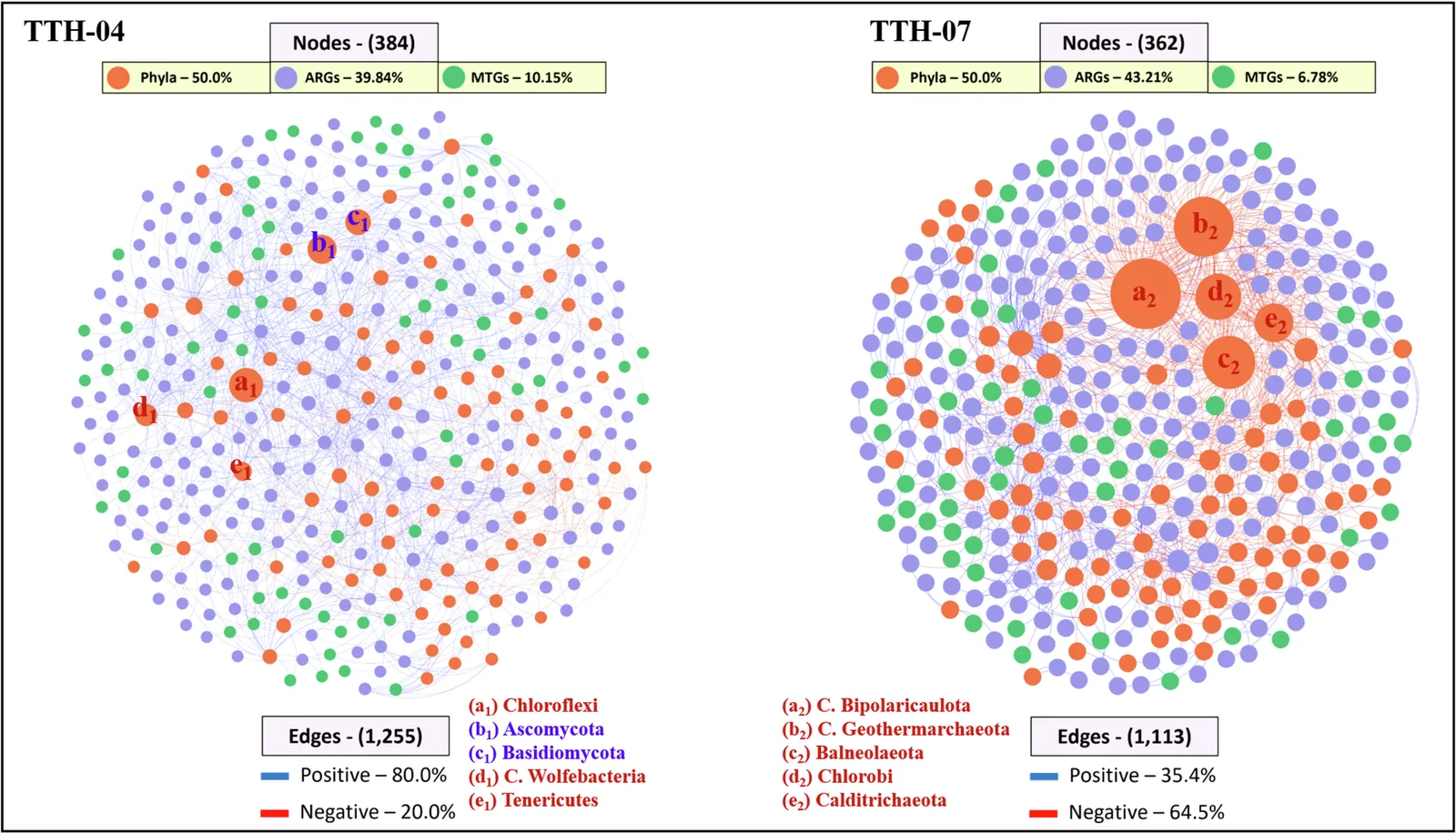
Co-occurrence network of expressed ARGs, MTGs, and microbial taxa across permafrost cores. Network visualization illustrating correlation-based co-occurrence patterns among antibiotic resistance genes (ARGs), metal tolerance genes (MTGs), and microbial taxa inferred from meta-transcriptomic profiles. Nodes are color-coded by category as indicated in the legend. Edges represent statistically significant (*P* < 0.05) and strong (| r | >  0.65) Pearson correlations, reflecting co-variation rather than direct interaction or gene transfer. Edge thickness is proportional to correlation strength. Node size corresponds to degree centrality (number of connections) and does not indicate relative abundance or expression level. The five taxa with the highest degree centrality in each core are labeled (bacterial phyla in red; fungal phyla in dark blue) using core-specific identifiers (a1–e1 for TTH-04; a2–e2 for TTH-07) to avoid cross-core comparisons. Blue edges indicate positive correlations, whereas red edges indicate negative correlations. Statistical significance in the network is based on unadjusted p-values; false discovery rate (FDR)-corrected significance across all tested associations is reported in the Supplementary Data 4 for Fig. 7. In TTH-04, 1121 out of 1255 associations remained significant after correction (*q* < 0.05), while in TTH-07 all tested associations remained significant, indicating that the inferred co-occurrence network structure is robust across both cores.

Network analyses revealed differing architectures: TTH-04 was dominated by positive ARG–MTG associations (80%), whereas TTH-07 had more negative associations (64.5%) (Fig. 7). Bacteria and fungi primarily structured networks at TTH-04, while bacteria and archaea dominated at TTH-07. Networks were modular but sparsely connected (Supplementary Table 3).

**Fig. 8:**
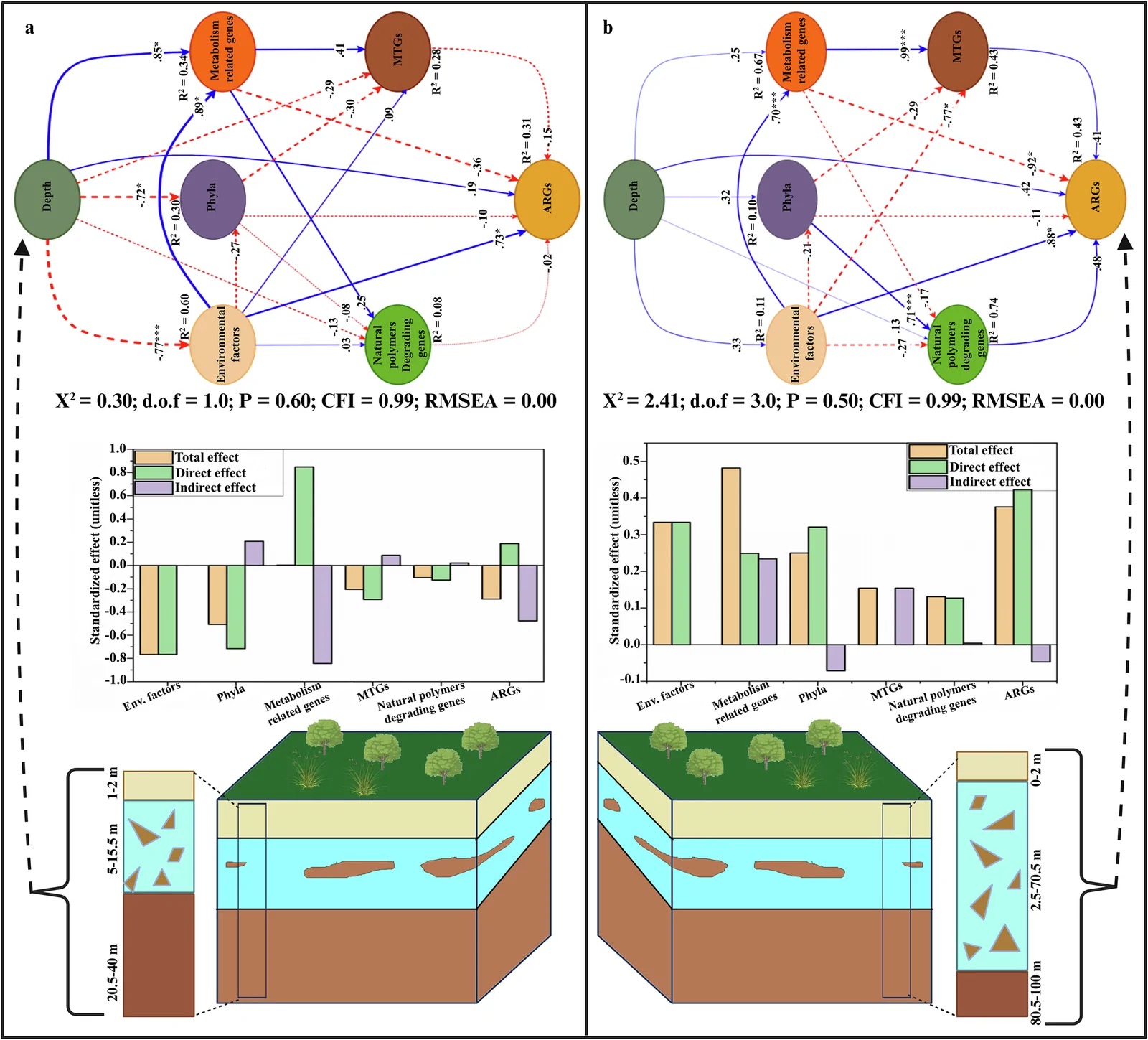
Structural equation models (SEMs) describing factors associated with ARG transcript abundance in permafrost cores TTH-04 and TTH-07. SEMs illustrate hypothesized relationships among depth, environmental factors, carbon cycle–related genes, microbial taxa, mobile genetic elements (MGEs), polymer-degrading genes, and ARG transcript abundance in TTH-04 (**a**) and TTH-07 (**b**). Single-headed arrows represent model-specified directional relationships based on ecological assumptions rather than demonstrated causation, with arrow width proportional to standardized path coefficients. Green solid arrows indicate positive associations, whereas red dashed arrows indicate negative associations. Circles represent composite variables summarized as the first principal component (PC1) derived from principal component analysis (PCA). Bar plots show the standardized total effects of individual predictors on ARG transcript abundance within each core. Statistical significance is indicated by asterisks (***P < 0.001; **P < 0.01; *P < 0.05).

SEM explained 31% (TTH-04) and 43% (TTH-07) of ARG variance (Fig. 8). At TTH-04, depth promoted metabolic genes and suppressed environmental variability, microbial phyla and MTGs, while environmental factors strongly enhanced ARGs and metabolism. At TTH-07, depth had stronger direct effects on ARGs composition and microbial phyla, while environmental factors boosted ARGs expression and metabolism but suppressed MTGs. Microbial phyla positively modulating polymer-degrading genes, which in turn promoted ARG expression, suggesting that enhanced substrate availability and microbial activity can promote ARG expression and potential HGT. However, some phyla exerted direct negative effects on ARG abundance, highlighting the complex interplay between metabolic activity and resistance traits. Metabolism promoted MTGs but suppressed ARGs, with both polymer-degrading genes and MTGs supporting ARG expression.

## Discussion

The extraordinary adaptability of microbes, driven by mechanisms such as HGT, metabolic versatility, and antimicrobial resistance evolution, underscores a long-standing evolutionary plasticity^39,40^. Here, we investigate antibiotic resistance in deep permafrost horizons from the TuoTuo River Basin, offering RNA-based evidence of ARG transcriptional signatures preserved within deep permafrost horizons that likely predate direct modern anthropogenic influence. However, the absence of direct geochronological constraints and the potential for stratigraphic reworking preclude definitive temporal attribution. Our meta-transcriptomic data support the presence of potentially active microbial functions in deep frozen sediments, complementing previous studies showing that permafrost harbors a complex assemblage of viable, dormant, and viable-but-non-culturable cells, alongside partially degraded biomass^41–44^. Metagenomic evidence further indicates that long-term freezing selects for survival strategies such as dormancy, cellular repair, and stress responses, rather than sustained active proliferation^45^. Although metabolic activity under in situ conditions is strongly constrained by extreme environmental stress, these communities retain the capacity for functional responsiveness under favorable conditions^11^.

Despite enduring subzero temperatures, oligotrophic conditions, and prolonged cryogenic isolation, ARG transcripts were detectable throughout permafrost layers. These findings support the Baas-Becking hypothesis^46,47^—“everything is everywhere, but the environment selects”—illustrating that certain resistome components can persist and remain transcriptionally detectable under long-term cryogenic conditions^48^.

Elevated relative expression of ARG transcripts beneath the active layer may reflect low-level or episodic metabolic activity sustained by geochemical stability and limited labile carbon. In contrast, the active layer, subject to seasonal thaw, redox fluctuations, and hydrological disturbance, displayed comparatively lower ARG diversity and transcription, likely reflecting dominance by opportunistic taxa adapted to dynamic conditions^11,18,24,49–54^. Consistent with these ecological contrasts, deeper permafrost layers displayed more constrained yet mechanistically differentiated ARG profiles shaped by long-term environmental filtering, low energy flux, and cryogenic preservation^11^. These results extend DNA-based observations^55,56^ by demonstrating transcriptional signals in deep permafrost. Transcript abundances reported here are based on transcripts per million (TPM) normalization and therefore represent relative expression within the sequenced transcript pool rather than absolute quantities. Accordingly, depth-related patterns should be interpreted primarily as shifts in the relative representation of ARG transcripts, rather than direct measures of total transcriptional activity or absolute gene expression levels.

A shared resistome across the cores indicates genetic continuity that may reflect historical HGT, vertical inheritance, or long-term ecological persistence, even in frozen and spatially constrained environments. This suggests that MGEs may retain functional relevance in deep cryoenvironments, although the extent of active HGT under such conditions remains uncertain. Conserved ARGs dominated the active layer, whereas ARGs unique to deeper layers were enriched beneath it (Fig. 3c), suggesting that deep layers in these cores may represent reservoirs of genetic novelty^57,58^. This stratification aligns with depth-related gradients in temperature, carbon availability, redox potential, and permafrost age^59,60^, collectively shaping the resistome architecture. Although associations among ARGs, MGEs, and microbial taxa are consistent with HGT potential, the ecological feasibility of active gene transfer in permafrost is constrained by limited microbial mobility, reduced cell–cell contact, and frozen microstructures^61^, which restrict opportunities for canonical HGT mechanisms. Although HGT has been documented in deep subsurface microbial systems under low-energy conditions^62^, these environments may not be directly comparable to permanently frozen permafrost. In this context, the observed ARG patterns likely reflect a combination of historical HGT, vertical inheritance, and long-term ecological filtering rather than ongoing gene transfer.

Interpretation of depth-resolved transcriptomic signals must consider several sources of uncertainty. The absence of direct geochronological constraints limits inference of absolute ages, and although large-scale cryoturbation was not observed during field observations, finer-scale or historical mixing processes cannot be excluded. Past glacial and periglacial dynamics may also have influenced sediment deposition and secondary burial of organic material in the region. Finally, while continuous permafrost generally restricts vertical hydrological transport, subsurface connectivity cannot be completely ruled out in heterogeneous systems. Together, these factors suggest that observed patterns reflect long-term ecological filtering rather than perfectly discrete or undisturbed stratigraphic signals, and the data do not allow direct inference of ancient transfer events or in situ HGT timing. Moreover, depth represents a continuous environmental gradient, and grouping samples into discrete horizons simplifies this complexity. However, these horizons are defined based on established cryostratigraphic and thermal boundaries that represent ecologically distinct regimes (active layer, permafrost, and sub-permafrost). The absence of a significant relationship between depth (as a continuous variable) and ARG expression further indicates that observed patterns likely reflect transitions between ecologically distinct strata rather than linear depth effects. Nevertheless, the use of pseudo-replicates within individual cores and categorical grouping may limit statistical independence, and results should therefore be interpreted as within-core, depth-resolved patterns rather than fully independent comparisons.

Resistance class distribution varied with depth. Aminoglycoside transcripts predominated in the active layer, consistent with higher microbial turnover and potential MGEs association^63–66^. Deeper horizons were enriched in β-lactam, puromycin, tetracycline, and multidrug ARGs, potentially reflecting long-term evolutionary filtering rather than recent acquisition^67^. However, detected transcript patterns in deeper permafrost may not exclusively represent active in situ expression. Persistence of nucleic acids from microbial necromass, as well as thaw-induced transcriptional responses or exposure to labile organic substrates, may contribute to observed signals^68,69^. Therefore, depth-associated patterns likely reflect a combination of active expression, preserved nucleic acids, and thaw-responsive transcription rather than exclusively stable evolutionary selection. These results suggest that intrinsic resistomes can persist under energy-limited conditions, extending beyond the anthropocentric perspective on antibiotic resistance^70^.

Consistent with these stratified patterns, antibiotic inactivation emerged as the predominant resistance strategy beneath the active layer in these cores, consistent with reports of diverse β-lactamases in remote Alaskan soils^71^, suggesting potentially similar functional patterns across cold environments. This strategy, based on enzymatic degradation or modification of antimicrobial compounds, may confer a selective advantage in oligotrophic environments, where energy conservation and competitive survival are paramount^9^. Efflux pumps and ribosomal protection genes further suggest ancestral defense strategies against endogenous antimicrobials^70,72^. In the active layer, mechanistic diversity was broader, likely reflecting seasonal metabolic flux and microbial turnover^52^. The presence of diverse resistance mechanisms in deeper layers suggests that resistome architecture is shaped not only by contemporary ecological interactions but also by long-term preservation and legacy signals. However, distinguishing between active in situ processes and preserved functional signatures remains challenging based on the current dataset.

ARG-associated reads were assigned to plasmid, chromosomal, and unclassified contexts; however, these assignments remain putative due to the absence of genome-resolved assemblies. The chromosomal ARGs, often considered part of the “core resistome”, beneath the active layers are more likely to be conserved, rather than recently acquired, and less reliant on HGT. Plasmid-associated ARGs may represent mobile potential, but actual HGT events cannot be inferred from these data. Numerous unclassified ARGs lacking homologs in reference databases were inferred from conserved resistance-associated domains without significant matches in SARG 2.0 (Supplementary Methods 1.3), highlighting an untapped genetic reservoir preserved in deep permafrost.

Strong correlations among ARGs, MGEs, and microbial taxa indicate potential functional associations rather than direct evidence of gene transfer. These patterns indicate that ARG distribution is structured by associations with specific microbial taxa and MGEs. ICEs and integrons were frequently co-located with ARGs, supporting their involvement in HGT and adaptive plasticity^67,70^. ICEs were abundant in active layers, likely promoting gene mobilization under seasonal stress^73^, while integrons, which dominated permafrost strata, may provide long-term stability and facilitate expression of ARG cassettes. This stratified pattern underscores the divergence in selective pressures across depth profiles. Consistent with this, the observed ARG–MGE associations suggest potential for gene mobilization within permafrost microbial communities; however, the data do not provide evidence for active excision, integration, or horizontal gene transfer. Insertion sequence (IS) elements beneath the active layer may indicate increased genome plasticity under prolonged cryogenic stress and low microbial turnover. While such patterns are consistent with ecological frameworks such as the Stress Gradient Hypothesis^74^, which propose that environmental stress can alter microbial interactions and network structure, the present data do not directly test these mechanisms. Localized enrichment of transposons at site TTH-07 may reflect microenvironmental heterogeneity, such as mineral interfaces or freeze-thaw cycles. These findings potentially highlight genetic plasticity even in geophysically isolated settings, with implications for ARG mobilization under future thaw scenarios^75,76^.

Taxonomic associations of ARG transcripts varied between sites and reflect transcriptionally active microbial taxa, providing ecological context for interpreting underlying expressed resistomes across depth horizons. At TTH-04, Bacillota (formerly Firmicutes) and Methanobacteriota (formerly Euryarchaeota) contributed disproportionately to the expressed resistome, consistent with vertical inheritance and physiological resilience, with endospore-forming Bacillota potentially serving as persistent ARG reservoirs^77^. Inverse association between fosmidomycin resistance and Pseudomonadota (formerly Proteobacteria) may reflect lineage-specific differences in isoprenoid biosynthesis pathways^78^, where alternative metabolic strategies reduce selective pressure for this resistance trait.

At TTH-07, polymyxin resistance was associated with Acidobacteria, Cyanobacteria, and Thaumarchaeota, suggesting that membrane remodeling or efflux-based defenses contribute to resistance expression across phylogenetically diverse taxa. The strong linkage of *Deinococcus–Thermus* with bacitracin, quinolone, and multidrug resistance aligns with its exceptional stress tolerance and DNA repair capacity^79^, which may favor retention of broad-spectrum resistance traits. Fosmidomycin resistance in Basidiomycota and Cyanobacteria may reflect ancient interdomain gene transfer or convergent adaptation to nutrient limitation, particularly phosphorus scarcity^78^, while tetracycline resistance in Actinobacteria and Cyanobacteria supports the antiquity of intrinsic resistomes. In contrast, inverse associations between β-lactam or puromycin resistance and Actinobacteria or Bacillota may indicate lineage-specific trade-offs under long-term energy limitation. The correlations highlight expression-based associations rather than definitive genomic co-localization or functional HGT.

Co-occurrence of ARGs with MTGs suggests shared selective pressures imposed by metal exposure and antibiotic resistance mechanisms. Trace metals are therefore more likely to structure microbial communities that harbor and express resistance genes than to directly select for ARGs. After multiple testing corrections, only a limited number of associations remained significant, indicating that many correlations are weak or context-dependent. Nevertheless, consistent trends in direction and magnitude suggest that trace metals exert diffuse, cumulative influences on microbial community structure and resistome composition.

Site-specific geochemical patterns further emphasize environmental heterogeneity. At TTH-04, multiple elements were associated with ARGs, MGEs, and MTGs, whereas fewer significant associations were observed at TTH-07 after FDR correction. The absence of significant MTG–environment associations at TTH-07 may reflect weaker coupling between metal resistance genes and environmental gradients or greater stochasticity in community assembly. Although many relationships did not remain significant after correction, their consistent directionality and moderate effect size suggests potential ecological relevance that warrants further investigation.

Elevated MTG levels beneath the active layers, particularly those associated with copper and mercury resistance, may reflect long-term geochemical selection pressures or historical co-selection processes. MGEs carrying both ARGs and MTGs may enhance microbial resilience under poly-extreme conditions by facilitating dual resistance and promoting gene exchange^80^, however, such functional linkages cannot be confirmed without genome-resolved data. Depth-resolved associations among ARGs, MGEs, and MTGs may therefore reflect a combination of contemporary geochemical gradients and ancient co-selection processes preserved since permafrost formation^4,76,80–84^. Procrustes analyses indicate that historical geochemistry contributes to observed ARG–MTG correlations, but these inferences are cautious given the limited number of cores.

Ecological modeling indicated that stochastic processes dominate ARG community assembly, with deterministic factors such as trace metal concentrations playing secondary roles. These dynamics suggest that permafrost microbiomes function as long-term, environmentally filtered systems^11,84,85^ in which resistome structure reflects the integration of historical legacy, constrained metabolic activity, and depth-dependent ecological differentiation. Network analysis revealed site-specific ecological strategies: TTH-07, characterized by deeper, more extreme conditions, supported a more competitive, bacteria–archaea–dominated network with prevalent negative interactions. In contrast, the comparatively milder conditions at TTH-04 fostered a more cooperative, bacteria–fungi-dominated assemblage. These contrasting topologies mirror local environmental stability and selective pressures^11,86–88^.

SEM analysis further revealed site-specific depth-related patterns. Other factors, such as ice content, redox history, or local mineralogy, may additionally influence ARG patterns, as reported in other permafrost studies^11,87,89,90^. At TTH-04, depth positively correlated with metabolic activity but negatively with phylogenetic diversity, suggesting that deeper layers support metabolically adapted communities shaped by long-term isolation and localized stressors^13,87^. Intriguingly, MTG abundance declined with depth, unlike Antarctic permafrost, indicating distinct metal deposition histories^91^. At TTH-07, ARG abundance increased with depth, consistent with Arctic permafrost and Antarctic soil studies where legacy contamination or ancient antibiotics shaped resistomes^19,92^. The inverse relationship between depth and MTGs may reflect energetic trade-offs under extreme nutrient limitation^93^.

Microbial phyla significantly influenced polymer-degrading genes, critical for accessing recalcitrant carbon (cellulose, lignin, and chitin) in deeper layers^17,73,94^. The opposing roles of metabolic genes, inhibiting ARGs while promoting MTGs (often due to essential cofactor requirements), echo findings in subglacial systems, where high metabolic activity suppresses resistance expression under starvation^95^. At TTH-07, the positive ARG–polymer-degrading gene linkage suggests co-selection, potentially via biofilm formation or shared regulatory pathways, as observed in landfill microbiomes^96,97^. Environmental factors emerged as dominant positive predictors of ARGs and metabolism-related genes, consistent with global permafrost meta-analyses^9^. Overall microbial diversity exerted a suppressive effect on ARGs, contrasting with patterns in temperate soils^98^, suggesting permafrost communities may prioritize survival over resistance.

Depth-resolved meta-transcriptomic profiling reveals a stratified resistome structured by long-term ecological filtering and environmental constraints. Although mobilization was not directly assessed, associations among ARGs, MGEs, and MTGs suggest latent connectivity that may become relevant under thaw conditions.

By integrating meta-transcriptomics with ecological modeling and network analyses, this study provides a framework for investigating resistome dynamics in deep permafrost and highlights thawing permafrost as a genetic archive with latent mobilization potential. These results provide rare RNA-based insights into transcriptionally detectable microbial communities at extreme depths and reveal site-specific ecological patterns that warrant further investigation.

Several limitations should be acknowledged. Logistical constraints restricted spatial replication and sampling resolution. Nevertheless, the 40 m and 100 m RNA-preserved cores represent some of the deepest biologically interrogated permafrost samples to date. The study sites are located in continuous permafrost within the Tuotuo River Basin, where permafrost generally acts as a strong hydrological barrier limiting vertical infiltration beyond the active layer; nevertheless, subsurface hydrological connectivity cannot be completely excluded in heterogeneous permafrost systems. While taxonomic profiling of ARG transcripts identifies likely contributors to resistance gene expression, the absence of functional assays, genome-resolved metagenomics, or long-read assemblies precludes definitive assignment of ARGs to chromosomal or plasmid origins. Limited sample biomass and inconsistent DNA recovery also prevented reliable qPCR- or metagenome-based estimates of absolute microbial abundance. Consequently, interpretations are based on transcriptional signals and should be understood as reflecting relative expression patterns rather than absolute microbial activity or abundance. Despite strict cold-chain handling, RNA signals may represent a mixture of active transcription, preserved extracellular or intracellular remnants, and stress-induced responses. Therefore, interpretations are made at the level of transcriptional potential rather than direct measurement of real-time activity at the moment of sampling. Future studies integrating multi-omics approaches, functional validation, and statistical frameworks tailored to sparse and zero-inflated microbiome data (e.g., Bayesian or zero-inflated negative binomial models) across multiple independent cores will be essential to more rigorously resolve associations between ARGs, microbial taxa, and environmental drivers and to assess their ecological and public health implications in a warming world.

## Methods

### Study sites and sample collection

A permafrost drilling campaign was conducted in the Tuotuo River region of the central QTP during the peak freezing season (October–November 2020; Supplementary Fig. 8; Supplementary Methods 1.1). A total of 25 stratified soil subsamples were collected from two representative deep permafrost cores: TTH-04 (91°30′3.71″E, 34°3′55.10″N; elevation: 4850 m) and TTH-07 (91°21′21.32″E, 34°2′48.98″N; elevation: 5005 m). Due to the logistical constraints of deep drilling (>40–100 m) at high elevation and the requirement to maintain aseptic cryogenic conditions for RNA preservation, only two independent permafrost cores were obtained and processed for meta-transcriptomic analyses. Core depths reached 40 m at TTH-04 and extended to 100 m at TTH-07^99,100^. Of the 25 samples, 10 were obtained from TTH-04 and 15 from TTH-07, distributed across the active layer, permafrost, and sub-permafrost horizons (Supplementary Table 4). The number of samples within each depth-defined horizon was not uniform due to logistical constraints associated with deep drilling and core recovery. Consequently, samples within each horizon represent pseudo-replicates within individual cores rather than independent biological replicates across sites. Statistical analyses were therefore designed to evaluate within-core, depth-resolved patterns rather than fully balanced comparisons among horizons or between cores. Although samples were grouped into depth-defined horizons based on cryostratigraphic and thermal criteria, depth was also evaluated as a continuous variable in correlation analyses to assess gradient-based relationships with ARG expression and associated features.

TTH-04 is located within a low solar-exposure basin characterized by gentle slopes (<4%), dense vegetation cover (>90%), and breccia-dominated substrates. In contrast, TTH-07 lies on a piedmont slope with higher solar exposure, moderate vegetation cover (~55%), and gravel-rich soil profiles (Supplementary Fig. 8). Despite these surface differences, both sites exhibit relatively flat topography (slopes < 5%) and are underlain by continuous permafrost.

To minimize contamination and disturbance-related artefacts, cores were recovered using a triple-tube rotary drilling system under frozen conditions. Immediately after retrieval, only the inner core material was used for downstream analyses following removal of the outer 1–2 cm using sterile tools. All subsampling was conducted under continuous frozen handling to minimize thaw-induced transcriptional responses, oxygen exposure, and mechanical redistribution of microbial material. Field and process blanks were included to monitor potential contamination, and laboratory processing was conducted under strict RNase/DNase-free sterile conditions in a biosafety cabinet (Supplementary Methods 1.1).

Permafrost stratification was determined using borehole thermal profiling, field cryostratigraphy, and ice-content observations. Samples were classified into three depth-defined horizons: the active layer (seasonally thawed surface soil), permafrost (perennially frozen sediments), and sub-permafrost (deeper unfrozen strata influenced by geothermal heat)^27,101^. Thermal profiles of borehole profiling and excavation confirmed that the upper horizons were seasonally thawed and declared active layer (2.4 m in TTH-04 and 2.3 m in TTH-07), followed by perennially frozen permafrost layers (<0 °C) extending to ~19 m (TTH-04) and ~73 m (TTH-07). Sub-permafrost transitions, where temperatures gradually rose above 0 °C due to geothermal heating, were identified at 20 m and 74 m, respectively (Supplementary Table 4). Ice lenses were observed within both vertical profiles (Supplementary Fig. 8). The permafrost cores represent heterogeneous, ice-rich systems with surface layers containing visible organic material and deeper silty, sandy, and sandstone units exhibiting variable cryostructures (e.g., layered ice, ice lenses, and inclusions), reflecting pronounced vertical heterogeneity in sediment composition and ground ice distribution. These characteristics reflect strong vertical heterogeneity in sedimentary and cryogenic processes. Detailed stratigraphic, lithological, and geochemical descriptions are provided in Supplementary Table 4.

Downhole temperature profiles were recorded during drilling. Field observations did not indicate large-scale cryoturbation; however, fine-scale or historical cryogenic mixing processes cannot be excluded. No direct geochronological dating was performed; therefore, depth is used as a relative stratigraphic proxy rather than an absolute temporal framework. Samples were stored at −40 °C in the field and transported under continuous frozen conditions to the laboratory for analysis.

### Physicochemical analysis

Soil pH was measured in a 2:1 (m v^−1^) soil-to-ultrapure water suspension using a calibrated digital pH meter (Model 361, Thermo Fisher Scientific), following standard soil analysis protocols (e.g., ISO 10390)^102^. For elemental and trace metal analyses, freeze-dried soils were subjected to microwave-assisted acid digestion (HNO₃–HF–H₂O₂) using a MARS Xpress system (CEM, USA), following established protocols for environmental solids (e.g., EPA Method 3052)^103^. Extracts were quantified using inductively coupled plasma mass spectrometry (ICP-MS) following certified analytical protocols at the Public Technology Service Center. Quality assurance included standard reference materials, matrix spikes, and procedural blanks. Total nitrogen was also measured as part of the soil chemical characterization^38^.

### RNA extraction, sequencing and meta-transcriptomic analysis

To minimize contamination, the outer 1–2 cm of each core segment was aseptically removed before subsampling. Preliminary DNA extractions from deep permafrost yielded inconsistent or insufficient quantities; therefore, all available material was allocated to RNA-based analyses. Total RNA was extracted from 0.5 g of homogenized soil using TRIzol (Invitrogen, Cat. No. 15596026) (Supplementary Table 5). Residual DNA was removed via DNase treatment, and RNA was reverse-transcribed into cDNA using the HiScript® Reverse Transcriptase kit (Vazyme Biotech, Cat. No. R101-01) with random hexamer primers to capture microbial transcripts, including non-polyadenylated mRNA (see Supplementary Methods 1.3 for rationale). No-RT controls and extraction blanks were included to monitor contamination (Supplementary Methods 1.2).

Sequencing libraries were prepared according to the manufacturer’s standard protocols for low-input RNA samples and sequenced on an Illumina NovaSeq 6000 platform (paired-end 150 bp) by BENAGEN Co., China^38^. Libraries were sequenced at high depth (60–100 million reads per sample) to ensure sufficient detection sensitivity for low-abundance transcripts in these low-biomass samples (Supplementary Methods 1.3). No rRNA depletion or poly(A) enrichment was performed due to the low RNA yields and the need to retain non-polyadenylated microbial transcripts (Supplementary Methods 1.3). Raw reads were processed using FASTP (default parameters), and high-quality reads were assembled de novo using MEGAHIT (v1.2.9) with a multi–k-mer strategy (*k* = 21–141) and a minimum contig length of 500 bp, which allows improved assembly of both low-abundance and longer transcripts by balancing sensitivity and specificity (Supplementary Methods 1.3). Reads were mapped to the Structured Antibiotic Resistance Gene (SARG) database v2.0 using Bowtie2 (v2.4.5)^104^, and transcript abundance was quantified and normalized as TPM using Salmon. TPM normalization accounts for both gene length and sequencing depth, enabling comparison of relative transcript abundances across samples (Supplementary Fig. 9). However, TPM values represent relative expression within the sequenced transcript pool and do not provide a measure of absolute transcript abundance or total transcriptional activity.

Nineteen ARG classes were detected based on functional annotation and measurable transcript abundance. These classes represent a subset of the ~38 ARG categories defined in the SARG v2.0 databases and include aminoglycosides, β-lactams, macrolide–lincosamide–streptogramin (MLS), multidrug resistance, and others. ARGs were further annotated by resistance mechanism. Contigs were classified as plasmid- or chromosome-associated using PlasFlow^105^, recognizing that such assignments from transcriptomic assemblies represent putative genomic context rather than resolved localization.

### Annotation of MGEs, MTGs, and functional genes

MGEs, including integrases and transposases, were identified via BLASTN against RefSeq v89 and ISfinder (2019-09-25) using thresholds of ≥60% identity and ≥60% coverage^106^. Integrative and conjugative elements were detected using the ICEberg database (identity ≥ 80%, coverage ≥ 50%)^107^. Detected transcripts primarily represent structural, regulatory, or maintenance-associated genes within these element families (details characteristics of MGEs are provided in Supplementary Data 1 and 2). Because only transcriptomic data were available, these annotations indicate potential activity rather than confirmed excision, integration, or horizontal gene transfer.

Additional functional annotations targeted natural polymer-degrading enzymes (PMBD, PlasticDB) and metal tolerance genes (MTGs) using the BacMet database^108^. Taxonomic classification was performed using Kraken2 with Bracken for abundance refinement, and functional pathways were reconstructed using KEGG orthology (Supplementary Methods 1.3).

### Statistical analysis and reproducibility

All analyses were conducted using a multi-platform computational workflow. Relative abundance analyses, Sankey diagrams, and circular packing plots were generated using OriginPro 2024b. Multivariate analyses, including principal coordinates analysis (PCoA; Bray–Curtis dissimilarity on TPM-normalized data), Procrustes tests (comparison of Bray–Curtis-based ordinations), ternary plots, Pearson correlations (two-tailed), Mantel tests (Bray–Curtis, 9999 permutations), and redundancy analysis (RDA), were conducted via the Tutools cloud platform (https://www.cloudtutu.com). Circos plots were produced using the Majorbio Cloud Platform (https://cloud.majorbio.com/page/tools/). Statistical significance was defined at *α* = 0.05 and *p*-values were adjusted for multiple comparisons using the Benjamini–Hochberg false discovery rate (FDR) correction.

Co-occurrence networks ( | *r* | ≥ 0.6 or *r* ≤ −0.6; *p* ≤ 0.05)^109^ were constructed based on Pearson correlations. To reduce spurious associations and improve robustness, edges were pre-filtered using these thresholds prior to multiple testing correction, resulting in a reduced set of high-confidence associations subjected to Benjamini–Hochberg FDR adjustment. Network topology, including modularity and graph density, was calculated using the psych package in R v4.1.2, and network visualization was performed in Gephi v0.10.1 (Fruchterman–Reingold layout)^110^. Community assembly processes were modeled using NCM, and variable interactions were assessed through SEM with lavaan and semPlot packages in R (model fit indices: *χ*² test, RMSEA ≤ 0.08, CFI ≥ 0.90). Community comparisons included ANOSIM (ARG composition), PERMANOVA (Adonis) (community structure), and Permidisp (variability across permafrost layers). Differential ARG expression across layers was evaluated in STAMP using Wilcoxon rank-sum tests with Benjamini–Hochberg FDR correction^111^.

Due to the substantial logistical challenges associated with deep permafrost RNA sampling, this study included two independent permafrost cores (TTH-04, 40 m; *n* = 10 depth-resolved subsamples, and TTH-07, 100 m; *n* = 15 depth-resolved subsamples). Within each core, multiple stratified subsamples from the active layer, permafrost, and sub-permafrost horizons were treated as replicate units to evaluate within-core, depth-resolved patterns. All analyses (including diversity metrics, PERMANOVA, Mantel and Procrustes tests, differential expression, and network correlations) were performed on these depth-based groups to evaluate within-core, depth-resolved patterns, with interpretations explicitly limited to site-specific observations rather than regional generalization (Supplementary Methods 1.4). The consistency of stratified patterns observed across both independent cores provides supportive evidence for the reproducibility of the identified depth-related trends.

### Reporting summary

Further information on research design is available in the Nature Portfolio Reporting Summary linked to this article.

## Supplementary information

Supplementary Materials

Description of Additional Supplementary files

Supplementry Data 1

Supplementry Data 2

Supplementry Data 3

Supplementary Data 4

Supplementary Data 5

Reporting Summary

Transparent Peer Review file

## Data Availability

The sequencing data generated in this study are available through the NCBI BioProject database under accession number PRJNA1251305. Mobile genetic element (MGE) identification and annotation data for permafrost cores TTH-04 and TTH-07 are provided in Supplementary Data 1 and Supplementary Data 2, respectively. Trace metal concentration data and results of Kruskal–Wallis tests followed by Dunn’s post hoc multiple comparison analyses are provided in Supplementary Data 3. Numerical source data underlying all graphs and charts presented in the main figures are provided in Supplementary Data 4, and numerical source data underlying all Supplementary Figs. and additional datasets related to metabolism genes, microbial phyla, and polymer-degrading genes are provided in Supplementary Data 5. Additional sample metadata and supporting information are available in the Supplementary Information. All other data supporting the findings of this study are available from the corresponding author upon reasonable request.
