# Supplementary material for: Depth-resolved transcriptional activity of antibiotic resistance genes in deep permafrost of the Qinghai–Tibet Plateau
Source: Commun Biol. 2026 Jun 30;9:1374. doi: 10.1038/s42003-026-10593-2 (PMC13683339; doi:10.1038/s42003-026-10593-2)
Supplement: Supplementary file 1 [file 42003_2026_10593_MOESM1_ESM.pdf]

Supplementary Informations

Depth-resolved transcriptional activity of antibiotic resistance genes in deep permafrost of the Qinghai–Tibet Plateau

Wasim Sajjad[1], Shichang Kang[1,2]*, Xiufeng Yin[1], Xiaodong Wu[1], Fei Deng[3], Christopher Williamson[4], Muhammad Rafiq[5,6].

[1]State Key Laboratory of Cryospheric Science and Frozen Soil Engineering, Northwest Institute of Eco-Environment and Resources, Chinese Academy of Sciences, Lanzhou 730000, China.

[2]Institute of Mountain Hazards and Environment (IMHE), Chinese Academy of Sciences, Chengdu, 610213, China.

[3]Key Laboratory of Virology and Biosafety and National Virus Resource Center, Wuhan Institute of Virology, Chinese Academy of Sciences, 430071 Wuhan, China.

[4]Bristol Glaciology Centre, School of Geographical Sciences, University of Bristol, Bristol, BS8 1SS, United Kingdom.

[5]Guizhou Key Laboratory of Microbiome and Infectious Disease Prevention and Control & Key Laboratory of Environmental Pollution Monitoring and Disease Control, Ministry of Education of Guizhou & School of basic Medical Sciences & Institution of One health research, Guizhou Medical University, Guiyang, Gui`an New district, 561113, China.

[6]Department of Microbiology, Faculty of Life Sciences and Informatics, Balochistan University of Information Technology, Engineering and Management Sciences, Quetta, 87300, Pakistan.

*Corresponding author: Shichang Kang.

Email: shichang.kang@lzb.ac.cn

1. Supplementary Methods

1.1 Sample Collection and Contamination Control

Permafrost drilling and sampling were conducted in the Tuotuo River Basin, central Qinghai–Tibet Plateau, during the peak freezing season (October–November 2020) to minimize thaw-related disturbance and potential exogenous microbial input. Two representative sites were selected: TTH-04 (40 m core; 4850 m elevation) and TTH-07 (100 m core; 5005 m elevation), capturing contrasting geomorphological settings and surface conditions.[1,2]

A total of 25 stratified subsamples were obtained from the two cores, with samples distributed across three depth-defined horizons (active layer, permafrost, and sub-permafrost). The number of samples within each horizon differed between cores due to drilling constraints and core recovery conditions (Supplementary Table 4). As such, depth-defined groups represent pseudo-replicates within individual cores rather than independent biological replicates. Statistical analyses were therefore designed to assess within-core depth-related patterns rather than to perform fully balanced comparisons across horizons or between sites.

Both sites are underlain by continuous permafrost, with active layer thicknesses of approximately 2.3–2.4 m determined by borehole thermal logging and pit excavation. Stratigraphic classification into active layer, permafrost, and sub-permafrost zones was based on field cryostratigraphy, in situ thermal gradients, and ice content observations.[3,4] Ice lenses, soil texture, and color transitions were documented in situ to confirm cryogenic stratification. No direct radiometric dating was performed; therefore, depth is used as a relative stratigraphic proxy rather than an absolute temporal indicator.

Drilling was carried out using a rotary drilling rig equipped with a triple-tube coring system designed to preserve stratigraphy and minimize mechanical disturbance. Drill bits, liners, and associated equipment were sterilized with 70% ethanol between core segments. To further reduce contamination risk, field processing was performed while maintaining cores in a frozen state, thereby minimizing temperature fluctuations, oxygen exposure, and potential thaw-induced microbial activation. Although drilling and recovery inevitably impose mechanical and environmental perturbations, all subsampling was conducted immediately under frozen conditions to reduce transcriptional responses associated with thaw or handling.

The outer 1–2 cm of each core was removed using sterile scalpels to minimize potential surface contamination. Inner-core material (Untouched part of the core) was then subsampled using sterile, single-use instruments under frozen conditions. Subsampling was performed on dry ice in a pre-cooled laboratory to prevent thaw-induced biological activity or transcriptional changes. Although tracer-based penetration tests were not performed, the protocol follows established standards for

permafrost microbiology and low-biomass subsurface sampling. The mechanical scraping approach used for outer-layer removal is widely applied in permafrost microbiology; however, it is recognized that no decontamination method can fully eliminate all contamination risks in low-biomass systems. To mitigate known limitations such as potential microfracture-mediated contamination transport reported in previous studies[5], only the uncontaminated interior core was used for RNA extraction, and strict sterile handling procedures were maintained throughout. Samples were sealed in sterile Whirl-Pak bags, placed in pre-sterilized insulated containers, and transported at ≤–20°C (temperature loggers were used) to ensure uninterrupted cold-chain conditions from field to laboratory. Long-term storage before RNA extraction was at –80°C.

Field workflows included sterility blanks (sterile collection tubes exposed at the drilling site) and negative controls (sterile ddH_2O processed in parallel), which were included to monitor potential contamination during field handling. Laboratory contamination controls included extraction blanks and no-reverse-transcription (no-RT) controls. All negative controls showed no detectable nucleic acids and no amplification signals after DNase treatment, indicating minimal contamination within detection limits.

Laboratory processing was conducted at the Northwest Institute of Eco-Environment and Resources (Chinese Academy of Sciences, Lanzhou) within a Class II biological safety cabinet using DNA/RNA-free consumables.[6] Sterile, single-use instruments were employed for each depth horizon to reduce cross-contamination. These procedures followed established permafrost microbiology standards (e.g., AMAP 2017 guidelines; ISO 14698:1999), ensuring preservation of in situ microbial assemblages, nucleic acids, and geochemical signatures.

Due to logistical constraints associated with deep drilling and RNA preservation at high elevation, only two deep permafrost cores were collected (TTH-04: 40 m; TTH-07: 100 m) for RNA-based analyses. Consequently, biological replication at the landscape scale is limited, and all results should be interpreted as site-specific, depth-resolved observations rather than region-wide generalizations. Each site represents a distinct combination of environmental conditions (e.g., vegetation cover, solar exposure, substrate composition), and environmental differences are therefore confounded with core identity. As a result, the effects of depth cannot be statistically disentangled from site-specific variation.

Within each core, multiple stratified subsamples were collected from three depth-defined horizons (active layer, permafrost, and sub-permafrost). These stratified intervals represent pseudo-replicates within individual cores rather than independent biological replicates. Depth-defined groups were treated as replicate units for statistical analyses to enable robust evaluation of within-core, depth-resolved patterns in ARGs, MGEs, MTGs, microbial communities, and trace elements.

Downhole temperature measurements were recorded during drilling using a digital thermistor probe, providing depth-resolved thermal profiles for each core. Layers were classified following standard cryostratigraphic criteria: (i) active layer， sediments

with temperatures above or oscillating around 0 °C; (ii) permafrost， sediments with persistently subzero temperatures; and (iii) sub-permafrost， deeper strata where temperatures rise above 0 °C due to geothermal heat flux. Thermal profiles for both sites are provided in Supplementary Table 4.

The studied cores were recovered from continuous permafrost within the Tuotuo River Basin on the Qinghai–Tibet Plateau. In such systems, permafrost is generally characterized as an aquiclude having low permeability due to ice-rich conditions[7,8], which constrains vertical water movement and promotes hydrological processes largely confined to the active layer. As a result, supra-permafrost flow is typically dominant, while vertical infiltration into deeper permafrost horizons is limited. However, direct hydrological measurements were not performed in this study, and subsurface connectivity cannot be completely excluded, particularly in the presence of heterogeneity, taliks, or localized disturbance.

With respect to cryoturbation and paleo–active layer dynamics, field observations during drilling did not reveal clear evidence of strongly disrupted bedding or large-scale cryoturbation features within the sampled cores. Nevertheless, finer-scale or historical mixing processes may occur in permafrost environments and cannot be excluded. Such processes could result in limited vertical redistribution of organic material or microbial signatures.

Furthermore, although permafrost depth is often used as a proxy for temporal separation, the absence of direct geochronological constraints (e.g., radiocarbon or luminescence dating) introduces uncertainty into the absolute age of the sampled horizons. In addition, past hydrological processes, cryogenic mixing, and regional glacial or periglacial dynamics may have influenced sediment deposition and preservation. Therefore, depth-resolved transcriptomic patterns should be interpreted with caution, as they may not exclusively reflect strictly stratified or temporally discrete microbial communities.

1.2 RNA Extraction, DNA removal, and contamination controls

Total RNA was extracted using TRIzol™ reagent (Invitrogen) and quantified with the Qubit RNA High Sensitivity (HS) Assay Kit (Thermo Fisher Scientific, Q32852) on a Qubit 4 Fluorometer according to the manufacturer's instructions (Supplementary Table 5). Extracted RNA concentrations ranged from 4.1–7.7 ng μL^{-1} (mean = 6.1 ng μL^{-1}).

To minimize genomic DNA contamination, all RNA extracts were treated with RNase-free DNase I prior to reverse transcription. Residual DNA was evaluated using the Qubit DNA HS Assay and was either below the detection limit or represented <5% of total nucleic acid signal following DNase treatment.

Contamination was assessed using multiple negative controls throughout the workflow. These included extraction blanks (RNase-free water processed through the full TRIzol extraction protocol) and no-reverse-transcriptase (no-RT) controls included during cDNA synthesis to evaluate potential DNA carryover and contamination introduced during reverse transcription. No detectable nucleic acids were observed in extraction blanks using Qubit assays, indicating the absence of measurable background contamination during RNA extraction. In addition, no amplification signal was detected in no-RT controls following PCR amplification, demonstrating effective removal of genomic DNA by DNase treatment and confirming that residual DNA did not contribute detectably to downstream cDNA libraries. Although these controls were not subjected to sequencing due to insufficient nucleic acid yield (Supplementary Table 5), the absence of detectable nucleic acids and amplification suggests that background contamination and DNA-derived artefacts were below the detection limits of the applied experimental assays.

Permafrost core subsampling and RNA extraction were conducted under strict cold-chain and RNase-free conditions. Core sections were maintained frozen during transport and storage, and subsampling was performed on dry ice in a pre-cooled laboratory environment to prevent thaw-induced biological activity. No incubation or thawing period was permitted prior to RNA stabilization. Total RNA was extracted immediately after subsampling using protocols optimized for low-biomass and environmentally challenging cryogenic samples, which are commonly applied in permafrost and subsurface microbiome studies[9,10]. All procedures were performed using RNase-free consumables and reagents to minimize RNA degradation and contamination.

1.3 Sequencing and meta-transcriptomic analysis

Reverse transcription was performed using the HiScript® Reverse Transcriptase kit (Vazyme Biotech, Cat. No. R101-01) according to the manufacturer's instructions, using random hexamer primers rather than oligo(dT) to enable comprehensive capture of microbial transcripts, as most bacterial and archaeal mRNA lacks poly(A) tails. Briefly, RNA was reverse transcribed in 20 μL reactions containing 50 ng random hexamers, 1 mM dNTPs, 200 U HiScript RT and RNase inhibitor. Reactions were incubated at 25°C for 10 min (primer annealing), 50°C for 30 min (cDNA synthesis), then 85°C for 5 min (enzyme inactivation). cDNA was stored at −20°C prior to library preparation. Sequencing libraries were prepared and run on an Illumina NovaSeq platform (paired-end, 150 bp; ~10 Gb

Libraries were sequenced to a depth of approximately 60–100 million reads per sample. This relatively high sequencing depth was intentionally employed to compensate for the extremely low RNA biomass typical of deep permafrost samples

and to ensure sufficient detection sensitivity for low-abundance transcripts. Deep sequencing improves transcript recovery and reduces stochastic sampling effects in low-biomass environmental transcriptomic datasets. Variability in GC content likely reflects depth-dependent differences in microbial community composition and taxonomic heterogeneity across stratified horizons. In addition, minor technical contributions cannot be excluded, including library preparation and amplification biases that may differentially affect GC-rich or GC-poor templates, as well as differences in read composition following quality filtering steps. Given the low-biomass nature of deep permafrost samples, such variability is expected in metatranscriptomic datasets and does not necessarily indicate contamination or systematic sequencing error.

Raw reads (Supplementary Table 1) were processed using FASTP to remove adapters, low-quality bases, and short reads (<25 bp). Quality-filtered reads were inspected using FastQC v0.11.9. No rRNA depletion or mRNA enrichment step was performed, due to extremely low RNA yields from deep permafrost and the need to retain non-polyadenylated transcripts from bacteria, archaea, and fungi. Clean reads were mapped against SILVA rRNA databases, and rRNA-derived reads were removed prior to downstream analyses. Only high-quality non-rRNA reads were used for assembly and ARG/MGE/MTG detection. High-quality reads were assembled *de novo* with MEGAHIT (v1.2.9; k-mer range: 21–141; minimum contig length: 500 bp) (Supplementary Table 6; Supplementary Fig. 9). Identification of antibiotic resistance genes (ARGs) was performed using a read-based approach, providing a direct measure of expressed resistance functions independent of assembly bias. Clean reads were aligned to the Structured Antibiotic Resistance Gene (SARG) database v2.0 (http://smile.hku.hk/SARGs/) using Bowtie2 (v2.4.5), and alignments were processed with Samtools (v1.13) to extract mapped sequences. Novel ARGs were identified from meta-transcriptomic reads using a read-based approach with the SARG 2.0 database. Reads were aligned to the SARG 2.0 reference using stringent thresholds (≥80% identity, ≥70% coverage). Reads that mapped to conserved resistance-associated protein domains but lacked significant matches to known ARG sequences were classified as putative novel ARGs. This approach allows detection of previously uncharacterized resistance genes based on conserved functional motifs, even in the absence of full-length homologs in reference databases.

ARG expression levels were normalized using transcripts per million (TPM) using Salmon to correct for gene length and sequencing depth. Because metagenomic data were not available, results are interpreted as relative transcriptional activity rather than expression normalized to genomic ARG abundance. ARGs were classified by resistance class and mechanism based on SARG annotations. ARGs were classified by drug class, including β-lactams (penams, cephalosporins, carbapenems, cephamycins, and monobactams), macrolide-lincosamide-streptogramin (MLS), multidrug, and aminoglycoside resistance genes (major reference databases describe approximately

30–40 ARG classes). Contigs were further annotated for plasmid or chromosomal origin using PlasFlow[11] with default parameters.

Mobile genetic elements (MGEs), including integrases, transposases and insertion sequences (IS), were annotated by BLASTN searches of contigs against RefSeq v89 and ISfinder (2019-09-25) (≥60% identity, ≥60% coverage). Integrative and conjugative elements (ICEs) were detected using ICEberg (≥80% identity, ≥50% coverage). Detected transcripts primarily correspond to structural or regulatory components of these elements and do not constitute direct evidence of active mobilization. Additional annotations targeted natural polymer-degrading enzymes (PMBD, PlasticDB) and metal resistance genes (BacMet). Taxonomic assignments were performed using Kraken2, with Bracken for abundance refinement, and functional pathways were reconstructed via KEGG orthology.

1.4 Statistical analysis framework

Given that only two deep permafrost cores were sampled, statistical comparisons between sites are not appropriate. All depth-resolved analyses treat stratified subsamples within each core as replicate units, allowing robust assessment of site-specific trends, but inter-site differences are presented as descriptive observations only. We note that with only two deep permafrost cores, it is not possible to distinguish definitively between generalizable depth-dependent effects, site-specific environmental influences, or stochastic variation. Thus, our observations should be interpreted as depth-resolved, site-specific patterns, which provide rare empirical insights into deep permafrost resistomes and can guide future multi-site investigations.

Neutral Community Models (NCMs) evaluate the extent to which community assembly patterns can be explained by stochastic processes, primarily random dispersal and ecological drift, rather than deterministic environmental selection. In this study, NCMs were used to assess whether ARG, MTG, and MGE distributions follow neutral expectations or show deviations suggesting selective pressures.

Structural Equation Modeling (SEM) is a multivariate statistical framework that evaluates hypothesized causal relationships among variables. SEM allows simultaneous testing of direct and indirect pathways. Here, SEM was used to quantify how environmental factors, microbial community composition, and mobility elements jointly influence resistome structure.

Because correlations were pre-filtered prior to FDR correction (based on effect size and nominal significance), the proportion of retained significant associations reflects both relatively stronger effect sizes and a reduced multiple testing burden.

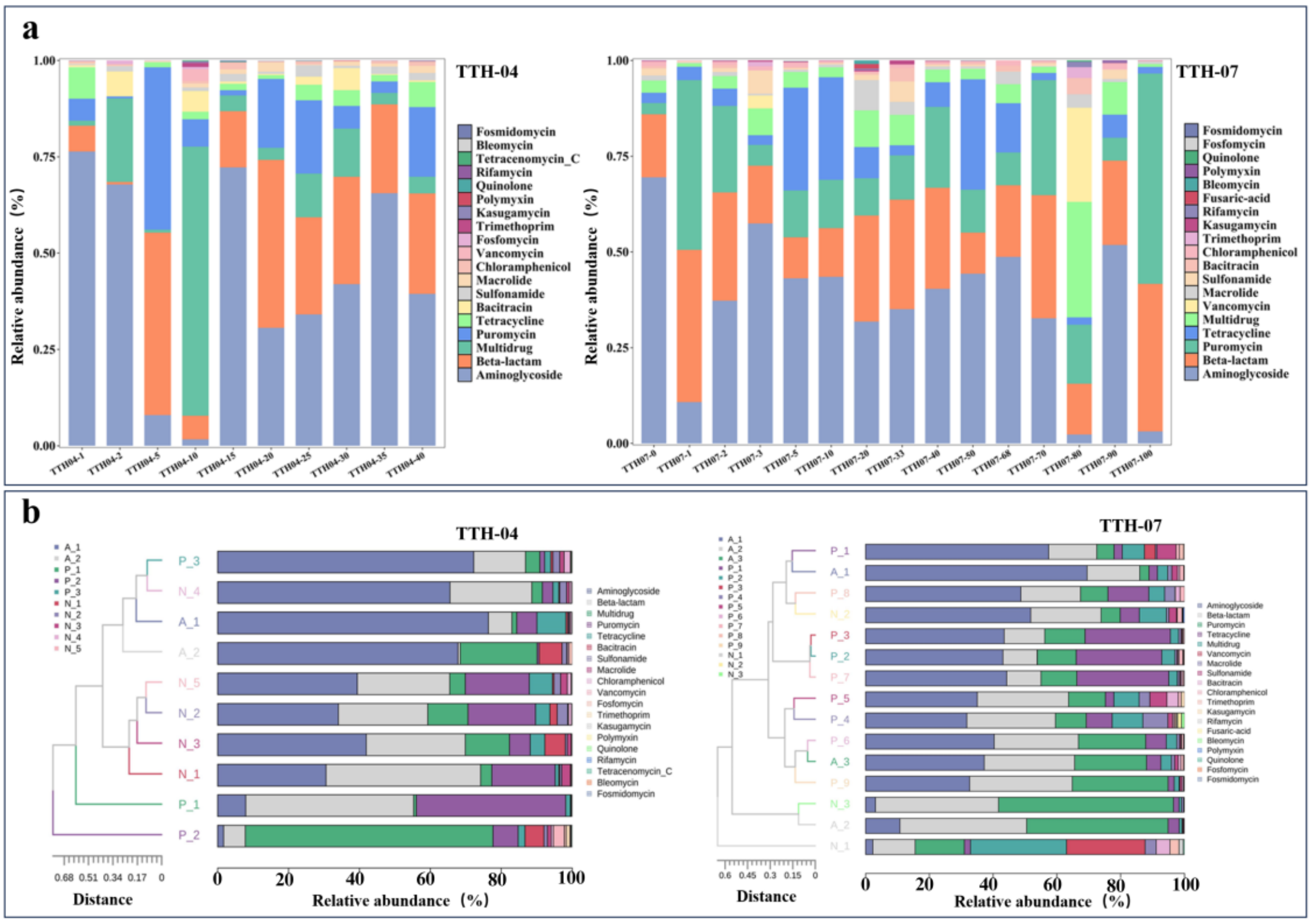

Supplementary Fig. 1. Expression Profiles of Antibiotic Resistance Genes (ARGs) Across Permafrost Layers at Sites TTH-04 and TTH-07. (a) Bar graph showing the relative abundance (%) of 19 ARG classes in individual core samples from TTH-04 (n = 10) and TTH-07 (n = 15). (b) Structural dynamics of ARG classes across individual samples at varying depths. Sample codes correspond to depth categories: TTH-04: A_1 = 1 m, A_2 = 2 m, P_1 = 5 m, P_2 = 10 m, P_3 = 15 m, N_1 = 20 m, N_2 = 25 m, N_3 = 30 m, N_4 = 35 m, N_5 = 40 m.TTH-07: A_1 = 0 m, A_2 = 1 m, A_3 = 2 m, P_1 = 3 m, P_2 = 5 m, P_3 = 10 m, P_4 = 20 m, P_5 = 33 m, P_6 = 40 m, P_7 = 50 m, P_8 = 68 m, P_9 = 70 m, N_1 = 80 m, N_2 = 90 m, N_3 = 100 m. "A" denotes the Active layer, "P" represents the Permafrost layer, and "N" indicates the Sub-permafrost layer.

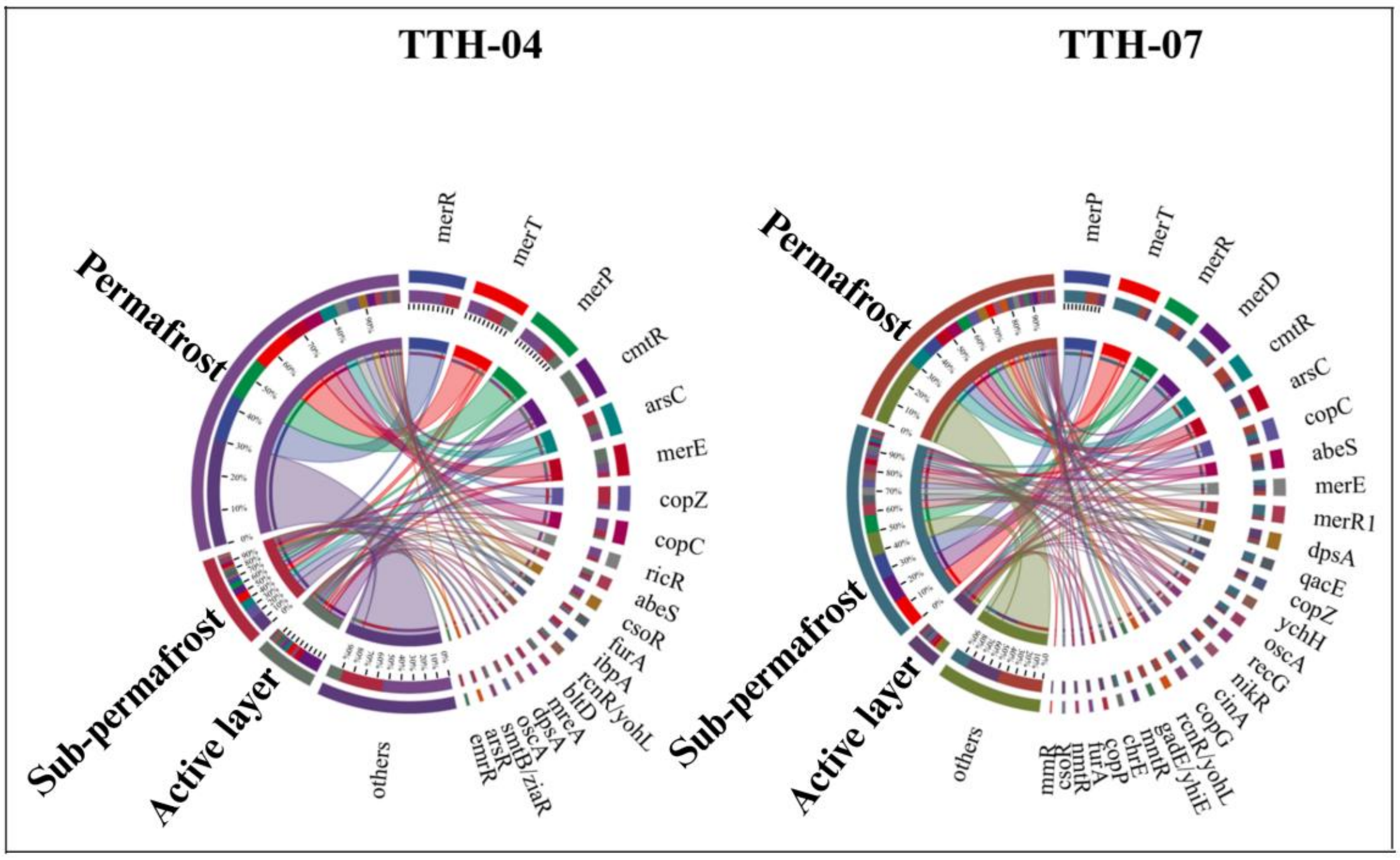

Supplementary Fig. 2. Relative Abundance and Distribution of Metal Tolerance Genes (MTGs) Across Permafrost Cores at Sites TTH-04 and TTH-07. The inner left side of the circle illustrates the relative abundance of MTGs across the three permafrost layers: Active layer, Permafrost layer, and Sub-permafrost layer. The outer circle represents the permafrost stratification, while the inner circle indicates the percentage distribution of MTGs within each layer. On the right side, the outer circle denotes the identified MTGs, while the inner circle represents their distribution across the permafrost layers.

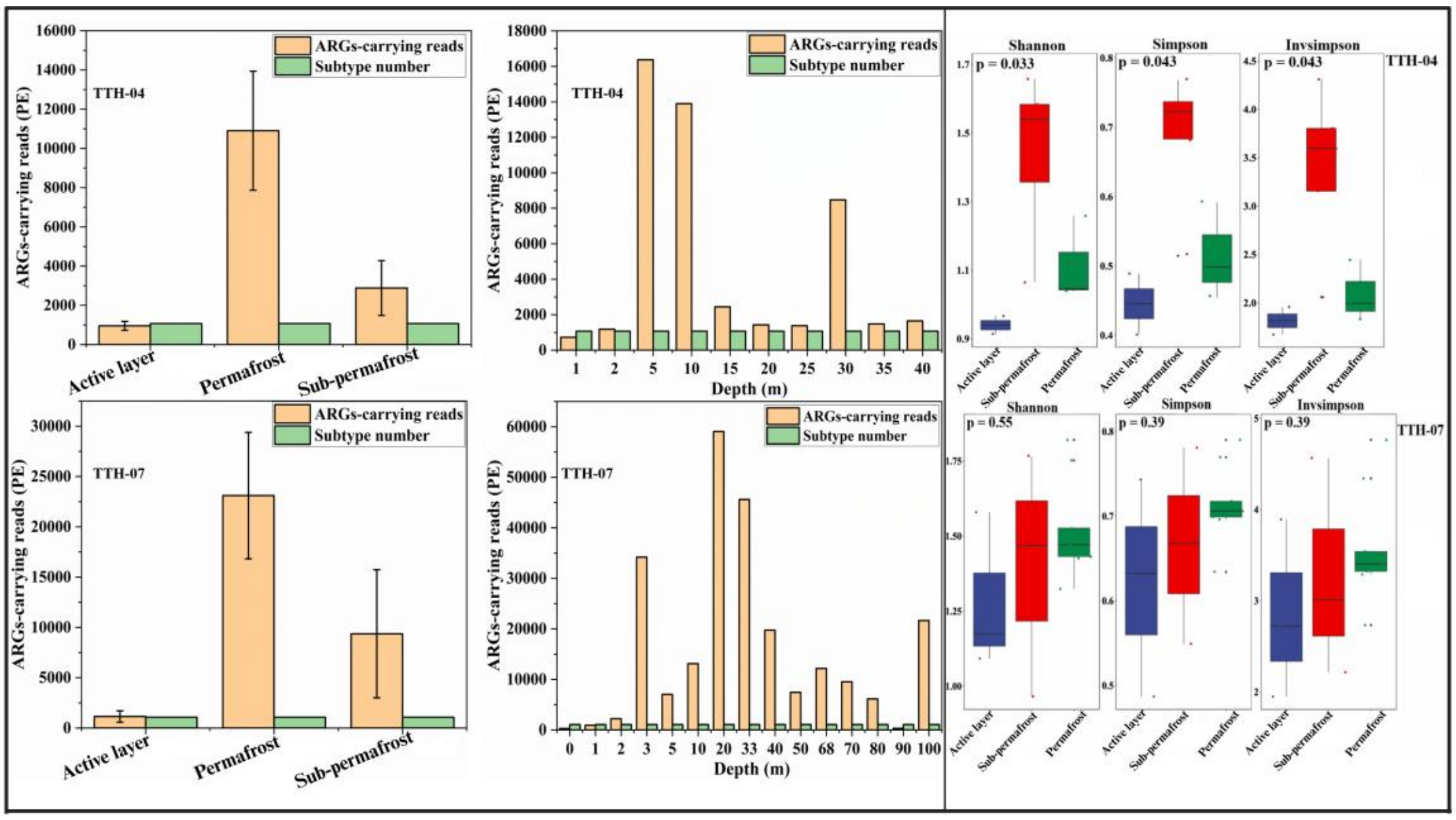

Supplementary Fig. 3. Absolute Abundance of Expressed ARG-carrying Reads and Alpha-diversity Indices of Antibiotic Resistance Genes (ARGs) Across the Active Layer, Permafrost, and Sub-permafrost Layers at sites TTH-04 and TTH-07. The analysis included 1,074 ARG subtypes classified into 22 major categories. Alpha-diversity indices (Shannon, Simpson, and Inverse Simpson) were calculated to assess ARG abundance across layers, with statistical significance evaluated using the Kruskal-Wallis test with Benjamini-Hochberg at 0.95 confidence intervals.

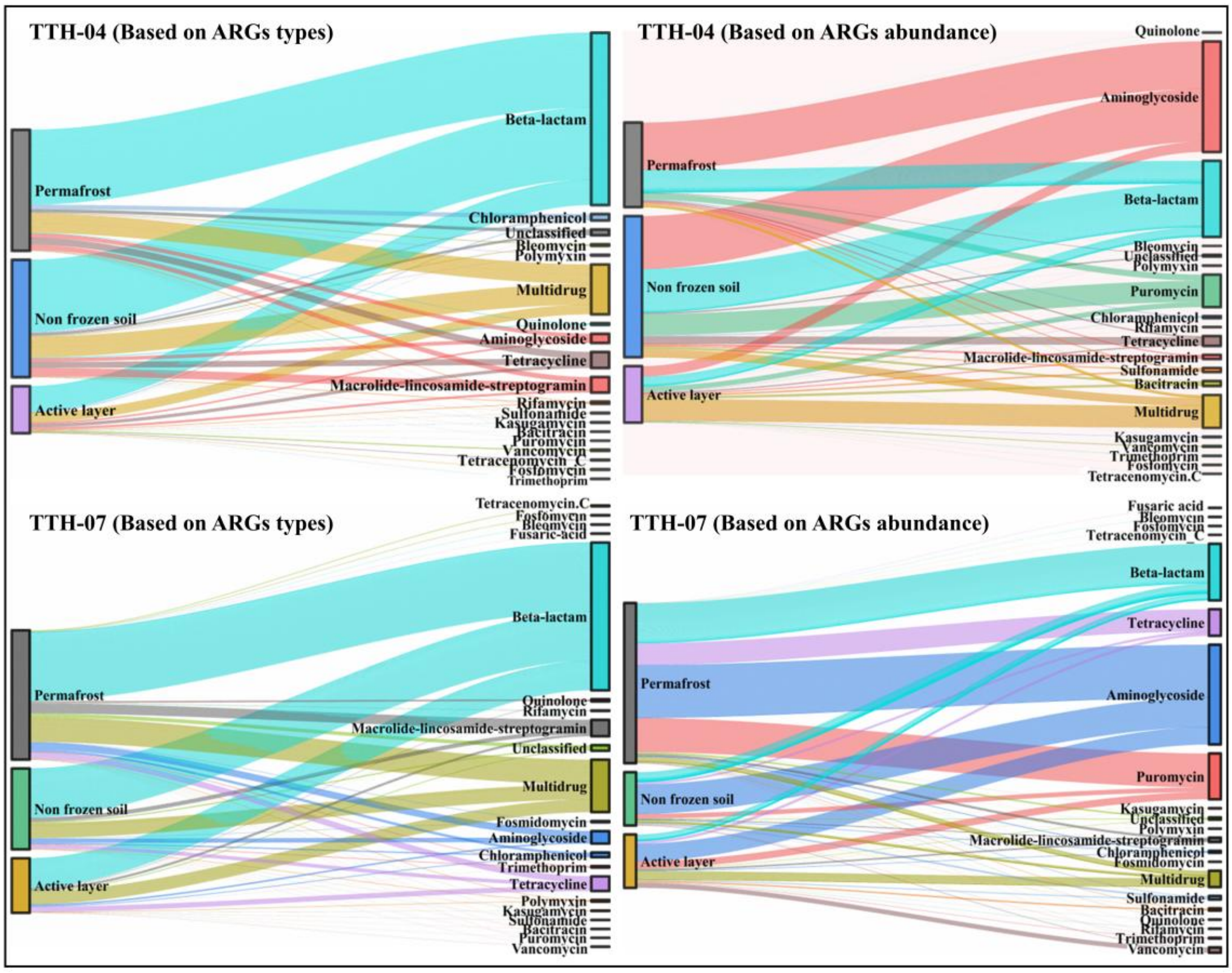

Supplementary Fig. 4. Sankey Diagram of ARG Diversity and Abundance. Sankey analysis illustrating the diversity and abundance of ARGs across the active layer, permafrost, and non-frozen soil at sites TTH-04 and TTH-07. Colored strips represent different ARG classes, highlighting their distribution across the three soil layers.

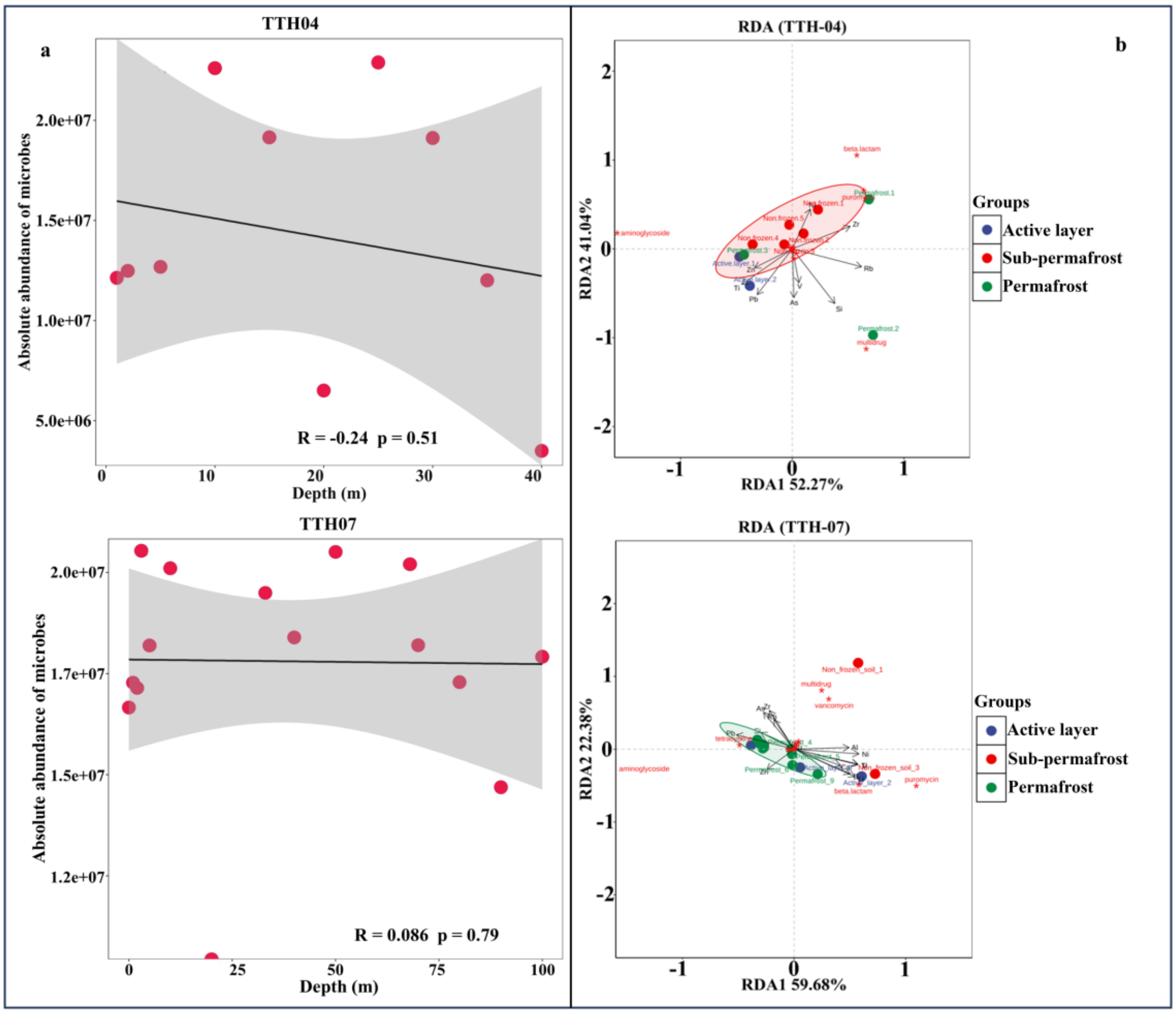

Supplementary Fig. 5. Distribution Patterns of Microbial Communities. (a) Spearman correlation analysis depicting the quantitative relationship between the absolute abundance of microbial communities and depth (m). P-values were adjusted for multiple testing using the Benjamini–Hochberg false discovery rate (FDR) correction, and significance is reported based on FDR-adjusted values. (b) Redundancy analysis (RDA) assessing the relationship between active ARG communities and metal ions across permafrost layers.

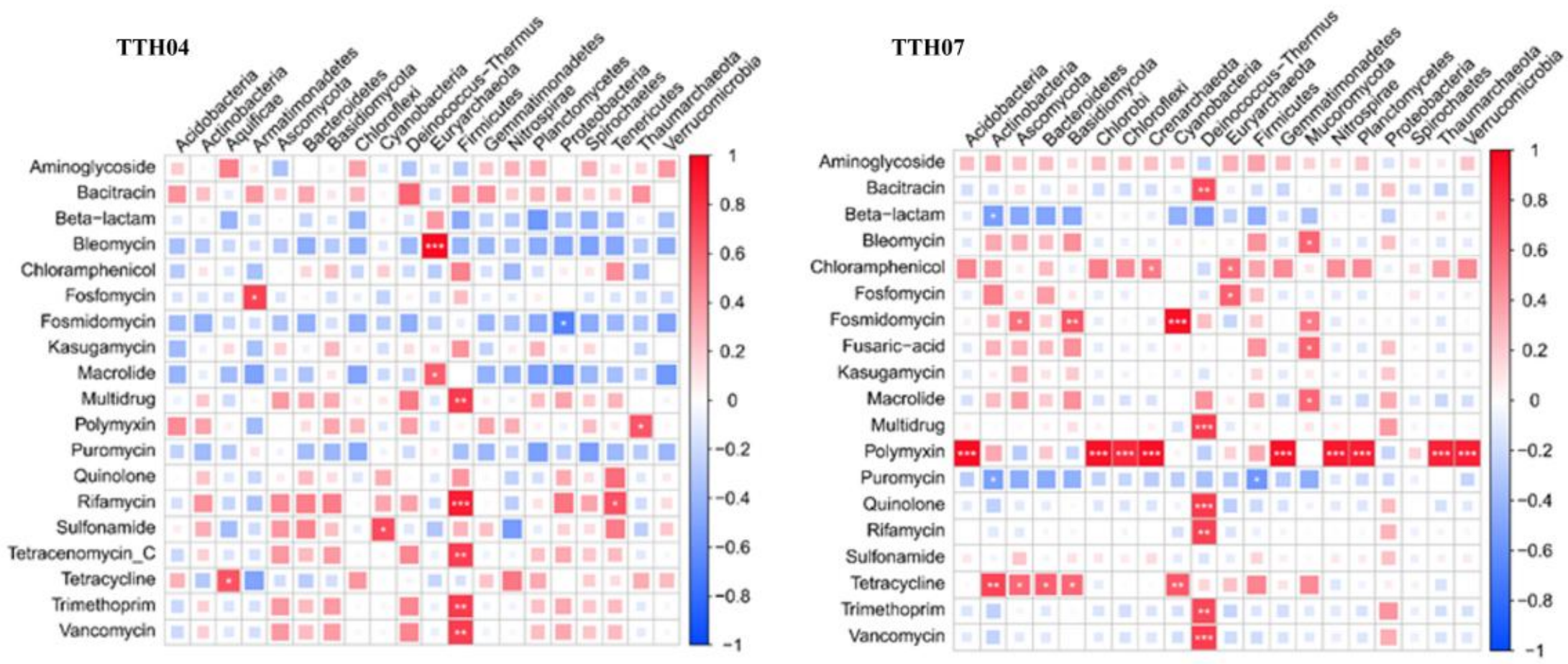

Supplementary Fig. 6. Correlation of ARG Families and Microbial Diversity. Spearman correlation analysis depicting the quantitative relationship between the absolute abundance of microbial communities (top 20 phyla) and ARG families. Asterisks denote statistical significance (****P* <0.001; ***P* <0.01; **P* <0.05).

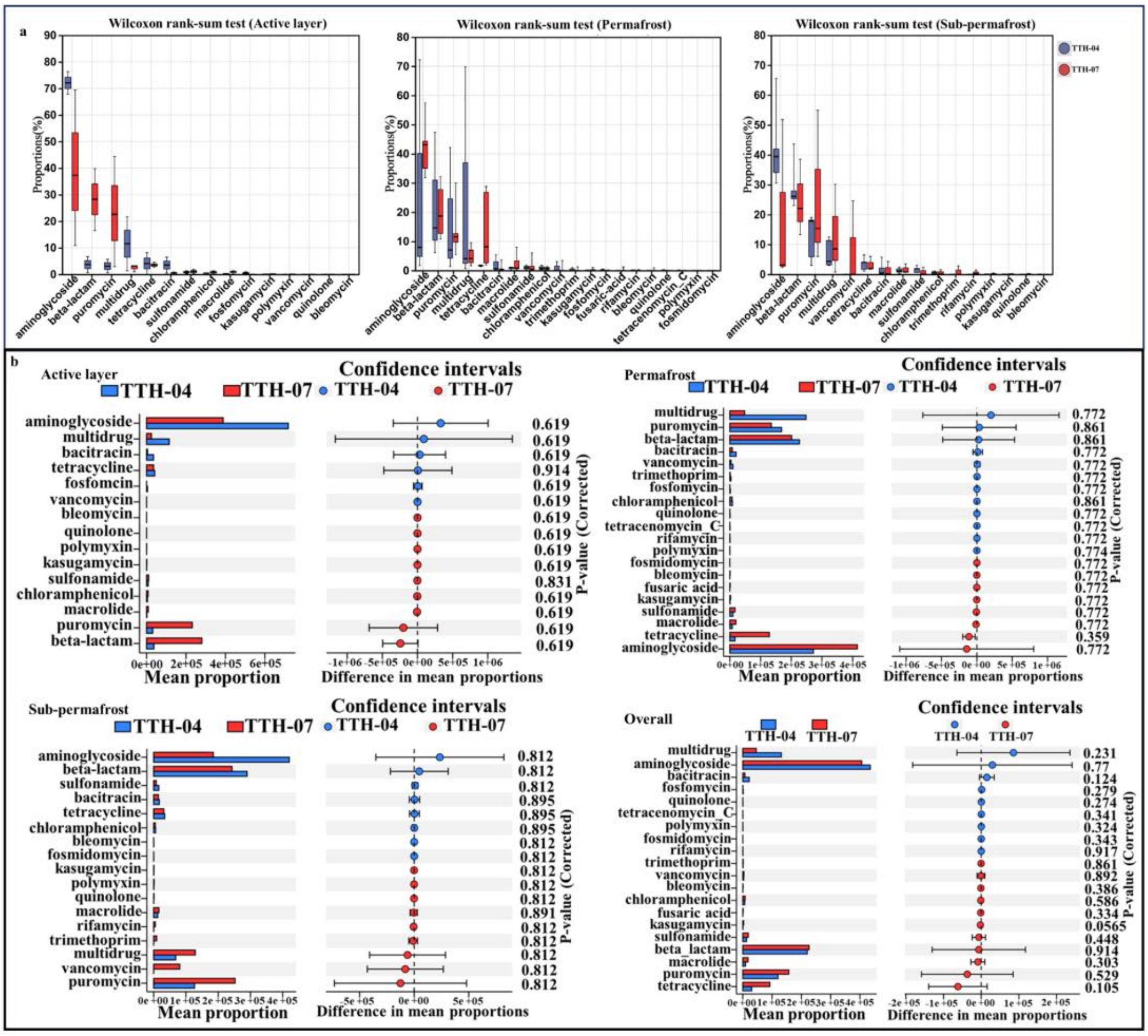

Supplementary Fig. 7. Differences in ARG Classes Between Sites TTH-04 and TTH-07. (a) Statistical differences in ARG classes between the permafrost layers of two sites (TTH-04 and TTH-07) and overall, the two sites were assessed using the Wilcoxon rank-sum test. (b) Statistical differences in ARG classes were analyzed using STAMP. All *P*-values were adjusted for multiple comparisons using the Benjamini–Hochberg procedure.

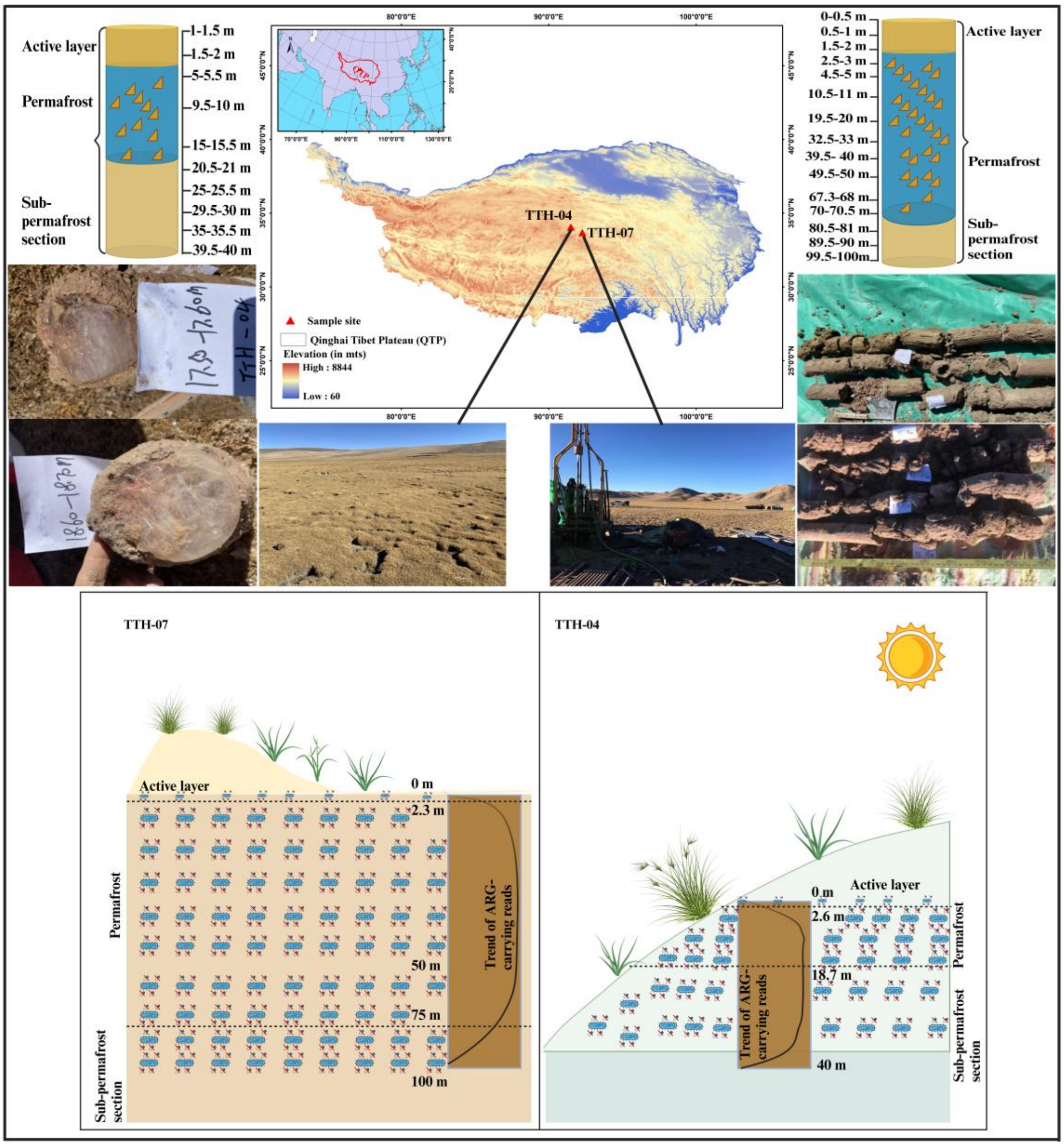

Supplementary Fig. 8. Map of The Study Area in The Tuotuo River Permafrost Region, the Headwater of The Yangtze River. Red markers indicate the sampling sites, with accompanying field photographs showing the sampling locations and extracted permafrost cores. Conceptual diagrams illustrate site characteristics, drilling depths, and the stratification of active, permafrost, and sub-permafrost layers. Drilling was conducted at elevations ranging from 4,850 to 5,005 m a.s.l., yielding 100 m (TTH-07) and 40 m (TTH-04) permafrost cores (sections shown in photographs). Core sampling included ten depth intervals from TTH-04 and fifteen depth intervals from TTH-07 (see Supplementary Table 4 for detailed sample metadata). Field photographs are presented for illustrative purposes and do not represent laboratory subsampling conditions. The study area map was generated by the authors using ArcGIS 10.8 (ESRI) based on original sampling coordinates. Background topographic data were derived from the

NASA Global DEM Version 2 (30 m resolution), and country boundary and Qinghai–Tibet Plateau shapefiles were obtained from publicly available IGIS Map datasets.

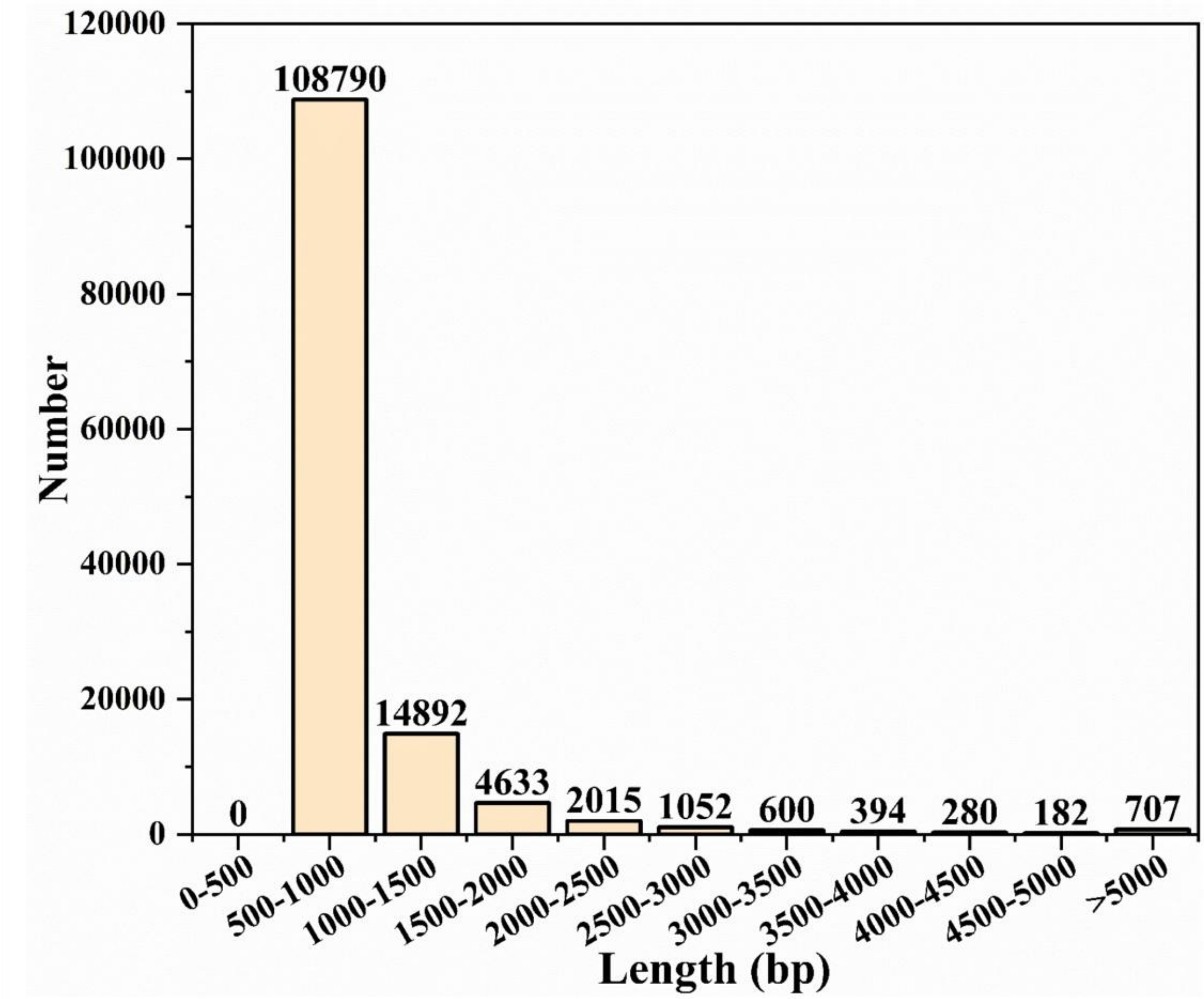

Supplementary Fig. 9. Distribution of Assembled Contig Lengths. The X-axis Represents Contig Length Intervals, While the Y-axis Indicates the Number of Sequences Within Each Interval. No contigs were observed in the 0–500 bp range. The highest number of contigs fell within the 500–1000 bp interval, followed by the 1000–1500 bp range. A total of 707 contigs exceeded 5000 bp in length.

Supplementary Table 1. Summary of Meta-transcriptomic Sequencing Data for Permafrost Cores Samples From TTH-04 and TTH-07. This provides detailed sequencing information for core samples collected from sites TTH-04 (TTH04-1 to TTH04-40) and TTH-07 (TTH07-0 to TTH07-100) in the Tuotuo River permafrost region, Qinghai-Tibet Plateau, China. The table includes metrics such as raw read counts, clean read counts, quality control parameters, and GC content for each sample. Sequencing quality and data processing were conducted by Wuhan Benagen Technology Company Limited (BENAGEN).

Sample	Raw reads	Raw bases	Clean reads	Clean bases	ARGs-carrying reads number (PE)	Q20_rate	Q30_rate	GC content
**TTH04-1**	62,945,684	944,185,260,0	62,167,772	60,024,768,20	730	98.46%	95.41%	56.37%
**TTH04-2**	84,480,502	126,720,753,00	84,225,738	57,410,501,15	1,181	98.79%	96.33%	61.89%
**TTH04-5**	72,757,456	10,913,618,400	65,372,054	5,978,811,905	16,369	98.09%	95.09%	56.37%
**TTH04-10**	81,586,370	12,237,955,500	80,342,030	8,990,007,729	13,900	98.29%	94.94%	54.36%
**TTH04-15**	60,520,458	90,780,687,00	54,573,392	47,188,781,33	2,443	98.37%	95.27%	56.25%
**TTH04-20**	86,413,208	12,961,981,200	31,553,214	2,116,003,769	1,428	93.62%	87.58%	56.18%
**TTH04-25**	55,112,626	82,668,939,00	54,735,002	53,171,080,71	1,379	98.38%	95.11%	55.81%
**TTH04-30**	70,006,276	105,009,414,00	60,214,546	68,357,736,20	8,474	98.40%	95.36%	55.08%
**TTH04-35**	77,055,274	115,582,911,00	76,713,706	90,353,056,96	1,482	98.54%	95.42%	55.32%
**TTH04-40**	73,841,488	110,762,232,00	32,094,464	31,013,440,15	1,651	97.06%	93.18%	46.38%
**TTH07-0**	73,589,064	110,383,596,00	73,276,368	51,055,242,81	285	98.71%	96.11%	63.11%
**TTH07-1**	78,079,568	117,119,352,00	77,574,594	52,190,211,02	938	98.48%	95.86%	63.79%
**TTH07-2**	61,322,768	91,984,152,00	61,002,348	67,119,554,76	2,216	98.60%	95.78%	56.42%
**TTH07-3**	74,687,556	112,031,334,00	74,046,656	72,111,920,27	34,195	98.61%	95.95%	56.34%
**TTH07-5**	73,551,308	110,326,962,00	72,881,238	70,482,829,41	7,040	98.75%	96.19%	56.34%
**TTH07-10**	64,560,552	149,990,262,00	64,173,230	56,263,291,50	13,134	98.77%	96.27%	57.46%
**TTH07-20**	99,993,508	118,408,821,00	99,047,244	101,410,693,47	59,061	98.51%	95.38%	53.62%

**TTH07-33**	78,939,214	100,520,964,00	75,797,264	66,411,848,27	45,605	98.69%	96.06%	55.70%
**TTH07-40**	67,013,976	11,541,527,100	66,487,856	66,042,505,35	19,750	98.90%	96.39%	54.71%
**TTH07-50**	76,943,514	103,461,894,00	76,515,806	6,857,193,751	7,438	55.46%	96.31%	55.46%
**TTH07-68**	68,974,596	103,461,894,00	68,369,352	63,191,836,33	12,174	56.71%	96.31%	56.71%
**TTH07-70**	59,313,590	88,970,385,00	54,982,230	55,004,544,22	9,526	51.20%	94.34%	51.20%
**TTH07-80**	68,533,624	102,800,436,00	66,511,076	61,526,154,01	6,141	98.45%	95.68%	56.01%
**TTH07-90**	60,738,714	91,108,071,00	60,357,850	60,123,198,61	316	98.27%	94.97%	58.28%
**TTH07-100**	68,681,080	10,302,162,000	54,631,074	4,814,977,953	21,654	97.21%	93.28%	54.45%

Legend Raw reads are the number of original sequences; Raw base is the amount of raw data; Clean reads are the number of filtered sequences; Clean base is the amount of data after filtering; ARGs-carrying reads number are the number of paired reads compared to the ARG; Q20 is the Q20 reads ratio after filtering; Q30 is the filtered Q30 reads ratio; GC is the GC ratio of filtered reads.

Supplementary Table 2. Exact correlation coefficients and adjusted P-values for multiple testing in the Mantel test using the Benjamini–Hochberg false discovery rate (FDR) correction, and significance is reported based on FDR-adjusted values. Reported P-values are rounded to three decimal places.

**TTH04**				
**spec**	**env**	**r**	**p.value**	**p.adj.FDR**
ARGs	Ti	0.88946	0.0001	**0.0102**
MTGs	Rb	0.8565	0.0004	**0.0204**
ARGs	Nb	0.81489	0.0006	**0.0204**
MGEs	K	0.67791	0.0008	**0.0204**
MTGs	Li	0.64413	0.0009	**0.0184**
MGEs	Ph	0.45936	0.013	0.221
ARGs	TN	0.72635	0.015	0.2295
ARGs	B	0.78379	0.015	0.2295
MTGs	K	0.37285	0.032	0.4352
MGEs	Hf	0.39936	0.042	0.476
MGEs	Fe	0.39939	0.05	0.51
MGEs	Ca	0.39939	0.05	0.51
MGEs	Zr	0.37067	0.057	0.528
MGEs	Na	0.29079	0.079	0.6715
MGEs	Ho	0.27137	0.084	0.672
MTGs	Si	0.30055	0.098	0.714
ARGs	Cs	0.32379	0.101	0.714
MGEs	Cs	0.25646	0.11	0.714
MGEs	Dy	0.24633	0.12	0.72
MGEs	As	0.22277	0.123	0.72
MGEs	Er	0.23048	0.131	0.726
MGEs	V	0.1875	0.14	0.7344
MGEs	Ga	0.26843	0.141	0.7344
MTGs	Pb	0.17914	0.144	0.7344
MGEs	Pb	0.14978	0.165	0.748
ARGs	Lu	0.20771	0.173	0.748
MGEs	Tb	0.22706	0.185	0.7548
MTGs	As	0.15501	0.187	0.7548
MGEs	Yb	0.17837	0.188	0.7548
MGEs	Gd	0.22335	0.191	0.7548
MTGs	Cs	0.16373	0.211	0.7914
MGEs	Zn	0.14299	0.223	0.7998
MGEs	Rb	0.1504	0.223	0.7998
MGEs	Eu	0.19771	0.224	0.7998

MGEs	TN	0.17439	0.235	0.7998
MGEs	Tm	0.11961	0.237	0.7998
ARGs	Tm	0.11036	0.243	0.7998
MGEs	Lu	0.13345	0.251	0.7998
MTGs	V	0.09328	0.254	0.7998
MGEs	Li	0.10248	0.264	0.7998
ARGs	Pb	0.0788	0.267	0.7998
ARGs	Cu	0.1466	0.271	0.7998
MTGs	Hf	0.0903	0.287	0.7998
ARGs	Yb	0.04505	0.306	0.7998
MTGs	Zr	0.0821	0.308	0.7998
MGEs	Ni	0.0806	0.327	0.7998
MTGs	Ph	0.04288	0.332	0.7998
ARGs	Bi	0.05621	0.346	0.7998
MGEs	Mg	0.04898	0.356	0.7998
MTGs	Na	0.03589	0.359	0.7998
ARGs	Er	0.00517	0.393	0.7998
MTGs	Ca	0.02096	0.395	0.7998
MGEs	B	-0.026	0.407	0.7998
ARGs	Al	-0.0052	0.416	0.7998
MTGs	Fe	0.02096	0.416	0.7998
MTGs	Cu	0.01159	0.457	0.7998
MGEs	Al	-0.0098	0.458	0.7998
ARGs	As	-0.0664	0.471	0.7998
ARGs	Ho	-0.0637	0.519	0.7998
MTGs	Yb	-0.0359	0.525	0.7998
MTGs	Ga	-0.0788	0.545	0.7998
ARGs	Ph	-0.0605	0.566	0.7998
MTGs	Ni	-0.0693	0.576	0.7998
MTGs	Eu	-0.094	0.587	0.7998
MGEs	Ti	-0.0832	0.622	0.7998
MTGs	Zn	-0.0965	0.623	0.7998
MGEs	Nb	-0.1013	0.625	0.7998
MGEs	Bi	-0.0616	0.626	0.7998
MTGs	Tm	-0.0799	0.627	0.7998
ARGs	Li	-0.1257	0.63	0.7998
ARGs	Rb	-0.1424	0.632	0.7998
MTGs	Er	-0.0857	0.634	0.7998
MTGs	Ti	-0.0832	0.635	0.7998
ARGs	Zn	-0.1512	0.672	0.7998
MGEs	Si	-0.1394	0.714	0.7998

ARGs	Fe	-0.1798	0.733	0.7998
MTGs	Nb	-0.1498	0.733	0.7998
MTGs	Tb	-0.1678	0.737	0.7998
MTGs	Dy	-0.1591	0.741	0.7998
MTGs	Ho	-0.1414	0.741	0.7998
ARGs	Ca	-0.1798	0.746	0.7998
MTGs	Al	-0.1212	0.756	0.7998
MTGs	Lu	-0.137	0.763	0.7998
MTGs	B	-0.1735	0.766	0.7998
MTGs	Gd	-0.1803	0.772	0.7998
ARGs	Ni	-0.2004	0.783	0.7998
ARGs	Dy	-0.2043	0.8	0.7998
ARGs	Si	-0.2219	0.814	0.7998
MTGs	TN	-0.2238	0.823	0.7998
MTGs	Bi	-0.1475	0.85	0.7998
MTGs	Mg	-0.2026	0.884	0.7998
ARGs	K	-0.2169	0.892	0.7998
ARGs	Hf	-0.2399	0.943	0.7998
ARGs	V	-0.2401	0.945	0.7998
ARGs	Zr	-0.2329	0.951	0.7998
ARGs	Mg	-0.2308	0.956	0.7998
ARGs	Eu	-0.2666	0.964	0.7998
ARGs	Na	-0.2657	0.979	0.7998
ARGs	Tb	-0.2847	0.981	0.7998
ARGs	Ga	-0.2607	0.984	0.7998
ARGs	Gd	-0.2799	0.991	0.7998
MGEs	Cu	-0.2898	0.994	0.7998
**TTH07**				
**spec**	**env**	**r**	**raw_p**	**p.adj.FDR**
**ARGs**	Eu	0.704	0.0001	**0.0102**
**MGEs**	Eu	0.739	0.0003	**0.0153**
**MGEs**	Bi	0.324	0.006	0.306
**ARGs**	Zr	0.308	0.01	0.34
**ARGs**	Hf	0.339	0.011	0.34
**MGEs**	TN	0.293	0.011	0.34
**MTGs**	Bi	0.396	0.011	0.34
**MGEs**	Mg	0.293	0.016	0.408
**MGEs**	Hf	0.242	0.019	0.4307
**MGEs**	Zr	0.243	0.02	0.4307
**MGEs**	Fe	0.259	0.025	0.4636
**MGEs**	V	0.263	0.029	0.493

**MGEs**	Ca	0.251	0.03	0.493
**ARGs**	TN	0.212	0.031	0.493
**ARGs**	Gd	0.246	0.036	0.5324
**MGEs**	Li	0.217	0.046	0.5865
**MTGs**	Eu	0.255	0.049	0.5865
**MGEs**	Gd	0.179	0.058	0.658
**MTGs**	Gd	0.192	0.06	0.658
**ARGs**	Rb	0.321	0.076	0.7752
**MTGs**	B	0.296	0.078	0.7752
**MTGs**	V	0.286	0.087	0.8105
**MTGs**	Li	0.278	0.09	0.8105
**MTGs**	Na	0.177	0.094	0.8105
**MGEs**	B	0.172	0.099	0.8105
**ARGs**	Si	0.248	0.108	0.8424
**MGEs**	Sn	0.188	0.109	0.8424
**ARGs**	Zn	0.155	0.115	0.8424
**MTGs**	TN	0.119	0.119	0.8424
**MTGs**	Pb	0.215	0.128	0.8694
**MTGs**	Tb	0.217	0.149	0.9229
**MGEs**	Rb	0.119	0.17	0.982
**MGEs**	Pb	0.144	0.171	0.982
**ARGs**	Bi	0.112	0.187	0.982
**MTGs**	Cu	0.153	0.193	0.982
**MGEs**	K	0.144	0.203	0.982
**MTGs**	Ti	0.231	0.203	0.982
**MGEs**	Cs	0.067	0.216	0.982
**MTGs**	Lu	0.205	0.218	0.982
**ARGs**	V	0.084	0.22	0.982
**MTGs**	Rb	0.124	0.228	0.982
**MTGs**	Yb	0.202	0.231	0.982
**MTGs**	Tm	0.186	0.246	0.982
**ARGs**	B	0.065	0.25	0.982
**MTGs**	Mg	0.074	0.252	0.982
**MTGs**	Ni	0.087	0.252	0.982
**MTGs**	Dy	0.168	0.276	0.982
**MTGs**	As	0.162	0.281	0.982
**MTGs**	Er	0.167	0.284	0.982
**MTGs**	Ga	0.119	0.287	0.982
**MTGs**	Ho	0.147	0.3	0.982
**MGEs**	Ga	0.065	0.307	0.982
**MGEs**	Si	0.083	0.308	0.982

**ARGs**	Li	0.026	0.309	0.982
**MTGs**	Nb	0.094	0.332	0.982
**MGEs**	Ti	0.022	0.377	0.982
**MGEs**	Yb	0.012	0.411	0.982
**MTGs**	Sn	0.043	0.413	0.982
**ARGs**	Sn	-0.02	0.428	0.982
**MGEs**	Tb	0.023	0.431	0.982
**MGEs**	Ni	0.01	0.432	0.982
**MTGs**	Cs	0.006	0.435	0.982
**MTGs**	Zr	0.012	0.437	0.982
**MGEs**	Lu	0.01	0.452	0.982
**MGEs**	As	0.014	0.453	0.982
**MTGs**	Si	-0.012	0.471	0.982
**MGEs**	Na	-0.01	0.496	0.982
**MTGs**	Hf	-0.004	0.5	0.982
**ARGs**	Ni	-0.02	0.506	0.982
**ARGs**	Mg	-0.022	0.51	0.982
**MGEs**	Tm	-0.021	0.519	0.982
**MGEs**	Nb	-0.041	0.531	0.982
**MGEs**	Er	-0.055	0.556	0.982
**MGEs**	Dy	-0.065	0.587	0.982
**MGEs**	Ho	-0.076	0.596	0.982
**MTGs**	Ca	-0.078	0.61	0.982
**MTGs**	Fe	-0.078	0.611	0.982
**ARGs**	Cu	-0.085	0.661	0.982
**ARGs**	Al	-0.065	0.672	0.982
**ARGs**	Lu	-0.106	0.681	0.982
**MGEs**	Cu	-0.062	0.688	0.982
**MGEs**	Al	-0.071	0.705	0.982
**MTGs**	K	-0.16	0.714	0.982
**ARGs**	Pb	-0.114	0.73	0.982
**ARGs**	Ga	-0.143	0.75	0.982
**ARGs**	Cs	-0.071	0.755	0.982
**MTGs**	Al	-0.092	0.769	0.982
**ARGs**	Yb	-0.158	0.77	0.982
**MGEs**	Zn	-0.081	0.789	0.982
**ARGs**	As	-0.171	0.797	0.982
**ARGs**	Tm	-0.172	0.799	0.982
**ARGs**	Ca	-0.158	0.811	0.982
**ARGs**	Tb	-0.159	0.811	0.982
**ARGs**	Fe	-0.157	0.842	0.982

**ARGs**	Er	-0.219	0.892	0.982
**ARGs**	Ph	-0.213	0.893	0.982
**ARGs**	Ti	-0.232	0.908	0.982
**ARGs**	Nb	-0.226	0.909	0.982
**ARGs**	Dy	-0.243	0.936	0.982
**ARGs**	Na	-0.214	0.944	0.982
**ARGs**	Ho	-0.248	0.946	0.982
**MTGs**	Zn	-0.172	0.947	0.982
**MGEs**	Ph	-0.235	0.987	0.982
**ARGs**	K	-0.273	0.994	0.982
**MTGs**	Ph	-0.345	0.997	0.982

Supplementary Table 3. Summary of Network Analysis for TTH-04 and TTH-07. Key topological parameters of the co-occurrence network analysis for samples from sites TTH-04 and TTH-07, including node count, edge density, average degree, clustering coefficient, and modularity. These metrics provide insights into the structural complexity and connectivity of ARGs, MTGs, and microbial taxa within each network.

S. No	Network Metrics/Top Phyla	Values	Top Phyla
		**TTH-04**	
**1**	Nodes	384	Chloroflexi (Hub = 0.294), 29 edges (ARGs)
**2**	Edges	1255	Ascomycota (Hub = 0.284), 28 edges (ARGs)
**3**	Positive	1004	Basidiomycota (Hub = 0.274), 25 edges (ARGs)
**4**	Negative	251	C. Wolfebacteria (Hub = 0.256), 24 edges (ARGs)
**5**	Graph Density	0.009	Tenericutes (Hub = 0.254), 21 edges (ARGs)
**6**	Modularity	0.548	….
**7**	Statistical Interference	5725.35	….
		**TTH-07**	
**8**	Nodes	362	C. Bipolaricaulota (Hub = 0.528), 97 edges (ARGs = 93; MTGs = 4)
**9**	Edges	1113	C. Geotheramarchaeota (Hub = 0.502), 87 edges (ARGs = 86; MTGs = 1)
**10**	Positive	395	Balneolaeota (Hub = 0.387), 65 edges (ARGs = 64; MTGs = 1)
**11**	Negative	718	Chlorbi (Hub = 0.326), 54 edges (ARGs = 52; MTGs = 2)
**12**	Graph Density	0.009	Calditrichaeota (Hub = 0.259), 43 edges (ARGs = 41; MTGs = 2)
**13**	Modularity	0.514	….
**14**	Statistical Interference	5002.88	….

Supplementary Table 4. Detailed Metadata of Permafrost Core Samples Collected from Sites TTH-04 and TTH-07 in the Tuotuo River Permafrost Region, Qinghai-Tibet Plateau, China. The table includes information on section profiles, sampling depths, ground temperature and lithological characteristics of each collected sample. These features were meticulously documented during intensive field observations to ensure accuracy and representativeness.

Sample	Depth (m)	GT (˚C)	Profile	Lithological characteristics	
**TTH04-1**	1-1.5	0.75	Active Layer	0-8.2 m (taupe)	Around 2.6 m was the upper limit of the active layer. Depth 0-20 cm was gray-brown and contained a surface organic matter layer; ice crystals were visible, and the overall structure contained a large amount of gravel (<10 mm). Depth 1.1-1.35 m was rock (soil content was about 50%). Depth 2-2.15 m was loose core. Depth 2.4-6.0 m contained layered ice, layered structures were developed, and the core was dense and compact. Depth 6.0-8.2 m developed layered ice (1 mm), complete core, silty sandy loam (sticky).
**TTH04-2**	1.5-2	0.70	Active Layer		
**TTH04-5**	5-5.5	-0.75	Permafrost		
**TTH04-10**	9.5-10	-0.60	Permafrost	8.2-9.5 m (tan)	A small amount of vein ice and layered ice can be seen with the naked eye, with a thickness of 1mm. The overall structure was complete, with a small amount of breccia.
**TTH04-15**	15-15.5	-0.40	Permafrost	9.5-40 m (off-white)	Sandstone, complete core, severely weathered. At a depth of 10.2-10.3 m, layered ice was seen (thickness of 2 mm), and ice inclusions were developed. Depth 10.3-27.1 m was quartz sandstone with a complete core and severe weathering. At depth 17.5 -17.6 m, a complete layer of ice was seen, with a thickness of <2 cm, a layered structure and a complete core. At depth 17.6-18.6 m, reddish-brown oxide cement was common inside the core. Depth 18.6-18.7 m, layered ice was seen (<2 cm), a layered structure and a complete core. At about 25m, groundwater was seen in the hole. At 27.1-41.3 m sandstone, reddish-brown cementation was common in the core, severely weathered and the texture became soft, the core was loose, and the sandstone particle size became coarser and <5 mm.
**TTH04-20**	20.5-21	NA	Sub-permafrost section		
**TTH04-25**	25-25.5	NA	Sub-permafrost section		
**TTH04-30**	29.5-30	NA	Sub-permafrost section		
**TTH04-35**	35-35.5	NA	Sub-permafrost section		
**TTH04-40**	39.5-40	NA	Sub-permafrost section		
**TTH07-0**	0-0.5	2.5	Active Layer	0-36.05 m (reddish brown)	Depth 0-0.1 m contained an organic matter layer and broken core; 0.1-1.5 m, loose core; depth 1.5-2.3 m, completely broken with a small amount of gravel; 2.3-2.6 m, a large amount of visible soil-containing reticulated ice layer, in the form of ice inclusions, inferred to be on permafrost. At 2.6-2.7 m, layered ice was visible (5 cm), with visible gravel. At 2.7-3.3 m, the core was broken with gravel, and ice lenses were reported at 3.0-3.3 m. At 3.3-4.5 m, the complete core and ice were visible in a network structure and contained a large number of ice inclusions and pure ice layers (3 cm). At 4.5-5.3 m, containing gravel and visible fissure ice; at 5.3-12.0 m, a complete core containing soil and ice layers, and ice inclusions were clear, with gravel (about 5-10%); at 12.0-36.05 m, a relatively complete core, and visible ice, belongs to repeated segregation ice. Layered ice, vein-like ice, and ice inclusions were seen, with gravel (about 10-15%). At 12.6-12.8 m, layered ice (about 2 cm) was visible; at 17.5-17.6 m, vein-
**TTH07-1**	0.5-1	2.4	Active Layer		
**TTH07-2**	1.5-2	1.8	Active Layer		
**TTH07-3**	2.5-3	-0.42	Permafrost		
**TTH07-5**	4.5-5.0	-1.4	Permafrost		
**TTH07-10**	10.5-11	-2.4	Permafrost		
**TTH07-20**	19.5-20	-2.37	Permafrost		

**TTH07-33**	32.5-33	NA	Permafrost		like ice was visible; at 22.8-22.9 m, layered ice (about 2cm) was visible; and at 24.2-24.3 m, ice inclusions were visible.
**TTH07-40**	39.5-40	NA	Permafrost	36.3-74 m (reddish brown)	At 37-41.4 m, complete core, vein-like and layered ice, and visible ice inclusions, containing gravel (5%); at 37.0-37.6 m, vein-like ice and ice inclusions were visible; at 37.6-37.7 m, pure ice layer (8 cm) was present; 40.4-40.9 m, pure ice core (0.5 m long) was reported; 41.3-41.4 m, ice inclusions were visible; 46-47.5 m, broken core, invisible ice, contained gravel (5%); 47.5-56.4 m, complete core, fine ice crystals. Ice inclusions were seen at 49.8-49.7 m. Broken core at 51.0-52.1 m; at 52.1-52.2 m, vein-like ice was present; at 56.4-57 m, broken core with no ice; at 57.0-58.2 m, complete core with no ice; at 58.2-59.5 m, fine ice crystals; at 59.5-60.0 m, broken core with no ice; at 60.0-61.0 m, complete core with no ice; at 61.0-63.0 m, broken core with some gravel and breccia; at 63.0-69.0 m, broken core containing some gravel (breccia, boulder), and groundwater appears at 67-68 m; at 69.0-69.2 m, complete core with small ice crystals; at 72-74 m, complete core, with small amount of gravel and breccia.
**TTH07-50**	49.5-50	NA	Permafrost		
**TTH07-68**	67.3-68	NA	Permafrost		
**TTH07-70**	70-70.5	NA	Permafrost		
**TTH07-80**	80.5-81	NA	Sub-permafrost section	74-91 m (earthy yellow)	At 74-83 m, no ice with some gravel and breccia; 74.6-76.4 m, broken core; 76.4-80.6 m, complete core; 80.6-83 m, broken core broken; 83-85 m, coarse sandy soil with no ice; at 83-83.5 m, core was broken; at 83.5-83.6 m, complete core with sandstone appears; at 83.6-85 m, core was broken; at 85-91 m, fine sandy soil with no ice.
**TTH07-90**	89.5-90	NA	Sub-permafrost section		
**TTH07-100**	99.5-100	NA	Sub-permafrost section	91-100 m (purple-red)	Fine sandy soil with no ice. At 91-92 m, broken core; at 92-92.6 m, complete core; at 92.6-93 m, broken core; 93-93.5 m, reddish-brown soil with no ice, 93.5-100 m, light loam, purple-red, complete core, no ice, containing 5-10% gravel, with gravel particle sizes ranging from 0.5-5 cm.
**Legend**					NA = Not available

Supplementary Table 5. RNA quality control metrics for permafrost samples from two cores (TTH04; TTH07). RNA concentrations and library preparation success varied with depth and stratigraphic zone. Only samples exceeding low-input library requirements were sequenced. Negative controls yielded no detectable nucleic acids.

Samples	Permafrost core	Depth (m)	Stratigraphic zone	Extraction method	RNA conc. (ng μL⁻¹, mean±SD, Qubit RNA HS, n=3)	DNA conc. (ng μL⁻¹, mean±SD, Qubit DNA HS, n=3)	Total RNA yield (ng, mean±SD)	A260/280 (NanoDrop, mean±SD)	DNase treatment	Library prepared	Notes
**TTH04-1**	TTH04	1-1.5	Active Layer	TRIzol + column cleanup	7.32±0.12	< BDL	146.4 ± 2.4	1.92 ± 0.05	Yes	Yes	Best library quality
**TTH04-2**	TTH04	1.5-2	Active Layer	TRIzol + column cleanup	6.90±0.16	< BDL	138.0 ± 3.2	1.91 ± 0.04	Yes	Yes	Best library quality
**TTH04-5**	TTH04	5-5.5	Permafrost	TRIzol + column cleanup	5.85±0.12	< BDL	117.0 ± 2.4	1.89 ± 0.03	Yes	Yes	Best library quality
**TTH04-10**	TTH04	9.5-10	Permafrost	TRIzol + column cleanup	6.32±0.21	< BDL	126.4 ± 4.2	1.90 ± 0.06	Yes	Yes	Best library quality

Samples	Permafrost core	Depth (m)	Stratigraphic zone	Extraction method	RNA conc. (ng μL⁻¹, mean±SD, Qubit RNA HS, n=3)	DNA conc. (ng μL⁻¹, mean±SD, Qubit DNA HS, n=3)	Total RNA yield (ng, mean±SD)	A260/280 (NanoDrop, mean±SD)	DNase treatment	Library prepared	Notes
**TTH04-15**	TTH04	15-15.5	Permafrost	TRIzol + column cleanup	6.12±0.21	< BDL	122.4 ± 4.2	1.88 ± 0.05	Yes	Yes	Best library quality
**TTH04-20**	TTH04	20.5-21	Sub-permafrost section	TRIzol + column cleanup	7.22±0.43	< BDL	144.4 ± 8.6	1.93 ± 0.08	Yes	Yes	Best library quality
**TTH04-25**	TTH04	25-25.5	Sub-permafrost section	TRIzol + column cleanup	5.83±0.26	< BDL	116.6 ± 5.2	1.87 ± 0.07	Yes	Yes	Best library quality
**TTH04-30**	TTH04	29.5-30	Sub-permafrost section	TRIzol + column cleanup	6.28±0.33	< BDL	125.6 ± 6.6	1.90 ± 0.05	Yes	Yes	Best library quality
**TTH04-35**	TTH04	35-35.5	Sub-permafrost section	TRIzol + column cleanup	7.66±0.67	< BDL	153.2 ± 13.4	1.94 ± 0.09	Yes	Yes	Best library quality

Samples	Permafrost core	Depth (m)	Stratigraphic zone	Extraction method	RNA conc. (ng µL⁻¹, mean±SD, Qubit RNA HS, n=3)	DNA conc. (ng µL⁻¹, mean±SD, Qubit DNA HS, n=3)	Total RNA yield (ng, mean±SD)	A260/280 (NanoDrop, mean±SD)	DNase treatment	Library prepared	Notes
**TTH04-40**	TTH04	39.5-40	Sub-permafrost section	TRIzol + column cleanup	6.22±0.13	< BDL	124.4 ± 2.6	1.90 ± 0.03	Yes	Yes	Best library quality
**TTH07-0**	TTH07	0-0.5	Active Layer	TRIzol + column cleanup	6.66±0.19	< BDL	133.2 ± 3.8	1.91 ± 0.04	Yes	Yes	Best library quality
**TTH07-1**	TTH07	0.5-1	Active Layer	TRIzol + column cleanup	7.0±0.14	< BDL	140.0 ± 2.8	1.92 ± 0.05	Yes	Yes	Best library quality
**TTH07-2**	TTH07	1.5-2	Active Layer	TRIzol + column cleanup	6.28±0.22	< BDL	125.6 ± 4.4	1.90 ± 0.04	Yes	Yes	Best library quality
**TTH07-3**	TTH07	2.5-3	Permafrost	TRIzol + column cleanup	5.55±0.34	< BDL	111.0 ± 6.8	1.88 ± 0.06	Yes	Yes	Best library quality

**Samples**	**Permafrost core**	**Depth (m)**	**Stratigraphic zone**	**Extraction method**	**RNA conc. (ng µL⁻¹, mean±SD, Qubit RNA HS, n=3)**	**DNA conc. (ng µL⁻¹, mean±SD, Qubit DNA HS, n=3)**	**Total RNA yield (ng, mean±SD)**	**A260/280 (NanoDrop, mean±SD)**	**DNase treatment**	**Library prepared**	**Notes**
**TTH07-5**	TTH07	4.5-5.0	Permafrost	TRIzol + column cleanup	6.11±0.33	< BDL	122.2 ± 6.6	1.89 ± 0.05	Yes	Yes	Best library quality
**TTH07-10**	TTH07	10.5-11	Permafrost	TRIzol + column cleanup	4.27±0.03	< BDL	85.4 ± 0.6	1.86 ± 0.02	Yes	Yes	Successful library
**TTH07-20**	TTH07	19.5-20	Permafrost	TRIzol + column cleanup	6.92±0.43	< BDL	138.4 ± 8.6	1.92 ± 0.07	Yes	Yes	Best library quality
**TTH07-33**	TTH07	32.5-33	Permafrost	TRIzol + column cleanup	6.12±0.23	< BDL	122.4 ± 4.6	1.88 ± 0.04	Yes	Yes	Best library quality
**TTH07-40**	TTH07	39.5-40	Permafrost	TRIzol + column cleanup	4.12±0.12	< BDL	82.4 ± 2.4	1.85 ± 0.03	Yes	Yes	Successful library

Samples	Permafrost core	Depth (m)	Stratigraphic zone	Extraction method	RNA conc. (ng µL⁻¹, mean±SD, Qubit RNA HS, n=3)	DNA conc. (ng µL⁻¹, mean±SD, Qubit DNA HS, n=3)	Total RNA yield (ng, mean±SD)	A260/280 (NanoDrop, mean±SD)	DNase treatment	Library prepared	Notes
**TTH07-50**	TTH07	49.5-50	Permafrost	TRIzol + column cleanup	5.19±0.55	< BDL	103.8 ± 11.0	1.87 ± 0.08	Yes	Yes	Best library quality
**TTH07-68**	TTH07	67.3-68	Permafrost	TRIzol + column cleanup	6.92±0.58	< BDL	138.4 ± 11.6	1.91 ± 0.06	Yes	Yes	Best library quality
**TTH07-70**	TTH07	70-70.5	Permafrost	TRIzol + column cleanup	4.52±0.63	< BDL	90.4 ± 12.6	1.86 ± 0.09	Yes	Yes	Successful library
**TTH07-80**	TTH07	80.5-81	Sub-permafrost section	TRIzol + column cleanup	4.68±0.46	< BDL	93.6 ± 9.2	1.87 ± 0.07	Yes	Yes	Successful library
**TTH07-90**	TTH07	89.5-90	Sub-permafrost section	TRIzol + column cleanup	6.87±0.16	< BDL	137.4 ± 3.2	1.92 ± 0.04	Yes	Yes	Best library quality

Samples	Permafrost core	Depth (m)	Stratigraphic zone	Extraction method	RNA conc. (ng µL⁻¹, mean±SD, Qubit RNA HS, n=3)	DNA conc. (ng µL⁻¹, mean±SD, Qubit DNA HS, n=3)	Total RNA yield (ng, mean±SD)	A260/280 (NanoDrop, mean±SD)	DNase treatment	Library prepared	Notes
**TTH07-100**	TTH07	99.5-100	Sub-permafrost section	TRIzol + column cleanup	5.52±0.08	< BDL	110.4 ± 1.6	1.88 ± 0.02	Yes	Yes	Best library quality
**Extraction blank**	Process control	–	–	TRIzol + column cleanup	< BDL	< BDL	No measurable RNA was recovered	–	Yes	No	No detectable nucleic acids
**No-RT control**	Library prep control	–	–	Skip RT	–	< BDL	No cDNA amplification was detected	–	Yes	No	No amplification observed
**Legend**	No-RT controls (one per core) showed no detectable DNA contamination. BDL = Below detection limit.										

Supplementary Table 6. Assembly result data statistics.

Sample	Contig No	Total Length (bp)	Largest Len(bp)	N50(bp)	GC(%)
**TTH04-1**	798	588,969	2,664	705	51.64
**TTH04-2**	6,623	5,740,373	21,916	878	63
**TTH04-5**	1,137	1,026,082	28,400	778	53.87
**TTH04-10**	3,010	2,275,779	7,139	718	55.82
**TTH04-15**	2,624	1,915,728	5,299	692	58.29
**TTH04-20**	127	95,121	2,653	722	55.44
**TTH04-25**	4,016	2,996,603	6,956	719	60.08
**TTH04-30**	2,729	2,129,163	5,501	743	56.39
**TTH04-35**	2,643	1,917,923	6,918	688	56.37
**TTH04-40**	1,745	1,384,437	11,401	774	53.87
**TTH07-0**	2,109	1,531,754	8,223	688	57.99
**TTH07-1**	541	541,506	19,663	976	50.11
**TTH07-2**	3,123	2,423,718	7,359	741	56.52
**TTH07-3**	4,439	3,649,113	14,194	805	60.82
**TTH07-5**	8,691	9,244,900	15,135	1,144	61.06
**TTH07-10**	5,504	4,825,765	15,083	893	62.86
**TTH07-20**	1,559	1,320,952	14,194	773	53.25
**TTH07-33**	3,234	2,486,672	14,194	735	59.45
**TTH07-40**	6,013	4,739,709	64,631	748	59.95
**TTH07-50**	4,391	3,473,768	14,194	772	60.64
**TTH07-68**	2,738	2,191,601	14,194	734	58.1
**TTH07-70**	742	596,328	4,589	801	48.05
**TTH07-80**	8,260	6,800,774	143,291	793	56.59
**TTH07-90**	4,092	3,047,353	3,506	717	57.15
**TTH07-100**	260	182,308	3,027	654	50.86
**Legend**	Contig Num: The number of contigs after assembly; Total Len: the sum of the bases of all contigs; Largest Len: the number of bases of the longest contig; N50: The contigs are sorted from long to short and the cumulative length is counted, when a contig is added so that the cumulative length is equal to half of the sum of all contigs lengths, the length of the contig is N50; GC: G+C/A+T+G+C as a percentage.				

References

1. Guangyue, L.I.U. *et al*. Preliminary results of permafrost investigation on northern and southern slopes of Mt. Geladandong, interior Qinghai-Tibet Plateau. *J Glaciol Geocryol*. **44**, 83-95 (2022).
2. Zhibin, L.I. *et al*. Analysis of soil moisture content in the active layer in the permafrost region of the Tuotuo River source in frozen season. *J Glaciol Geocryol*. **44**, 56-68 (2022).
3. Yost, J.L. & Hartemink, A.E. How deep is the soil studied – an analysis of four soil science journals. *Plant Soil.* **452**, 5–18 (2020).
4. Zhang, T. Geocryology in China. *Permafr. Periglac. Process*. **12**, 315–322 (2001).
5. Saidi-Mehrabad, A. *et al*. Optimization of subsampling, decontamination, and DNA extraction of difficult peat and silt permafrost samples. *Sci Rep*. **31;10(1)**:14295 (2020)
6. Wen, Q. *et al*. Viral communities locked in high elevation permafrost up to 100 m in depth on the Tibetan Plateau. *Sci Total Environ*. **932**, 172829 (2024).
7. Gao, H. *et al*. Permafrost Hydrology of the Qinghai-Tibet Plateau: A Review of Processes and Modeling. *Front. Earth Sci*. **8**, 576838 (2021). doi: 10.3389/feart.2020.576838
8. Lu, Q., Zhao, D. & Wu, S. Simulated responses of permafrost distribution to climate change on the Qinghai–Tibet Plateau. *Sci Rep* **7,** 3845 (2017). https://doi.org/10.1038/s41598-017-04140-7
9. Mackelprang, R., *et al*. Microbial survival strategies in ancient permafrost: insights from metagenomics. *ISME J*. **11**, 2305–2318 (2017). https://doi.org/10.1038/ismej.2017.93
10. Jansson, J.K. & Taş, N. The microbial ecology of permafrost. *Nat. Rev. Microbiol*. **12**, 414–425 (2014).
11. Krawczyk, P.S., Lipinski, L. & Dziembowski, A. PlasFlow: predicting plasmid sequences in metagenomic data using genome signatures. *Nucleic Acids Res*. **46,** e35 (2018).
