# Supplementary material for: Depth-resolved transcriptional activity of antibiotic resistance genes in deep permafrost of the Qinghai–Tibet Plateau
Source: Commun Biol. 2026 Jun 30;9:1374. doi: 10.1038/s42003-026-10593-2 (PMC13683339; doi:10.1038/s42003-026-10593-2)
Supplement: Supplementary file 2 [file 42003_2026_10593_MOESM2_ESM.pdf]

Description of Additional Supplementary files

File name: Supplementary Data 1

Description: Identification and annotation of mobile genetic elements (MGEs) in individual samples from permafrost core TTH-04.

File name: Supplementary Data 2

Description: Identification and annotation of mobile genetic elements (MGEs) in individual samples from permafrost core TTH-07.

File name: Supplementary Data 3

Description: Trace metal concentrations across depth-resolved permafrost samples and results of Kruskal–Wallis tests followed by Dunn's post hoc multiple comparison analyses.

File name: Supplementary Data 4

Description: Numerical source data underlying all graphs and charts presented in the main manuscript figures (Figures 1–8).

File name: Supplementary Data 5

Description: Numerical source data underlying all supplementary figures, including datasets for metabolic genes, microbial phyla, and polymerdegrading genes.
