# Supplementary material for: Depth-resolved transcriptional activity of antibiotic resistance genes in deep permafrost of the Qinghai–Tibet Plateau
Source: Commun Biol. 2026 Jun 30;9:1374. doi: 10.1038/s42003-026-10593-2 (PMC13683339; doi:10.1038/s42003-026-10593-2)
Supplement: Supplementary file 3 [file 42003_2026_10593_MOESM8_ESM.pdf]

nature portfolio

Corresponding author(s): Shichang Kang

Last updated by author(s): 2026-23-2

Reporting Summary

Nature Portfolio wishes to improve the reproducibility of the work that we publish. This form provides structure for consistency and transparency in reporting. For further information on Nature Portfolio policies, see our Editorial Policies and the Editorial Policy Checklist.

Please do not complete any field with "not applicable" or n/a. Refer to the help text for what text to use if an item is not relevant to your study.
For final submission: please carefully check your responses for accuracy; you will not be able to make changes later.

Statistics

For all statistical analyses, confirm that the following items are present in the figure legend, table legend, main text, or Methods section.

n/a	Confirmed	
☐	☑	The exact sample size (*n*) for each experimental group/condition, given as a discrete number and unit of measurement
☐	☑	A statement on whether measurements were taken from distinct samples or whether the same sample was measured repeatedly
☐	☑	The statistical test(s) used AND whether they are one- or two-sided<br>*Only common tests should be described solely by name; describe more complex techniques in the Methods section.*
☐	☑	A description of all covariates tested
☐	☑	A description of any assumptions or corrections, such as tests of normality and adjustment for multiple comparisons
☐	☑	A full description of the statistical parameters including central tendency (e.g. means) or other basic estimates (e.g. regression coefficient) AND variation (e.g. standard deviation) or associated estimates of uncertainty (e.g. confidence intervals)
☐	☑	For null hypothesis testing, the test statistic (e.g. *F*, *t*, *r*) with confidence intervals, effect sizes, degrees of freedom and *P* value noted<br>*Give P values as exact values whenever suitable.*
☑	☐	For Bayesian analysis, information on the choice of priors and Markov chain Monte Carlo settings
☐	☑	For hierarchical and complex designs, identification of the appropriate level for tests and full reporting of outcomes
☐	☑	Estimates of effect sizes (e.g. Cohen's *d*, Pearson's *r*), indicating how they were calculated

Our web collection on statistics for biologists contains articles on many of the points above.

Software and code

Policy information about availability of computer code

Data collection	No custom software was used for data collection. Sequencing was performed using standard Illumina meta-transcriptomic protocols by a commercial sequencing provider, as described in the Methods.
Data analysis	Data were processed using established, publicly available bioinformatics software (MEGAHIT, Bowtie2, Samtools, Salmon, and custom scripts) as described in the Methods. No custom algorithms central to the study were developed.

For manuscripts utilizing custom algorithms or software that are central to the research but not yet described in published literature, software must be made available to editors and reviewers. We strongly encourage code deposition in a community repository (e.g. GitHub). See the Nature Portfolio guidelines for submitting code & software for further information.

Data

Policy information about availability of data

All manuscripts must include a data availability statement. This statement should provide the following information, where applicable:
- Accession codes, unique identifiers, or web links for publicly available datasets
- A description of any restrictions on data availability
- For clinical datasets or third party data, please ensure that the statement adheres to our policy

The sequencing data generated in this study have been deposited in the NCBI BioProject database under accession number PRJNA1251305, with Sequence Read Archive (SRA) accession numbers SRX28703910–SRX28703934, SRX28704801–SRX28704825, SRX28705162–SRX28705186, and SRX28746333–SRX28746357. Processed data and functional annotations are provided in the Supplementary Information.

Research involving human participants, their data, or biological material

Policy information about studies with human participants or human data. See also policy information about sex, gender (identity/presentation), and sexual orientation and race, ethnicity and racism.

Reporting on sex and gender	Not applicable in this study.
Reporting on race, ethnicity, or other socially relevant groupings	Not applicable in this study.
Population characteristics	Not applicable in this study.
Recruitment	Not applicable in this study.
Ethics oversight	Not applicable in this study.

Note that full information on the approval of the study protocol must also be provided in the manuscript.

Field-specific reporting

Please select the one below that is the best fit for your research. If you are not sure, read the appropriate sections before making your selection.

☐ Life sciences ☐ Behavioural & social sciences ☑ Ecological, evolutionary & environmental sciences

For a reference copy of the document with all sections, see nature.com/documents/nr-reporting-summary-flat.pdf

Life sciences study design

All studies must disclose on these points even when the disclosure is negative.

Sample size	
Data exclusions	
Replication	
Randomization	
Blinding	

Behavioural & social sciences study design

All studies must disclose on these points even when the disclosure is negative.

Study description	
Research sample	
Sampling strategy	
Data collection	
Timing	
Data exclusions	
Non-participation	
Randomization	

Ecological, evolutionary & environmental sciences study design

All studies must disclose on these points even when the disclosure is negative.

Study description	This is an observational, depth-resolved meta-transcriptomics study of antibiotic resistance genes expression in two deep permafrost cores (40 m and 100 m) from TuoTuo River Basin, QTP. Total of 25 stratified subsamples representing active layer, permafrost, and sub-permafrost horizons were analyzed. RNA was extracted and sequenced, and bio-informatics and statistical analysis were conducted in each core to assess depth-associated patterns. No exterminate manipulation was performed.
Research sample	Two deep permafrost cores (TTH-04: 40□m; TTH-07: 100□m) from the Tuotuo River Basin, Qinghai–Tibet Plateau were collected. Cores were stratified into active layer, permafrost, and sub-permafrost horizons, yielding 25 subsamples. Samples were collected under aseptic, frozen conditions for RNA preservation. Sampling, decontamination, and handling procedures are described in the Methods.
Sampling strategy	Cores were collected at representative sites to capture depth-resolved variation in permafrost layers. Within each core, stratified subsamples were taken from the active layer, permafrost, and sub-permafrost horizons. Subsamples were treated as independent units for statistical analysis. No randomization or blinding was applied, as this was an observational study of natural environmental gradients.
Data collection	Stratified subsamples from each permafrost core were aseptically collected and preserved at −40°C. Total RNA was extracted from each subsample, treated with DNase, and reverse-transcribed to cDNA. Libraries were prepared and sequenced using standard Illumina NovaSeq meta-transcriptomic protocols (Shown in Methods section). No custom software was used during sample collection.
Timing and spatial scale	Samples were collected during the frozen season (October–November 2020). Two permafrost cores were sampled at the Tuotuo River Basin, Qinghai–Tibet Plateau, with vertical stratification from 0 to 40□m (TTH-04) and 0 to 100□m (TTH-07). Subsamples represent depth-resolved horizons: active layer, permafrost, and sub-permafrost.
Data exclusions	No data were excluded from analyses. All collected subsamples that passed RNA quality control were included in meta-transcriptomic, bioinformatic, and statistical analyses.
Reproducibility	All analyses were performed on independent depth-resolved subsamples within each core. No technical replicates were used for sequencing; however, all processing steps (RNA extraction, library preparation, sequencing, and bioinformatics) followed standardized, reproducible protocols as described in the Methods.
Randomization	No randomization was applied, as this was an observational study of naturally stratified permafrost cores. Depth-defined subsamples were collected systematically along the core to capture vertical variation.
Blinding	Blinding was not applied, as sample identity (core and depth) was required for all analyses and interpretation.

Did the study involve field work? ☑ Yes ☐ No

Field work, collection and transport

Field conditions	Cores were collected from the Tuotuo River Basin, Qinghai–Tibet Plateau, at elevations of 4850□m (TTH-04) and 5005□m (TTH-07). TTH-04 was located in a low solar-exposure basin with gentle slopes and dense vegetation; TTH-07 was on a piedmont slope with higher solar exposure and moderate vegetation. Sampling was conducted during the frozen season (October–November 2020) under subzero temperatures and typical alpine permafrost conditions.
Location	Permafrost cores were collected from the Tuotuo River Basin, central Qinghai–Tibet Plateau, China (TTH-04: 91°30′3.71″E, 34°3′55.10″N; TTH-07: 91°21′21.32″E, 34°2′48.98″N).
Access & import/export	Sampling was conducted with permission from local authorities. Cores were transported under frozen conditions to the Northwest Institute of Eco-Environment and Resources (NIEER), Chinese Academy of Sciences, following national and institutional guidelines for the transport of environmental samples.
Disturbance	Sampling involved minimal physical disturbance. Only the extracted cores were removed, and the surrounding permafrost environment was largely undisturbed. No chemical or biological manipulation occurred in the field.

Reporting for specific materials, systems and methods

We require information from authors about some types of materials, experimental systems and methods used in many studies. Here, indicate whether each material, system or method listed is relevant to your study. If you are not sure if a list item applies to your research, read the appropriate section before selecting a response.

Materials & experimental systems

n/a	Involved in the study	
☑	☐	Antibodies
☑	☐	Eukaryotic cell lines
☑	☐	Palaeontology and archaeology
☑	☐	Animals and other organisms
☑	☐	Clinical data
☑	☐	Dual use research of concern
☑	☐	Plants

Methods

n/a	Involved in the study	
☑	☐	ChIP-seq
☑	☐	Flow cytometry
☑	☐	MRI-based neuroimaging

Antibodies

Antibodies used	
Validation	

Eukaryotic cell lines

Policy information about cell lines and Sex and Gender in Research

Cell line source(s)

Authentication

Mycoplasma contamination

Commonly misidentified lines
(See ICLAC register)

Palaeontology and Archaeology

Specimen provenance

Specimen deposition

Dating methods

☐ Tick this box to confirm that the raw and calibrated dates are available in the paper or in Supplementary Information.

Ethics oversight

Note that full information on the approval of the study protocol must also be provided in the manuscript.

Animals and other research organisms

Policy information about studies involving animals; ARRIVE guidelines recommended for reporting animal research, and Sex and Gender in Research

Laboratory animals

Wild animals

Reporting on sex

Field-collected samples

Ethics oversight

Note that full information on the approval of the study protocol must also be provided in the manuscript.

Clinical data

Policy information about clinical studies

All manuscripts should comply with the ICMJE guidelines for publication of clinical research and a completed CONSORT checklist must be included with all submissions.

Clinical trial registration

Study protocol

Data collection

Outcomes

Dual use research of concern

Policy information about dual use research of concern

Hazards

Could the accidental, deliberate or reckless misuse of agents or technologies generated in the work, or the application of information presented in the manuscript, pose a threat to:

No | Yes

- [ ] [ ] Public health
- [ ] [ ] National security
- [ ] [ ] Crops and/or livestock
- [ ] [ ] Ecosystems
- [ ] [ ] Any other significant area

Experiments of concern

Does the work involve any of these experiments of concern:

No | Yes

- [ ] [ ] Demonstrate how to render a vaccine ineffective
- [ ] [ ] Confer resistance to therapeutically useful antibiotics or antiviral agents
- [ ] [ ] Enhance the virulence of a pathogen or render a nonpathogen virulent
- [ ] [ ] Increase transmissibility of a pathogen
- [ ] [ ] Alter the host range of a pathogen
- [ ] [ ] Enable evasion of diagnostic/detection modalities
- [ ] [ ] Enable the weaponization of a biological agent or toxin
- [ ] [ ] Any other potentially harmful combination of experiments and agents

Plants

Seed stocks

Novel plant genotypes

Authentication

ChIP-seq

Data deposition

- [ ] Confirm that both raw and final processed data have been deposited in a public database such as GEO.
- [ ] Confirm that you have deposited or provided access to graph files (e.g. BED files) for the called peaks.

Data access links
May remain private before publication.

Files in database submission

Genome browser session
(e.g. UCSC)

Methodology

Replicates

Sequencing depth

Antibodies

Peak calling parameters

Data quality

Software

Flow Cytometry

Plots

Confirm that:

☐ The axis labels state the marker and fluorochrome used (e.g. CD4-FITC).

☐ The axis scales are clearly visible. Include numbers along axes only for bottom left plot of group (a 'group' is an analysis of identical markers).

☐ All plots are contour plots with outliers or pseudocolor plots.

☐ A numerical value for number of cells or percentage (with statistics) is provided.

Methodology

Sample preparation

Instrument

Software

Cell population abundance

Gating strategy

☐ Tick this box to confirm that a figure exemplifying the gating strategy is provided in the Supplementary Information.

Magnetic resonance imaging

Experimental design

Design type

Design specifications

Behavioral performance measures

Imaging type(s)

Field strength

Sequence & imaging parameters

Area of acquisition

Diffusion MRI ☐ Used ☐ Not used

Preprocessing

Preprocessing software

Normalization

Normalization template

Noise and artifact removal

Volume censoring

Statistical modeling & inference

Model type and settings

Effect(s) tested

Specify type of analysis: ☐ Whole brain ☐ ROI-based ☐ Both

Statistic type for inference

(See Eklund et al. 2016)

Correction

Models & analysis

n/a	Involved in the study
☐	☐ Functional and/or effective connectivity
☐	☐ Graph analysis
☐	☐ Multivariate modeling or predictive analysis

Functional and/or effective connectivity

Graph analysis

Multivariate modeling and predictive analysis

This checklist template is licensed under a Creative Commons Attribution 4.0 International License, which permits use, sharing, adaptation, distribution and reproduction in any medium or format, as long as you give appropriate credit to the original author(s) and the source, provide a link to the Creative Commons license, and indicate if changes were made. The images or other third party material in this article are included in the article's Creative Commons license, unless indicated otherwise in a credit line to the material. If material is not included in the article's Creative Commons license and your intended use is not permitted by statutory regulation or exceeds the permitted use, you will need to obtain permission directly from the copyright holder. To view a copy of this license, visit http://creativecommons.org/licenses/by/4.0/
