# Supplementary material for: Depth-resolved transcriptional activity of antibiotic resistance genes in deep permafrost of the Qinghai–Tibet Plateau
Source: Commun Biol. 2026 Jun 30;9:1374. doi: 10.1038/s42003-026-10593-2 (PMC13683339; doi:10.1038/s42003-026-10593-2)
Supplement: Supplementary file 4 [file 42003_2026_10593_MOESM9_ESM.pdf]

nature portfolio

Peer Review File

Depth-resolved transcriptional activity of antibiotic resistance genes in deep permafrost of the Qinghai–Tibet Plateau

Corresponding Author: Professor Shichang Kang

This file contains all reviewer reports in order by version, followed by all author rebuttals in order by version.

Version 0:

Reviewer comments:

Reviewer #1

(Remarks to the Author)
Highlights: Sajjad et al. highlight a major knowledge gap in permafrost systems regarding antibiotic resistance genes (ARGs), not from a metagenomic perspective, but from a metatranscriptomic perspective. This approach is challenging but has the potential to answer critical questions and bypass the issues associated with relic DNA. The study is unique and valuable; however, it contains a number of caveats—some are understandable given the system, while others would benefit from more thorough explanation. Overall, although this work represents a local phenomenon, it is highly promising and could pave the way for hypothesis development and future analyses in permafrost microbiomes. For example, studies of regions such as Beringia, which represent true relics of the Ice Age, could greatly benefit from similar metatranscriptomic approaches.

Overall, this study addresses a major knowledge gap by exploring ARGs in permafrost from a metatranscriptomic perspective. While unique and promising, the manuscript requires major revisions.

My Review
Main text:
Line 60: Recent studies have demonstrated microbial activity under a range of subzero temperatures. The authors should acknowledge this body of work, as the field has already established that microbial activity can occur below 0 °C. For example, see Tuorto et al., Bacterial genome replication at subzero temperatures in permafrost, The ISME Journal (Tuorto SJ, Darias P, McGuinness LR, Panikov N, Zhang T, Häggblom MM, et al. Bacterial genome replication at subzero temperatures in permafrost. The ISME journal. 2014;8(1):139–49), which provides evidence of bacterial genome replication under subzero conditions. Incorporating discussion of such studies would better contextualize the current findings.

The Methods and Supplementary Methods sections (S1.1): refer to prior studies for permafrost stratigraphic and cryostratigraphic interpretations; however, several critical aspects that could directly or indirectly influence downstream meta-transcriptomic analyses are not sufficiently addressed: 1) It is unclear whether there is published evidence of past or present active-layer water infiltration into the underlying permafrost at the study site. Hydrologic transport can introduce contemporary solutes, dissolved gases, and potentially mobile microorganisms into deeper horizons. 2) The manuscript does not indicate whether there are stratigraphic or cryostructural signs of paleo–active layer mixing or reworking (e.g., cryoturbation, involutions, disrupted bedding). Such features could reflect past active-layer dynamics and may influence interpretations regarding the “indigenous” versus potentially introduced origin of recovered RNA signals. 3) The potential impact of past glacial cycles (e.g., local mountain glaciation or periglacial processes) on sediment deposition and permafrost formation is not discussed. Glacial or periglacial disturbances can lead to stratigraphic reworking, erosion, and secondary burial of younger organic material at depth. This has important implications for interpreting whether deeper horizons necessarily represent temporally “ancient” transcriptomic signals. 4) The manuscript lacks explicit information regarding the age of the analyzed permafrost samples. Establishing the age of material at specific depths is essential for placing transcriptomic results in a temporal framework.

In the Methods and Supplementary Methods sections (S1.1), several important contamination-related concerns that could directly affect transcriptomic interpretations are not adequately addressed. 1) It is unclear whether the authors considered the

potential effects of drilling-induced mechanical stress, temperature fluctuation, oxygen exposure, and pressure changes on microbial stress responses and downstream transcript profiles. These perturbations could induce rapid transcriptional changes, potentially confounding interpretation of in situ activity. 2) The sterilization/decontamination approach described appears insufficiently justified. Mechanical scraping or surface sterilization alone has been shown to be inadequate for removing exogenous contamination in low-biomass permafrost samples. For example, Saidi-Mehrabad et al. (Saidi-Mehrabad A, Neuberger P, Cavaco M, Froese D, Lanoil B. Optimization of subsampling, decontamination, and DNA extraction of difficult peat and silt permafrost samples. Scientific Reports. 2020 Aug 31;10(1):14295) demonstrated that certain decontamination methods can create microcracks and inadvertently push exogenous microbes deeper into the core material. The authors should discuss whether their method avoids these pitfalls and provide validation data (e.g., tracer tests, contamination monitoring). 3) The manuscript does not comprehensively describe post-decontamination subsampling procedures. Multiple studies have emphasized rigorous sterile workflows for low-biomass permafrost samples (Saidi-Mehrabad A, Neuberger P, Cavaco M, Froese D, Lanoil B. Optimization of subsampling, decontamination, and DNA extraction of difficult peat and silt permafrost samples. Scientific Reports. 2020 Aug 31;10(1):14295; D'Costa VM, King CE, Kalan L, Morar M, Sung WW, Schwarz C, Froese D, Zazula G, Calmels F, Debruyne R, Golding GB. Antibiotic resistance is ancient. Nature. 2011 Sep 22;477(7365):457-61). 4) In Figure S8, the right panel appears to show a core being handled with bare hands. If accurate, this raises serious contamination concerns. Additionally, the left panel suggests that the core material is friable and crumbling, which increases the likelihood that surface contamination could penetrate deeper into the sample. Given the extremely low biomass typical of deep permafrost, even minor contamination could significantly influence downstream meta-transcriptomic results. The permafrost microbiology community strongly emphasizes contamination assessment and validation, especially for ancient or deep cores.

S1.2 RNA Extraction: Quantification of residual DNA using the Qubit DNA HS assay alone may not be sufficient to confirm complete removal of amplifiable genomic DNA, particularly in low-biomass permafrost samples. The authors should clarify whether RT-minus controls, qPCR-based validation, or other functional assessments were performed to verify the absence of residual DNA capable of contributing to downstream sequencing results.
Results section, line 88: The authors repeatedly emphasize that their observations are limited to the sampled cores and are depth-resolved. However, the manuscript lacks critical contextual information, including the type of permafrost studied (e.g., ice and organic matter content) as well as the age of the cores. Providing these details is essential for interpreting microbial and transcriptomic results. Additionally, the sequencing data reported in Table S1 show very high depth for deep permafrost meta-transcriptomics (60–100 million clean reads per sample). While such depth is not inherently inappropriate, justification is needed given the extremely low biomass and RNA yields typical of deep permafrost. Some samples also exhibit substantial reductions from raw to clean reads, suggesting aggressive filtering or potentially low-quality libraries. Finally, although most samples have GC content around 54–56%, a few outliers (e.g., 46–63%) may reflect compositional heterogeneity, technical artifacts, or potential contamination, which should be addressed or discussed.

Line 96: It is not clear under what conditions RNA was extracted. Were the cores fully thawed, partially thawed, or kept frozen? Was extraction performed in a refrigerated BSL hood or cold room? If thawing occurred, how do the authors account for potential transcription induced by thaw or handling? How confident can the authors be that the detected transcripts reflect in situ activity at the time of permafrost deposition rather than being generated in response to sampling and extraction?

Lines 103–112: Did the authors consider that some of the observed changes might result from necromass degradation or microbial responses to thaw? The manuscript reports depth-dependent stratification of ARG transcripts across both cores, with aminoglycoside, β-lactam, puromycin, and multidrug resistance genes showing distinct peaks at various depths. While these patterns are biologically plausible, it remains unclear to what extent the detected transcripts reflect active microbial expression versus RNA derived from necromass degradation or transcription induced by thaw. Necromass can be slowly degraded even under subzero permafrost conditions, potentially releasing nucleic acids and supporting transcription in surviving microbes (Zhou W, Ma T, Yin X, Wu X, Li Q, Rupakheti D, Xiong X, Zhang Q, Mu C, de Foy B, Rupakheti M. Dramatic carbon loss in a permafrost thaw slump in the Tibetan Plateau is dominated by the loss of microbial necromass carbon. Environmental Science & Technology. 2023 Apr 19;57(17):6910–21; Coolen MJ, Orsi WD. The transcriptional response of microbial communities in thawing Alaskan permafrost soils. Frontiers in Microbiology. 2015 Mar 16;6:197). Likewise, thawing itself or the presence of labile organic matter can trigger microbial transcription independent of the original in situ activity (Orsi WD, 2015). This process could contribute to localized ARG transcript peaks, particularly at depths with higher nutrient availability, redox variation, or preserved organic matter.

Lines 124-133: The reported depth-dependent variation in resistance mechanisms raises an ecological question that warrants further clarification. In dynamic surface soils such as the active layer, recurrent freeze–thaw cycles, periodic inputs of fresh organic matter, and higher microbial turnover are expected to intensify microbial competition and selective pressures. Experimental and ecological studies have shown that competitive microbial growth strategies in soils are associated with increased ARG abundance and diversification (Liu Z, Yao X, Chen C, Zhao Y, Dong C, Sun L, Zhao J, Zhang B, Yu Z, Cheng D, Zhu L. Growth of microbes in competitive lifestyles promotes increased ARGs in soil microbiota: insights based on genetic traits. Microbiome. 2025 Jan 13;13(1):8). In contrast, deep permafrost communities are typically structured by long-term environmental stability, low energy flux, and strong abiotic filtering, where functional gene distributions may reflect legacy signals and environmental constraints rather than active competitive dynamics (Waldrop MP, Chabot CL, Liebner S, Holm S, Snyder MW, Dillon M, Dudgeon SR, Douglas TA, Leewis MC, Walter Anthony KM, McFarland JW. Permafrost microbial communities and functional genes are structured by latitudinal and soil geochemical gradients. The ISME Journal. 2023 Aug;17(8):1224-35). Additionally, studies of ecological succession in glacial forefields demonstrate that ARG prevalence shifts with environmental selection and community turnover (Gou Y, Liu Y, Hu A, Mao G, Dong R, Li S, Liu P, Liu Y, Ji M. Dissemination of genes associated with antibiotic resistance and bacterial virulence during ecosystem succession in two Tibetan glacier forefields. Science of The Total Environment. 2025 Feb 1;963:178514). Given

this ecological framework, it would be helpful for the authors to clarify whether the higher diversity or mechanistic breadth of ARGs observed in specific layers aligns with expected competitive dynamics in the active layer versus the comparatively stable, energy-limited permafrost strata. If deeper layers show equal or greater mechanistic diversity, additional explanation is needed to distinguish between active in situ expression, legacy functional potential, or methodological artifacts.

Please ensure that all gene names are consistently italicized throughout the manuscript in accordance with standard nomenclature conventions. See https://insight.jci.org/kiosk/publish/genestyle?utm_source=chatgpt.com

Lines 146-148: It would be helpful if the authors could report the measured concentrations of mercury (Hg) and copper (Cu) in the analyzed permafrost samples and directly compare them with those in the corresponding active-layer samples. Providing these quantitative data would allow readers to better assess potential metal-driven co-selection of ARGs. While elevated metal concentrations can contribute to resistance selection, it should be noted that high mercury levels are not universally characteristic of permafrost environments (e.g. see Zhao Y, Hu HW, Su JQ, Hao X, Guo H, Liu YR, Zhu YG. Influence of legacy mercury on antibiotic resistomes: evidence from agricultural soils with different cropping systems. Environmental science & technology. 2021 Oct 6;55(20):13913-22.; Antcibor I, Eschenbach A, Zubrzycki S, Kutzbach L, Bolshiyanov D, Pfeiffer EM. Trace metal distribution in pristine permafrost-affected soils of the Lena River delta and its hinterland, northern Siberia, Russia. Biogeosciences. 2014 Jan 2;11(1):1-5.; Perryman CR, Wirsing J, Bennett KA, Brennick O, Perry AL, Williamson N, Ernakovich JG. Heavy metals in the Arctic: Distribution and enrichment of five metals in Alaskan soils. PLoS One. 2020 Jun 3;15(6):e0233297). Therefore, site-specific measurements are essential to support any interpretation linking ARG patterns to metal exposure.

Discussion, line 202: The reference to “pre-anthropogenic strata” may be valid; however, it still remains unclear whether the authors have considered potential stratigraphic reworking associated with past glacial maxima, periglacial processes, or the existence of paleo–active layers.

Lines 206-210: Lines 206–210: The statement that permafrost contains viable microorganisms is valid; however, it may oversimplify the ecological complexity of permafrost microbiomes. Permafrost soils are known to contain a mixture of culturable cells, viable but non-culturable cells, dormant cells, and dead biomass, and metabolic activity under in situ conditions is generally minimal and strongly constrained by extreme environmental stress. Previous studies have shown that permafrost microbial communities include viable but non-culturable cells and partially degraded “ghost” cells, reflecting a spectrum of survival states rather than sustained metabolic activity (e.g., Gilichinsky et al. 1989; Rivkina et al. 1998; Vishnivetskaya et al. 2000). Metagenomic analyses further suggest that long-term freezing selects for survival strategies such as dormancy, repair mechanisms, and stress responses rather than active proliferation, with functional potential declining with increasing permafrost age and environmental stress (Mackelprang et al. 2017). This is not intended as a criticism, but rather as contextual information that may help refine the discussion and more accurately frame the physiological state and activity potential of permafrost microbial communities.

Lines 215-217: While the general statement may be valid, it is difficult to justify without clear age constraints for the analyzed permafrost samples. The phrase “over geological timescales” is particularly broad and may overstate the temporal framework unless supported by explicit dating data.

Lines 225-227: The authors propose horizontal gene transfer (HGT) as a driver of ARG distribution; however, it is not clear how this process could occur in a system where microbial movement is extremely limited and cells are effectively trapped within a spatially constrained, frozen microenvironment. Permafrost communities are strongly shaped by dispersal limitation and physical constraints, which reduce cell–cell contact and limit opportunities for the primary mechanisms of HGT (conjugation, transduction, transformation) that typically require mobility or interaction among organisms (e.g., Bottos EM, Kennedy DW, Romero EB, Fansler SJ, Brown JM, Bramer LM, Chu RK, Tfaily MM, Jansson JK, Stegen JC. Dispersal limitation and thermodynamic constraints govern spatial structure of permafrost microbial communities. FEMS microbiology ecology. 2018 Aug;94(8):fiy110.). While evidence of HGT exists in deep subsurface environments under slow growth conditions (e.g., Labonté JM, Field EK, Lau M, Chivian D, Van Heerden E, Wommack KE, Kieft TL, Onstott TC, Stepanauskas R. Single cell genomics indicates horizontal gene transfer and viral infections in a deep subsurface Firmicutes population. Frontiers in microbiology. 2015 Apr 22;6:349.), these environments still maintain some degree of microbial activity and interaction that may not be comparable to permanently frozen permafrost. Given the strong physical and ecological constraints on microbial mobility in frozen soils, the authors should clarify the ecological plausibility of frequent HGT and discuss whether alternative explanations (e.g., ancient HGT events preserved in genomes, relic DNA inheritance, or slow selective retention over evolutionary timescales) might better account for the observed ARG patterns.

Lines 256-258: Did the authors consider using models specifically designed for sparse count data, such as BAMZINB (Hajihosseini M, Amini P, Saidi-Mehrabad A, Dinu I. Infants’ gut microbiome data: A Bayesian Marginal Zero-inflated Negative Binomial regression model for multivariate analyses of count data. Computational and Structural Biotechnology Journal. 2023;21:1621–1629), to test for relationships between environmental variables and microbial taxa? BAMZINB is particularly well suited for highly sparse microbiome datasets, as it accounts for zero inflation and overdispersion, and could provide a more robust assessment of which taxa are associated with ARG abundances or environmental gradients in these permafrost samples.

Lines 265-267: Does this statement align with the context of the Stress Gradient Hypothesis (Hernandez DJ, David AS, Menges ES, Searcy CA, Afkhami ME. Environmental stress destabilizes microbial networks. The ISME Journal. 2021;15(6):1722–1734)?

Line 272: The authors should refrain from using outdated nomenclature. Please update Firmicutes to Bacillota and Euryarchaeota to Methanobacteriota throughout the manuscript. For accuracy, the authors may consult the DSMZ LPSN database to verify current taxonomic names and ensure consistent usage.

Line 275: change the Proteobacteria to Pseudomonadota.

Reviewer #2

(Remarks to the Author)
The study system is interesting and logistically challenging, however there are some key methodological and analytical limitations requiring substantial revision of the statistical approach.

1) The number of (pseudo)replicates per core and per layer type is not clear. This is important in determining how many samples (group sizes) were used for comparison of permafrost horizons to evaluate the robustness and balance of the statistical tests.

2) From Supplementary: "Within each core, multiple stratified subsamples from the active layer, permafrost, and sub-permafrost horizons were treated as replicate units to evaluate within-core, depth-resolved patterns."
I understand that this study is logistically difficult in terms of gathering the samples. Nevertheless, grouping across depth layers into permafrost horizons could have been avoided (though I understand the initial desire to do this to simplify interpretation). The current subsamples within each core are treated as independent replicates, despite being along a continuous gradient. Using depth as a continuous variable would have been more statistically robust for analysis of ARG abundances/diversity trends/networks than collapsing samples into three permafrost horizons (active, permafrost, sub-permafrost). Or at least controlling for depth. Grouping samples into discrete permafrost horizons collapses a continuous gradient into categorical comparisons. Though this would not fully fix the pseudo-replication issue of subsampling within the same cores, it would strengthen the analysis interpretations. Even if this is a site-specific study, the statistical treatment of the data is still not fully appropriate for the type of data, and this affects the reliability of the conclusions.

3) The negative and no-RT controls were not sequenced because there was not sufficient input for the library preparation (Supplementary data). However, for these types of low-biomass samples, it is important to sequence negative controls to identify any potential sources of contamination, even with insufficient/low input for library prep.

4) It does not seem like correction for multiple testing was performed with Pearson correlations, Mantel tests, network assembly (correction for multiple tests only done for: "Differential ARG expression across layers was evaluated in STAMP using Wilcoxon rank-sum tests with Benjamini–Hochberg FDR correction" -line 475)? Lack multiple test correction across all statistical tests in such a dataset (already exposed to pseudo replication) may result in large number of reported false positives and overinterpretation.

5) The authors report metatranscriptomic data but there is very limited interpretation of the broader "active" microbial community beyond ARG-centric analyses. As ARG expression is dependent on the microbial taxa expressing these genes, the direct lack of integration between community composition and ARG patterns limits biological interpretation. It remains unclear whether the reported permafrost horizon patterns reflect shifts in active microbial taxa.

6) The TPM normalization performed in this study allows the relative comparison of ARGs within the transcript pool, it does not capture absolute transcript abundance or total activity. Therefore, throughout the manuscript, statements claiming increased ARG activity and abundance across depth layers (permafrost horizons) may be an overinterpretation of the current data. These results could be interpreted as shifts in relative expression instead of absolute differences. This needs to be reworked beyond adding a simple caveat.

Reviewer #3

(Remarks to the Author)
Sajjad and colleagues investigated depth-resolved metatranscriptomes from two sampling cores bridging the active layer, permafrost and sub-permafrost, focusing on MTGs/ARGs expressed in the different layers and their potential origins like viruses or Transposons. The manuscript is very well written, with a trough of detail even including additional supplementary methods and reasoned out findings and conclusions. I do not really have any major concerns about this study but have listed a few suggestions below.

minor comments

Affiliation section – Monitoring / Institution (there are two typos in the affiliations)
L16 - As the analysis is
L86-88 – Switching between storage space size and read counts makes it more difficult to relate the read counts per sample and the total storage space. I'd consider making the measures uniform (or at least give total read length across all samples instead of storage space size) to make it easier to relate the numbers.

L394- 396 – a link or reference to these established protocols would be good to add as without them, the analyses are not really reproducible.
L406 – add product/article number
L407 – im curious as to the reasoning behind focusing on nonpolyadenylated transcripts – is that just a technical reason (i.e., the RTase you use excludes them) or is there a scientific reason?
L409 – add NovaSeq instrument identifier (X / 6000 / …); Also specify that you did not do any type of rRNA depletion or similar (that's my assumption as it isnt mentioned) and the library prep kit.
L417 – maybe a bit more specification than "~30-40" is possible? E.g., via mean and SD % per AMG database categories covered by your data or so? Or give a single number with "~" as it already implies a range around the given value.
L442 – please ensure that all statistical tests done within the tutools platform are described in sufficient detail to allow reproducing in more open-source platforms like R. To this end, please specify numbers of permutations, p-value adjustment, alpha error values used and so on (as I do not have an account on the tutools platform, I cannot really see what the defaults are and whether they are similar to alternatives in, e.g., R).

more a comment: To me it looks a lot nicer if the format of the affiliations/title/authors in the main and the SI are identical. I'd also make font+sizes uniform in the SI text passages.

Fig 3a – convert y axis fractions to percentages to match axis title

Supplemental data file names – The file names I got when downloading the zipped files are all indistinguishable (just a sequential number counting up). That makes it rather time-consuming to cross-reference your in-text references to, e.g., Table S3, as there is no file (and I also don't see a sheet in any Excel file) with that name.

Reviewer #4

(Remarks to the Author)
The study addresses a clear core scientific question with prominent innovations, standardised technical methods, and sufficient data support. There are no scientific flaws or methodological errors. The main issues focus on the explanation of sample representativeness and optimization of result presentation. These can be improved through revisions.

1. Insufficient evaluation of sample size limitations and inadequate constraint on conclusion expression (Abstract: Lines 36-37; Introduction: Lines 82-83; Results: Lines 98-100; Discussion: Lines 345-347).Only two permafrost cores (TTH-04: 40 m; TTH-07: 100 m) were selected in the study without spatial replicate samples. Although the authors repeatedly mentioned that the results were site-specific, the constraints imposed by this limitation on the study's conclusions were not consistently emphasized when presenting key findings (e.g., "antibiotic inactivation is the dominant resistance mechanism in permafrost" and "deep permafrost acts as a reservoir of genetic novelty").

2.The abbreviation "MTGs" first appeared in Line 28 of the abstract without definition, and it was not explained as "metal tolerance genes" until Line 146 of the results section.

3.In the transcriptome analysis, the selection basis of k-mer (21–141) for MEGAHIT assembly was not stated, and whether gene length was considered in the TPM normalization of Salmon quantification was not explained.

4.The number of ARG subtypes marked in the UpSet plot of Figure 3c was inconsistent with the calculation results of "40% (TTH-04) and 34.6% (TTH-07) of ARGs were shared" in Lines 165-167 of the text, and the coordinate axis was unlabeled with unclear numerical meaning.

5.Adonis P=0.28 (no statistical significance) in the PCoA analysis of TTH-04 in Figure 4a, but the text still stated that "β-diversity analysis confirmed depth-specific ARG assemblages" in Lines 162-164, which was contradictory to the figure results.

6.There was no correlation between depth and ARG abundance in TTH-07 in Figure 5a (R=-0.075, P=0.79), but this result was not mentioned in Lines 170-172 of the text, and only the correlation in TTH-04 was emphasized, resulting in selective data reporting.

Version 1:

Reviewer comments:

Reviewer #2

(Remarks to the Author)
The authors have addressed my comments. I would like to suggest that in future studies they do sequence negative controls despite insufficient material. It is still possible to make libraries with 'undetected' nucleic acids in negative controls and sequence them (you treat it as if you have enough material).

Reviewer #4

(Remarks to the Author)
The authors have adequately addressed all of my previous comments. I have no further concerns and support the publication of this manuscript.

Open Access This Peer Review File is licensed under a Creative Commons Attribution 4.0 International License, which permits use, sharing, adaptation, distribution and reproduction in any medium or format, as long as you give appropriate credit to the original author(s) and the source, provide a link to the Creative Commons license, and indicate if changes were made.
In cases where reviewers are anonymous, credit should be given to 'Anonymous Referee' and the source.
The images or other third party material in this Peer Review File are included in the article's Creative Commons license, unless indicated otherwise in a credit line to the material. If material is not included in the article's Creative Commons license and your intended use is not permitted by statutory regulation or exceeds the permitted use, you will need to obtain permission directly from the copyright holder.
To view a copy of this license, visit https://creativecommons.org/licenses/by/4.0/

Cover Statement to the Editor and Reviewers

Dear Editor and Reviewers,

We sincerely thank the Editor and all reviewers for their careful evaluation of our manuscript entitled: "Depth-resolved transcriptional activity of antibiotic resistance genes in deep permafrost of the Qinghai–Tibet Plateau."

We greatly appreciate the constructive comments and suggestions, which have significantly improved the quality, clarity, and rigor of the manuscript. We have carefully revised the manuscript accordingly, and all modifications are highlighted in yellow in the revised version.

Below, we provide a detailed point-by-point response to each reviewer's comment.

Sincerely,

Shichang Kang

On behalf of all authors

Reviewers' comments:

Reviewer #1 (Remarks to the Author):

Highlights: Sajjad et al. highlight a major knowledge gap in permafrost systems regarding antibiotic resistance genes (ARGs), not from a metagenomic perspective, but from a metatranscriptomic perspective. This approach is challenging but has the potential to answer critical questions and bypass the issues associated with relic DNA. The study is unique and valuable; however, it contains a number of caveats—some are understandable given the system, while others would benefit from more thorough explanation. Overall, although this work represents a local phenomenon, it is highly promising and could pave the way for hypothesis development and future analyses in permafrost microbiomes. For example, studies of regions such as Beringia, which represent true relics of the Ice Age, could greatly benefit from similar metatranscriptomic approaches.

Overall, this study addresses a major knowledge gap by exploring ARGs in permafrost from a metatranscriptomic perspective. While unique and promising, the manuscript requires major revisions.

Response: *We sincerely thank the reviewer for their thoughtful and constructive evaluation of our manuscript. We appreciate the recognition of the novelty and potential of applying a metatranscriptomic approach to investigate antibiotic resistance genes (ARGs) in deep permafrost systems. We agree that, given the complexity and low-biomass nature of this environment, several methodological and interpretative aspects require clearer explanation.*

In response, we have substantially revised the manuscript to improve methodological transparency, strengthen contextualization with existing literature, and refine interpretation of the results (yellow highlighted in revised MS). We have also carefully addressed each specific concern raised, particularly regarding contamination control, stratigraphic context, and interpretation of transcriptional signals.

Below, we provide a detailed, point-by-point response to each comment.

My Review

Main text:

Line 60: Recent studies have demonstrated microbial activity under a range of subzero temperatures. The authors should acknowledge this body of work, as the field has already established that microbial activity can occur below 0 °C. For example, see Tuorto et al., Bacterial genome replication at subzero temperatures in permafrost, The ISME Journal (Tuorto SJ, Darias P, McGuinness LR, Panikov N, Zhang T, Häggblom MM, et al. Bacterial genome replication at subzero temperatures in permafrost. The ISME journal. 2014;8(1):139–49), which provides evidence of bacterial genome replication under subzero conditions. Incorporating discussion of such studies would better contextualize the current findings.

Response: *We thank the reviewer for this important suggestion. We agree that microbial activity under subzero conditions is well established. We have revised the Introduction to acknowledge this body of work and now cite Tuorto et al. (2014) as an example demonstrating bacterial genome replication at subzero temperatures, thereby improving the contextualization of our study. Page# 3: Lines 62-64.*

The Methods and Supplementary Methods sections (S1.1): refer to prior studies for permafrost stratigraphic and cryostratigraphic interpretations; however, several critical aspects that could directly or indirectly influence downstream meta-transcriptomic analyses are not sufficiently addressed: 1) It is unclear whether there is published evidence of past or present active-layer water infiltration into the underlying permafrost at the study site. Hydrologic transport can introduce contemporary solutes, dissolved gases, and potentially mobile microorganisms into deeper horizons. 2) The manuscript does not indicate whether there are stratigraphic or cryostructural signs of paleo–active layer mixing or reworking (e.g., cryoturbation, involutions, disrupted bedding). Such features could reflect past active-layer dynamics and may influence interpretations regarding the "indigenous" versus potentially introduced origin of recovered RNA signals. 3) The potential impact of past glacial cycles (e.g., local mountain glaciation or periglacial processes) on sediment deposition and permafrost formation is not discussed. Glacial or periglacial disturbances can lead to stratigraphic reworking, erosion, and secondary burial of younger organic material at depth. This has important implications for interpreting whether deeper horizons necessarily represent temporally "ancient" transcriptomic signals. 4) The manuscript lacks explicit information regarding the age of the analyzed permafrost samples. Establishing the age of material at specific depths is essential for placing transcriptomic results in a temporal framework.

Response: *We thank the reviewer for these insightful and important comments regarding the stratigraphic context, depositional history, and temporal interpretation of the analyzed permafrost cores. We agree that these factors are critical for interpreting depth-resolved molecular signals and have revised the manuscript to provide clearer contextualization and to avoid overinterpretation. We have revised the manuscript to more explicitly address these considerations in both the Supplementary Methods and the Discussion.*

1) Hydrologic infiltration

Regarding potential active-layer water infiltration, the study sites are located in continuous permafrost within the Tuotuo River Basin on the Qinghai–Tibet Plateau, where permafrost is widely recognized to act as an aquiclude and low permeability due to ice-rich conditions that limit vertical water percolation and promote predominantly supra-permafrost flow within the active layer (Gao et al., 2021; Lu et al., 2017). However, direct hydrological measurements were not conducted in this study, and subsurface hydrological connectivity cannot be completely excluded, particularly in the presence of taliks or heterogeneous permafrost structures. Page# 8: lines 274-293: Page# 12: Lines 442-445 in main text. S1.1 Sample Collection and Contamination Control, Lines 116-126.

Gao H, Wang J, Yang Y, Pan X, Ding Y and Duan Z (2021) Permafrost Hydrology of the Qinghai-Tibet Plateau: A Review of Processes and Modeling. Front. Earth Sci. 8:576838. doi: 10.3389/feart.2020.576838

Lu, Q., Zhao, D. & Wu, S. Simulated responses of permafrost distribution to climate change on the Qinghai–Tibet Plateau. Sci Rep 7, 3845 (2017). https://doi.org/10.1038/s41598-017-04140-7

2) Cryoturbation / paleo-mixing

With respect to cryoturbation and paleo–active layer disturbance, field observations during drilling did not indicate evidence of large-scale stratigraphic disruption. However, we now explicitly note that finer-scale or historical cryoturbation and mixing processes cannot be ruled out and may influence the distinction between indigenous deep signals and potentially redistributed material from shallower horizons. Page# 8: Lines 274-279. Page# 14: Lines 513-515 in main text. S1.1 Sample Collection and Contamination Control, Lines 125-129.

3) Glacial/periglacial history

We agree that past glacial and periglacial processes may have influenced sediment deposition and permafrost formation in the region. We have revised the manuscript to explicitly acknowledge that Quaternary climatic fluctuations, including glacial and periglacial activity, may have contributed to sediment reworking, erosion, and secondary burial of organic material. These processes could affect the stratigraphic integrity of the cores and should be considered when interpreting depth-resolved transcriptomic signals, particularly with respect to distinguishing between in situ microbial activity and potentially reworked material. Previous cryostratigraphic studies have demonstrated that ancient permafrost deposits commonly contain thaw unconformities, erosional surfaces, cryoturbation features, and secondary depositional units that complicate direct age interpretation and may disrupt stratigraphic continuity (French & Shur, 2010; Murton et al., 2021). *Page# 8: Lines 277-279 in main text. S1.1 Sample Collection and Contamination Control, Lines 135-138.*

4) Age constraints

We clarify that direct radiometric dating of the sampled horizons was not performed, and therefore, absolute age constraints are not available. Accordingly, all interpretations referring to temporal scales (e.g., "ancient" or "long-term") are now explicitly framed in a relative, depth-stratified context rather than as absolute chronological inferences. We further acknowledge that establishing robust age-depth relationships in permafrost systems is inherently challenging because

cryostratigraphic sequences may reflect multiple depositional, freezing, thawing, and reworking events rather than continuous chronological accumulation. Previous studies have demonstrated that cryoturbation, thaw unconformities, sediment reworking, secondary burial, and refreezing processes can complicate chronological interpretation and disrupt stratigraphic continuity in permafrost deposits (French & Shur, 2010 (10.1016/j.earscirev.2010.04.002); Murton et al., 2021 (https://doi.org/10.1017/qua.2021.27); Opel et al., 2024 (10.1016/B978-0-323-99931-1.00258-0)). Accordingly, depth should not necessarily be interpreted as a direct proxy for sample age in the absence of site-specific geochronological constraints. This clarification is important to avoid overinterpretation of temporal signals in the absence of geochronological constraints. Page# 7: lines 220-222; Page#8: lines 275-277; Page# 14: Lines 515.

Collectively, these revisions ensure a more cautious and transparent interpretation of the data and emphasize that observed patterns should be understood within the limitations of stratigraphic uncertainty, potential sediment reworking, and unresolved temporal scaling.

In the Methods and Supplementary Methods sections (S1.1), several important contamination-related concerns that could directly affect transcriptomic interpretations are not adequately addressed. 1) It is unclear whether the authors considered the potential effects of drilling-induced mechanical stress, temperature fluctuation, oxygen exposure, and pressure changes on microbial stress responses and downstream transcript profiles. These perturbations could induce rapid transcriptional changes, potentially confounding interpretation of in situ activity. 2) The sterilization/decontamination approach described appears insufficiently justified. Mechanical scraping or surface sterilization alone has been shown to be inadequate for removing exogenous contamination in low-biomass permafrost samples. For example, Saidi-Mehrabad et al. (Saidi-Mehrabad A, Neuberger P, Cavaco M, Froese D, Lanoil B. Optimization of subsampling, decontamination, and DNA extraction of difficult peat and silt permafrost samples. Scientific Reports. 2020 Aug 31;10(1):14295) demonstrated that certain decontamination methods can create microcracks and inadvertently push exogenous microbes deeper into the core material. The authors should discuss whether their method avoids these pitfalls and provide validation data (e.g., tracer tests, contamination monitoring). 3) The manuscript does not comprehensively describe post-decontamination subsampling procedures. Multiple studies have emphasized rigorous sterile workflows for low-biomass permafrost samples (Saidi-Mehrabad A, Neuberger P, Cavaco M, Froese D, Lanoil B. Optimization of subsampling, decontamination, and DNA extraction of difficult peat and silt permafrost samples. Scientific Reports. 2020 Aug 31;10(1):14295; D'Costa VM, King CE, Kalan L, Morar M, Sung WW, Schwarz C, Froese D, Zazula G, Calmels F, Debruyne R, Golding GB. Antibiotic resistance is ancient. Nature. 2011 Sep 22;477(7365):457-61). 4) In Figure S8, the right panel appears to show a core being handled with bare hands. If accurate, this raises serious contamination concerns. Additionally, the left panel suggests that the core material is friable and crumbling, which increases the likelihood that surface contamination could penetrate deeper into the sample. Given the extremely low biomass typical of deep permafrost, even minor contamination could significantly influence downstream meta-

transcriptomic results. The permafrost microbiology community strongly emphasizes contamination assessment and validation, especially for ancient or deep cores.

Response: *We thank the reviewer for this detailed and important comment regarding potential contamination sources and handling-related biases in low-biomass permafrost meta-transcriptomic analyses. In response, we substantially revised Supplementary Methods S1.1 to provide a clearer and more transparent description of field handling, contamination control procedures, subsampling workflows, and limitations associated with deep permafrost sampling (Supplementary Methods S1.1; lines 54–85).*

First, we now clarify that drilling and subsampling were conducted during the peak freezing season, and all cores were maintained continuously frozen from retrieval through transport, storage, and laboratory processing. No thawing or incubation period was permitted prior to RNA stabilization or extraction. To minimize potential transcriptional responses induced by temperature fluctuation, oxygen exposure, or handling stress, subsampling for RNA analyses was performed immediately under frozen conditions using pre-cooled sterile instruments and dry-ice-supported workflows.

Second, we expanded the description of decontamination procedures and contamination monitoring. The outer 1–2 cm of each core was mechanically removed using sterile, single-use instruments, and only the uncontaminated inner-core material was used for downstream analyses. We acknowledge the reviewer's important point that mechanical scraping alone cannot eliminate contamination risk in low-biomass permafrost systems, and we now explicitly discuss this limitation in the revised Supplementary Methods, including reference to Saidi-Mehrabad et al. (2020) regarding the potential for microfracture-mediated contaminant penetration. Although tracer-based contamination tests were not performed, multiple contamination controls were included throughout field and laboratory workflows, including field blanks, extraction blanks, and no-reverse-transcription controls. No detectable nucleic acids or amplification signals were observed in these controls within the sensitivity limits of the applied assays.

Third, we clarified post-decontamination subsampling procedures by emphasizing that all RNA-related subsampling was conducted under sterile, RNase-free conditions within a Class II biological safety cabinet using single-use sterile consumables for each depth interval to minimize cross-contamination between horizons. These procedures are consistent with established workflows widely used in permafrost and low-biomass subsurface microbiology studies.

We also thank the reviewer for the observation regarding Figure S8. We further emphasize that field handling images were included only to provide contextual information on drilling logistics and core recovery in a high-altitude permafrost environment, and should not be interpreted as reflecting the sterile conditions applied during laboratory-based molecular work. All intact core handling for RNA-related analyses was conducted under strictly controlled laboratory conditions using sterile, RNase-free consumables within a Class II biosafety cabinet. Only the internal, uncontaminated portions of frozen cores were used for downstream processing, and all analytical subsampling was performed under aseptic conditions to minimize any risk of contamination. Collectively, these revisions provide a more transparent description of contamination control

procedures and acknowledge the inherent limitations associated with low-biomass permafrost sampling and RNA-based analyses. While contamination and handling artifacts can never be completely excluded in deep permafrost studies, the combination of continuous frozen-state handling, interior-core subsampling, multiple negative controls, and sterile laboratory workflows was designed to minimize these risks as much as practically possible.

S1.2 RNA Extraction: Quantification of residual DNA using the Qubit DNA HS assay alone may not be sufficient to confirm complete removal of amplifiable genomic DNA, particularly in low-biomass permafrost samples. The authors should clarify whether RT-minus controls, qPCR-based validation, or other functional assessments were performed to verify the absence of residual DNA capable of contributing to downstream sequencing results.

Response: *We thank the reviewer for this important comment regarding the validation of RNA-derived signals and potential residual genomic DNA contamination. We agree that rigorous validation is essential in low-biomass permafrost metatranscriptomic studies.*

In our workflow, RNA extracts were treated with DNase I prior to cDNA synthesis to remove residual genomic DNA. Remaining DNA was assessed using the Qubit DNA High Sensitivity assay, which indicated either undetectable levels or <5% residual DNA signal after DNase treatment. Page# 15; lines 539-540 and S1.2 RNA Extraction, DNA removal, and contamination controls; Lines 148-161.

In addition, RT-minus controls were performed during cDNA synthesis and showed no detectable amplification in representative samples, confirming effective removal of amplifiable genomic DNA. This information has been clarified in the revised manuscript (Page# 15; lines 539-540) and Supplementary Methods (S1.2) S1.2 RNA Extraction, DNA removal, and contamination controls; Lines 148-161. We acknowledge that in low-biomass permafrost systems, trace DNA contamination cannot be fully excluded; however, multiple independent controls support that its contribution to the observed transcriptomic profiles is minimal.

Results section, line 88: The authors repeatedly emphasize that their observations are limited to the sampled cores and are depth-resolved. However, the manuscript lacks critical contextual information, including the type of permafrost studied (e.g., ice and organic matter content) as well as the age of the cores. Providing these details is essential for interpreting microbial and transcriptomic results. Additionally, the sequencing data reported in Table S1 show very high depth for deep permafrost meta-transcriptomics (60–100 million clean reads per sample). While such depth is not inherently inappropriate, justification is needed given the extremely low biomass and RNA yields typical of deep permafrost. Some samples also exhibit substantial reductions from raw to clean reads, suggesting aggressive filtering or potentially low-quality libraries. Finally, although most samples have GC content around 54–56%, a few outliers (e.g., 46–63%) may reflect compositional heterogeneity, technical artifacts, or potential contamination, which should be addressed or discussed.

Repones:

1. Permafrost type, lithology, and contextual stratigraphy (Table S4)

We agree that lithological and cryostratigraphic context is essential for interpreting microbial and transcriptomic patterns. We have therefore expanded the manuscript to explicitly reference the detailed sedimentological and cryostructural characteristics of the studied cores.

In particular, we now clarify that the permafrost profiles represent highly heterogeneous, ice-rich systems comprising organic-rich surface layers and deeper silty, sandy, and sandstone units with variable cryostructures, including layered ice, ice lenses, vein ice, and ice inclusions. These features indicate strong vertical heterogeneity in sediment composition and ground ice distribution, as detailed in Supplementary Table S4. This additional context has been incorporated into the Study Sites and Sample Collection section to improve environmental interpretability.

2. Absence of absolute age constraints

We acknowledge that no direct geochronological dating was available for the studied cores. This limitation is now explicitly stated in both the Results and Discussion sections. We clarify that depth is used as a relative stratigraphic proxy rather than a strict temporal framework, and we have revised interpretations accordingly to avoid over-extrapolation of temporal dynamics. Page# 7: Lines 220-222: Page# 8: Lines 275-276: Page# 14: Lines 514. S1.1 Sample Collection and Contamination Control Lines 131-138.

3. High sequencing depth (60–100 million reads per sample)

We agree that the sequencing depth requires justification, given the low-biomass nature of deep permafrost samples. We now explicitly state in the Methods that deep sequencing was intentionally employed to ensure sufficient sensitivity for detecting low-abundance transcripts and to reduce stochastic sampling effects common in low-RNA environmental samples. Page# 15: Lines 543-545.

This clarification has been added directly in the sequencing description and is further supported by a reference to the Supplementary Methods (S1.3; Lines 183-196). We emphasize that such sequencing depth is standard practice in low-biomass environmental metatranscriptomic studies to improve transcript recovery and robustness of downstream quantification.

4. Reduction from raw to clean reads (filtering effects)

We clarify that reductions from raw to clean reads reflect standard and necessary bioinformatic processing steps, including adapter removal, quality trimming and low-complexity filtering. These filtering steps are particularly impactful in environmental RNA datasets due to the high proportion of degraded or non-target sequences. We now explicitly state this in the Methods (lines 547-549)/Supplementary Methods (197-206) to avoid misinterpretation as data loss or sequencing artifact.

5. GC content variability and outliers

We have added a dedicated clarification in the Supplementary Materials (S1.3 Sequencing and meta-transcriptomic analysis Lines 188-196) addressing GC variability. We explain that variation in GC content likely reflects depth-dependent microbial community heterogeneity and taxonomic shifts across stratified horizons. We further note that minor technical contributions cannot be excluded, including library preparation and amplification biases affecting GC-rich or GC-poor templates, as well as compositional shifts introduced during quality filtering. Given the

inherently low-biomass nature of deep permafrost samples, such variability is expected and does not necessarily indicate contamination or sequencing artifacts.

Line 96: It is not clear under what conditions RNA was extracted. Were the cores fully thawed, partially thawed, or kept frozen? Was extraction performed in a refrigerated BSL hood or cold room? If thawing occurred, how do the authors account for potential transcription induced by thaw or handling? How confident can the authors be that the detected transcripts reflect in situ activity at the time of permafrost deposition rather than being generated in response to sampling and extraction?

Response: *We thank the reviewer for this important methodological comment. We agree that clarification of RNA handling conditions is essential for interpreting transcript-based signals in permafrost systems. In the revised manuscript, we have expanded the Methods section to explicitly describe that all permafrost cores were maintained under frozen, cold-chain conditions throughout transport and processing, and that RNA extraction was conducted under RNase-free conditions in a pre-cooled laboratory with samples handled on dry ice. No thawing or incubation steps were allowed prior to RNA stabilization, thereby minimizing the possibility of transcriptional changes induced during handling. Page# 13-14: lines 489-496 and Supplementary Methods S1.1 lines 63-79.*

Additionally, we have added clarification in the Discussion/Limitations section acknowledging that, while these precautions minimize post-sampling artifacts, RNA signals in permafrost may still reflect a combination of in situ transcriptional activity and preserved or stabilized RNA fractions. Accordingly, our interpretations are framed in terms of transcriptional potential rather than instantaneous biological activity at the time of sampling. Lines 452-456

Lines 103–112: Did the authors consider that some of the observed changes might result from necromass degradation or microbial responses to thaw? The manuscript reports depth-dependent stratification of ARG transcripts across both cores, with aminoglycoside, β-lactam, puromycin, and multidrug resistance genes showing distinct peaks at various depths. While these patterns are biologically plausible, it remains unclear to what extent the detected transcripts reflect active microbial expression versus RNA derived from necromass degradation or transcription induced by thaw. Necromass can be slowly degraded even under subzero permafrost conditions, potentially releasing nucleic acids and supporting transcription in surviving microbes (Zhou W, Ma T, Yin X, Wu X, Li Q, Rupakheti D, Xiong X, Zhang Q, Mu C, de Foy B, Rupakheti M. Dramatic carbon loss in a permafrost thaw slump in the Tibetan Plateau is dominated by the loss of microbial necromass carbon. Environmental Science & Technology. 2023 Apr 19;57(17):6910–21; Coolen MJ, Orsi WD. The transcriptional response of microbial communities in thawing Alaskan permafrost soils. Frontiers in Microbiology. 2015 Mar 16;6:197). Likewise, thawing itself or the presence of labile organic matter can trigger microbial transcription independent of the original in situ activity (Orsi WD, 2015). This process could contribute to localized ARG transcript peaks, particularly at depths with higher nutrient availability, redox variation, or preserved organic matter.

Response: *We thank the reviewer for this insightful comment. We agree that the detected ARG transcript patterns may not exclusively reflect active in situ microbial expression. In the revised manuscript, we have added clarification that permafrost systems may contain residual nucleic acids derived from microbial necromass, which can persist under frozen conditions and potentially influence transcript detection. We also acknowledge that thaw-related processes or exposure to labile organic substrates may induce transcriptional responses in surviving microbial populations independent of their original in situ activity. These considerations have been incorporated into the Discussion section to provide a more cautious interpretation of depth-dependent ARG expression patterns. We have included the suggested references to support these processes. These revisions improve the robustness and balance of our interpretation without altering the observed results. Page # 9: Lines 298-304*.

Lines 124-133: The reported depth-dependent variation in resistance mechanisms raises an ecological question that warrants further clarification. In dynamic surface soils such as the active layer, recurrent freeze–thaw cycles, periodic inputs of fresh organic matter, and higher microbial turnover are expected to intensify microbial competition and selective pressures. Experimental and ecological studies have shown that competitive microbial growth strategies in soils are associated with increased ARG abundance and diversification (Liu Z, Yao X, Chen C, Zhao Y, Dong C, Sun L, Zhao J, Zhang B, Yu Z, Cheng D, Zhu L. Growth of microbes in competitive lifestyles promotes increased ARGs in soil microbiota: insights based on genetic traits. Microbiome. 2025 Jan 13;13(1):8). In contrast, deep permafrost communities are typically structured by long-term environmental stability, low energy flux, and strong abiotic filtering, where functional gene distributions may reflect legacy signals and environmental constraints rather than active competitive dynamics (Waldrop MP, Chabot CL, Liebner S, Holm S, Snyder MW, Dillon M, Dudgeon SR, Douglas TA, Leewis MC, Walter Anthony KM, McFarland JW. Permafrost microbial communities and functional genes are structured by latitudinal and soil geochemical gradients. The ISME Journal. 2023 Aug;17(8):1224-35). Additionally, studies of ecological succession in glacial forefields demonstrate that ARG prevalence shifts with environmental selection and community turnover (Gou Y, Liu Y, Hu A, Mao G, Dong R, Li S, Liu P, Liu Y, Ji M. Dissemination of genes associated with antibiotic resistance and bacterial virulence during ecosystem succession in two Tibetan glacier forefields. Science of The Total Environment. 2025 Feb 1;963:178514). Given this ecological framework, it would be helpful for the authors to clarify whether the higher diversity or mechanistic breadth of ARGs observed in specific layers aligns with expected competitive dynamics in the active layer versus the comparatively stable, energy-limited permafrost strata. If deeper layers show equal or greater mechanistic diversity, additional explanation is needed to distinguish between active in situ expression, legacy functional potential, or methodological artifacts.

Response: *We thank the reviewer for this insightful comment. We agree that the observed depth-dependent variation in antibiotic resistance genes requires careful ecological interpretation. In the revised manuscript, we have added a brief clarification in the discussion section indicating that differences in ARG composition across soil horizons likely reflect contrasting ecological*

conditions, including higher microbial turnover and environmental variability in the active layer, versus long-term stability and stronger abiotic filtering in deeper permafrost layers. Page# 8: Lines 241-256.

In addition, we have expanded the Discussion to provide a more detailed ecological framework. Specifically, we highlight that competitive microbial dynamics may be more pronounced in surface soils, whereas functional gene patterns in deeper layers may be shaped primarily by environmental filtering, long-term preservation, and legacy effects. We further clarify that the mechanistic diversity observed in deeper permafrost likely reflects a combination of these factors rather than exclusively active ecological processes. These revisions improve the ecological interpretation of the results and ensure a more balanced and cautious framing of the observed patterns, without altering the original findings.

Please ensure that all gene names are consistently italicized throughout the manuscript in accordance with standard nomenclature conventions. See https://insight.jci.org/kiosk/publish/genestyle?utm_source=chatgpt.com

Response: *We thank the reviewer for pointing this out. We have carefully revised the manuscript to ensure that all gene names are consistently italicized in accordance with standard nomenclature conventions (see gene nomenclature guidelines). These changes have been applied throughout the main text.*

Lines 146-148: It would be helpful if the authors could report the measured concentrations of mercury (Hg) and copper (Cu) in the analyzed permafrost samples and directly compare them with those in the corresponding active-layer samples. Providing these quantitative data would allow readers to better assess potential metal-driven co-selection of ARGs. While elevated metal concentrations can contribute to resistance selection, it should be noted that high mercury levels are not universally characteristic of permafrost environments (e.g. see Zhao Y, Hu HW, Su JQ, Hao X, Guo H, Liu YR, Zhu YG. Influence of legacy mercury on antibiotic resistomes: evidence from agricultural soils with different cropping systems. Environmental science & technology. 2021 Oct 6;55(20):13913-22.; Antcibor I, Eschenbach A, Zubrzycki S, Kutzbach L, Bolshiyanov D, Pfeiffer EM. Trace metal distribution in pristine permafrost-affected soils of the Lena River delta and its hinterland, northern Siberia, Russia. Biogeosciences. 2014 Jan 2;11(1):1-5.; Perryman CR, Wirsing J, Bennett KA, Brennick O, Perry AL, Williamson N, Ernakovich JG. Heavy metals in the Arctic: Distribution and enrichment of five metals in Alaskan soils. PLoS One. 2020 Jun 3;15(6):e0233297). Therefore, site-specific measurements are essential to support any interpretation linking ARG patterns to metal exposure.

Response: *We thank the reviewer for this important suggestion. We agree that quantitative metal concentration data are essential for evaluating potential co-selection mechanisms. In our study, trace element concentrations, including Copper, were previously measured and reported in the Supplementary Materials of our earlier publication (Wen et al., 2024, ref. 90). We have now incorporated a comparison of Cu concentrations between permafrost and active-layer samples in the revised manuscript to provide additional context for the observed distribution of metal*

tolerance genes. (Page# 5: Lines 151-154 and Supplementary File S3_Metals Concentrations in Samples).

However, Mercury concentrations were not measured in this study. We have therefore clarified that the potential role of Hg in shaping MTG patterns cannot be directly assessed and that interpretations of metal-driven co-selection remain tentative. Accordingly, we revised the manuscript to emphasize that metal-related interpretations are based on available Cu data only and should be considered with caution. These revisions ensure a more conservative and evidence-based interpretation.

Discussion, line 202: The reference to "pre-anthropogenic strata" may be valid; however, it still remains unclear whether the authors have considered potential stratigraphic reworking associated with past glacial maxima, periglacial processes, or the existence of paleo–active layers.

Response: *We thank the reviewer for this important comment. We agree that the term "pre-anthropogenic strata" may imply a level of stratigraphic and temporal certainty that is not fully supported by the available data. We have therefore revised the text to clarify that these interpretations are based on depth and environmental context rather than direct geochronological constraints, and we now explicitly acknowledge that past glacial, periglacial, and cryogenic processes may have influenced sediment structure and preservation. Page# 7; Lines 220-222.*

Lines 206-210: Lines 206–210: The statement that permafrost contains viable microorganisms is valid; however, it may oversimplify the ecological complexity of permafrost microbiomes. Permafrost soils are known to contain a mixture of culturable cells, viable but non-culturable cells, dormant cells, and dead biomass, and metabolic activity under in situ conditions is generally minimal and strongly constrained by extreme environmental stress. Previous studies have shown that permafrost microbial communities include viable but non-culturable cells and partially degraded "ghost" cells, reflecting a spectrum of survival states rather than sustained metabolic activity (e.g., Gilichinsky et al. 1989; Rivkina et al. 1998; Vishnivetskaya et al. 2000). Metagenomic analyses further suggest that long-term freezing selects for survival strategies such as dormancy, repair mechanisms, and stress responses rather than active proliferation, with functional potential declining with increasing permafrost age and environmental stress (Mackelprang et al. 2017). This is not intended as a criticism, but rather as contextual information that may help refine the discussion and more accurately frame the physiological state and activity potential of permafrost microbial communities.

Response: *We thank the reviewer for this valuable comment and agree that permafrost microbial communities comprise a spectrum of physiological states, including viable, dormant, and viable-but-non-culturable cells, as well as partially degraded biomass. We have revised the manuscript to better reflect this ecological complexity and to better reflect the site-specific nature of the dataset of metabolic activity. Specifically, we now clarify that while transcriptional signals indicate potential functional activity, in situ metabolic rates are likely low and strongly constrained by environmental conditions. We have incorporated the suggested references (Gilichinsky et al., 1989; Rivkina et al., 1998; Vishnivetskaya et al., 2000; Mackelprang et al., 2017) to provide both*

classical and metagenomic context. These revisions ensure a more accurate and nuanced interpretation of microbial activity in permafrost systems. Page# 7: lines 224-227.

Lines 215-217: While the general statement may be valid, it is difficult to justify without clear age constraints for the analyzed permafrost samples. The phrase "over geological timescales" is particularly broad and may overstate the temporal framework unless supported by explicit dating data.

Response: *We thank the reviewer for this important comment. We agree that the phrase "over geological timescales" may overstate the temporal interpretation in the absence of direct age constraints. We have therefore revised the text to remove this phrasing and instead frame our findings in terms of depth-resolved and environmental gradients. This change ensures that our conclusions remain consistent with the acknowledged limitations regarding the lack of geochronological data. Page# 7: line 220.*

Lines 225-227: The authors propose horizontal gene transfer (HGT) as a driver of ARG distribution; however, it is not clear how this process could occur in a system where microbial movement is extremely limited and cells are effectively trapped within a spatially constrained, frozen microenvironment. Permafrost communities are strongly shaped by dispersal limitation and physical constraints, which reduce cell–cell contact and limit opportunities for the primary mechanisms of HGT (conjugation, transduction, transformation) that typically require mobility or interaction among organisms (e.g., Bottos EM, Kennedy DW, Romero EB, Fansler SJ, Brown JM, Bramer LM, Chu RK, Tfaily MM, Jansson JK, Stegen JC. Dispersal limitation and thermodynamic constraints govern spatial structure of permafrost microbial communities. FEMS microbiology ecology. 2018 Aug;94(8):fiy110.). While evidence of HGT exists in deep subsurface environments under slow growth conditions (e.g., Labonté JM, Field EK, Lau M, Chivian D, Van Heerden E, Wommack KE, Kieft TL, Onstott TC, Stepanauskas R. Single cell genomics indicates horizontal gene transfer and viral infections in a deep subsurface Firmicutes population. Frontiers in microbiology. 2015 Apr 22;6:349.), these environments still maintain some degree of microbial activity and interaction that may not be comparable to permanently frozen permafrost. Given the strong physical and ecological constraints on microbial mobility in frozen soils, the authors should clarify the ecological plausibility of frequent HGT and discuss whether alternative explanations (e.g., ancient HGT events preserved in genomes, relic DNA inheritance, or slow selective retention over evolutionary timescales) might better account for the observed ARG patterns.

Response: *We thank the reviewer for this insightful comment regarding the ecological plausibility of horizontal gene transfer (HGT) in frozen permafrost systems. We agree that strong physical constraints in permafrost, including dispersal limitation, reduced microbial mobility, frozen microstructures, and limited cell–cell contact, likely restrict opportunities for active HGT processes such as conjugation, transformation, and transduction (Bottos et al., 2018).*

In response, we revised the manuscript to clarify that our data do not provide direct evidence of ongoing HGT in deep permafrost. We now explicitly state that the observed associations among

ARGs, MGEs, and microbial taxa are consistent with the potential for mobilization but do not demonstrate active gene transfer under frozen conditions (Page 8, lines 265–273).

We also incorporated alternative ecological interpretations proposed by the reviewer, including the possibility that the observed ARG patterns reflect historical HGT events preserved within genomes, vertical inheritance, long-term ecological filtering, or selective retention over extended evolutionary timescales. In addition, although HGT has been documented in deep subsurface microbial systems under low-energy conditions (Labonté et al., 2015), we now clarify that these environments may not be directly comparable to permanently frozen permafrost, where physical isolation and constrained microbial interactions are likely more pronounced. These revisions provide a more cautious and ecologically grounded interpretation of ARG persistence and mobilization potential in deep permafrost systems, and the suggested references have been incorporated to better contextualize these limitations.

Lines 256-258: Did the authors consider using models specifically designed for sparse count data, such as BAMZINB (Hajihosseini M, Amini P, Saidi-Mehrabad A, Dinu I. Infants' gut microbiome data: A Bayesian Marginal Zero-inflated Negative Binomial regression model for multivariate analyses of count data. Computational and Structural Biotechnology Journal. 2023;21:1621–1629), to test for relationships between environmental variables and microbial taxa? BAMZINB is particularly well suited for highly sparse microbiome datasets, as it accounts for zero inflation and overdispersion, and could provide a more robust assessment of which taxa are associated with ARG abundances or environmental gradients in these permafrost samples.

Response:

We thank the reviewer for this valuable suggestion. We agree that models specifically designed for sparse and zero-inflated microbiome data, such as BAMZINB, provide a powerful framework for analyzing overdispersed count datasets and may improve inference for taxa–environment relationships in complex microbial communities.

In the present study, however, analyses were performed primarily on TPM-normalized transcript abundance profiles rather than raw count data, and our objective was to identify broad ecological and transcriptional association patterns using exploratory multivariate approaches (e.g., correlation analyses, ordination, Mantel tests, and SEM). In addition, the relatively small sample size, depth-structured pseudo-replicate design, and limited number of independent cores constrain the statistical power and parameter stability required for more complex Bayesian zero-inflated models.

Nevertheless, we agree that Bayesian or zero-inflated negative binomial frameworks represent an important direction for future studies, particularly for larger multi-core datasets with greater replication and raw count-based modeling. Accordingly, we have added a statement in the revised Discussion acknowledging this methodological consideration and highlighting sparse-data statistical frameworks (including Bayesian and zero-inflated approaches) as valuable future analytical tools for resolving ARG–taxa–environment relationships in permafrost microbiomes. Page# 13: Lines 456-461.

Lines 265-267: Does this statement align with the context of the Stress Gradient Hypothesis (Hernandez DJ, David AS, Menges ES, Searcy CA, Afkhami ME. Environmental stress destabilizes microbial networks. The ISME Journal. 2021;15(6):1722–1734)?

Response:

We thank the reviewer for this insightful comment. We agree that our observations of increased genome plasticity under cryogenic stress may conceptually align with ecological frameworks such as the Stress Gradient Hypothesis, which proposes that environmental stress can influence microbial interactions and network structure. However, as our data are based on correlative transcriptomic patterns rather than direct measurements of microbial interactions or network stability, we have revised the text to avoid overinterpretation and to clarify that these observations are consistent with, but do not directly test, such theoretical frameworks. Page# 10; Lines 241-344.

Line 272: The authors should refrain from using outdated nomenclature. Please update Firmicutes to Bacillota and Euryarchaeota to Methanobacteriota throughout the manuscript. For accuracy, the authors may consult the DSMZ LPSN database to verify current taxonomic names and ensure consistent usage.

Response: We thank the reviewer for this helpful suggestion. We have updated taxonomic nomenclature throughout the manuscript in accordance with current standards (e.g., LPSN), including replacing Firmicutes with Bacillota and Euryarchaeota with Methanobacteriota. The original names are retained at first mention for clarity, and consistent usage has been ensured across the main text, figures, and supplementary materials. Page# 10; Lines 350-351; 353; 355; 368.

Line 275: change the Proteobacteria to Pseudomonadota.

Response: *We thank the reviewer for this suggestion. We have updated taxonomic nomenclature throughout the manuscript to reflect current standards, including replacing Proteobacteria with Pseudomonadota (formerly Proteobacteria), and ensuring consistent usage across the main text and supplementary materials. Page# 10; Line 355.*

Reviewer #2 (Remarks to the Author):

The study system is interesting and logistically challenging, however there are some key methodological and analytical limitations requiring substantial revision of the statistical approach.

Response: We thank the reviewer for recognizing the novelty and logistical difficulty of working with deep permafrost cores in this study system. We also appreciate the constructive comments regarding the statistical and analytical framework. We acknowledge that several aspects of the statistical approach required clarification and refinement. In the revised manuscript, we have substantially improved the description of statistical methods, including clearer justification of analytical choices, improved treatment of depth-resolved structure, and additional clarification of replicate structure and limitations associated with core-level independence. We believe these revisions strengthen the robustness and interpretability of the results.

1) The number of (pseudo)replicates per core and per layer type is not clear. This is important in determining how many samples (group sizes) were used for comparison of permafrost horizons to evaluate the robustness and balance of the statistical tests.

Response: *We thank the reviewer for this important comment regarding the number and structure of replicates used in the analysis. In the revised manuscript, we have clarified the sampling design in the Methods section (Page# 13; Line 471). A total of 25 stratified samples were collected, including 10 from TTH-04 and 15 from TTH-07, distributed across three depth-defined horizons (active layer, permafrost, and sub-permafrost), as detailed in Table S4. Due to logistical constraints associated with deep drilling and core recovery, the number of samples within each horizon is not uniform. We further clarify that samples collected across depth intervals within each core represent pseudo-replicates, as they are not independent biological replicates but rather depth-resolved subsamples from the same core. Accordingly, statistical analyses were designed to evaluate within-core, depth-related patterns, rather than to perform fully balanced comparisons across horizons or between sites. These clarifications have now been incorporated into the revised Methods section to improve transparency and to ensure appropriate interpretation of the statistical analyses. Page# 13; Lines 471-481.*

2) From Supplementary: "Within each core, multiple stratified subsamples from the active layer, permafrost, and sub-permafrost horizons were treated as replicate units to evaluate within-core, depth-resolved patterns."

I understand that this study is logistically difficult in terms of gathering the samples. Nevertheless, grouping across depth layers into permafrost horizons could have been avoided (though I understand the initial desire to do this to simplify interpretation). The current subsamples within each core are treated as independent replicates, despite being along a continuous gradient. Using depth as a continuous variable would have been more statistically robust for analysis of ARG abundances/diversity trends/networks than collapsing samples into three permafrost horizons (active, permafrost, sub-permafrost). Or at least controlling for depth. Grouping samples into discrete permafrost horizons collapses a continuous gradient into categorical comparisons. Though this would not fully fix the pseudo-replication issue of subsampling within the same cores, it would strengthen the analysis interpretations. Even if this is a site-specific study, the statistical treatment of the data is still not fully appropriate for the type of data, and this affects the reliability of the conclusions.

Response: We thank the reviewer for this thoughtful and constructive comment regarding the statistical treatment of depth and sample grouping. We agree that depth in permafrost systems represents a continuous environmental gradient, and that grouping samples into discrete horizons may reduce resolution and potentially obscure fine-scale variation. However, in this study, samples were categorized into active layer, permafrost, and sub-permafrost horizons based on established cryostratigraphic, thermal, and ice-content criteria (Lines# 495-499 in the main manuscript), and followed these studies for classifications 101. Yost, J.L. & Hartemink, A.E. How deep is the soil studied – an analysis of four soil science journals. Plant Soil. 452, 5–18 (2020); 102. Zhang,

T. Geocryology in China. Permafr. Periglac. Process. 12, 315–322 (2001).), which define ecologically and physically distinct regimes rather than arbitrary groupings (details are shown in Table S4). These stratified horizons are commonly used in permafrost research because they represent ecologically and physically distinct regimes characterized by differences in thermal stability, hydrology, substrate availability, and microbial habitat structure. While we agree that modeling depth as a continuous variable can provide higher resolution in some contexts, the lack of a significant linear relationship in our dataset (Page# 6; Lines 174-176; and Fig 5a), together with established stratigraphic boundaries, supported the use of a horizon-based framework for ecological interpretation.

Importantly, we also evaluated depth as a continuous variable in our analyses (SEM analysis; Fig 8). As now clarified in the revised manuscript, depth did not show a significant relationship with ARG expression in either core (Page# 6; Lines 174-176; and Fig 5a), suggesting that the observed patterns are not solely driven by linear depth gradients but may instead reflect transitions between functionally distinct stratigraphic horizons.

We acknowledge that subsamples within each core represent pseudo-replicates along a continuous profile and are not fully independent biological replicates. Accordingly, we have revised the Methods and Discussion sections to more explicitly state this limitation and to clarify that our statistical analyses are designed to evaluate within-core, depth-resolved patterns, rather than fully independent or balanced comparisons across horizons or sites. Lines 601-608;

These revisions improve transparency in the statistical framework while maintaining the ecological relevance of stratified permafrost horizons for interpreting transcriptomic patterns.

3) The negative and no-RT controls were not sequenced because there was not sufficient input for the library preparation (Supplementary data). However, for these types of low-biomass samples, it is important to sequence negative controls to identify any potential sources of contamination, even with insufficient/low input for library prep.

Response: We thank the reviewer for this important comment regarding the sequencing of negative and no-RT controls. We agree that sequencing controls is ideal for low-biomass metatranscriptomic studies to directly assess potential contamination sources.

In our study, multiple layers of experimental controls were implemented. Extraction blanks and no-RT (reverse transcription–minus) controls were included throughout the workflow to monitor potential contamination and residual DNA carryover. Importantly, no detectable amplification signal was observed in no-RT controls following reverse transcription and PCR amplification steps, indicating effective removal of genomic DNA and negligible contribution of residual DNA to the cDNA libraries. Line# 157-158 in supplementary materials and Table S5.

However, these controls consistently yielded nucleic acid concentrations below the minimum threshold required for library preparation, and therefore, sequencing libraries could not be constructed despite repeated attempts. This limitation reflects the extremely low biomass nature of deep permafrost samples rather than the omission of experimental controls.

To further mitigate potential contamination concerns, we applied multiple independent safeguards, including (i) sterile field and laboratory workflows under RNase/DNase-free conditions, (ii) strict

cold-chain preservation to prevent post-sampling transcriptional activity, and (iii) stringent downstream bioinformatic filtering, including removal of low-quality reads, and conservative mapping thresholds for functional annotation.

We have clarified these procedures in the revised Supplementary Methods to improve transparency regarding contamination control and explicitly acknowledge residual uncertainty inherent to ultra-low biomass metatranscriptomic systems. Lines 54-60; 63-75; 80-92.

While sequencing of negative controls is ideal in low-biomass studies, this was not technically feasible due to insufficient input material, a common limitation in deep permafrost metatranscriptomics.

4) It does not seem like correction for multiple testing was performed with Pearson correlations, Mantel tests, network assembly (correction for multiple tests only done for: "Differential ARG expression across layers was evaluated in STAMP using Wilcoxon rank-sum tests with Benjamini–Hochberg FDR correction" -line 475)? Lack multiple test correction across all statistical tests in such a dataset (already exposed to pseudo replication) may result in large number of reported false positives and overinterpretation.

Response: We thank the reviewer for highlighting the importance of correcting for multiple testing across the large number of statistical comparisons performed in this study. We agree that without appropriate correction, there is a risk of inflated false positives and overinterpretation.

In response, we have now systematically applied false discovery rate (FDR) correction using the Benjamini–Hochberg procedure across all relevant statistical analyses, including Mantel tests (corrected p values provided in Table S2 and .Source Data files associated with Fig. 6: Source Data for Figure 6 (Manuscript)) and correlation-based network analyses (corrected p values provided in Source Data files associated with Fig. 6: Source Data for Figure 7 (Manuscript)). The p-value correction statements are provided in the respective legends of each figure.

For Mantel and partial Mantel analyses (Fig. 6), we found that only a subset of associations remained significant after FDR correction ($q < 0.05$). Accordingly, we have revised the Results (Page# 6; Lines 193-198) and Discussion sections (Page# 11: Lines 374-378) to clearly distinguish between exploratory associations based on nominal p-values and statistically robust relationships supported by FDR-adjusted significance. These revisions reduce the risk of overinterpretation and emphasize only the most reliable environmental drivers.

For the co-occurrence network analysis (Fig. 7), we note that edges were initially filtered based on strong correlation thresholds ($|r| \geq 0.6$) and nominal significance ($p \leq 0.05$), resulting in a reduced set of high-confidence associations prior to FDR correction. After applying FDR correction across all tested associations ($n = 1255$), the majority of edges in TTH-04 (1121/1255) and all retained edges in TTH-07 remained significant ($q < 0.05$). This indicates that the inferred network structure is robust and not driven by spurious correlations. We have clarified this procedure in the Methods section (Page# 16: Lines 589-594) and explicitly reported FDR-

corrected results in the figure legends (Page# 26: lines 71-74) and Source Data (Source Data for Figure 7 (Manuscript)).

We also acknowledge the reviewer's point regarding potential pseudo-replication. While our study is based on depth-resolved samples from a limited number of permafrost cores, we have taken a conservative approach by applying stringent correlation thresholds, permutation-based Mantel tests, and multiple testing correction. We have additionally tempered our interpretations to reflect the exploratory nature of some associations and now explicitly discuss limitations related to sample size and statistical power. Overall, these revisions ensure that multiple testing is appropriately controlled across analyses and that our conclusions are supported by statistically robust evidence while avoiding overinterpretation.

5) The authors report metatranscriptomic data but there is very limited interpretation of the broader "active" microbial community beyond ARG-centric analyses. As ARG expression is dependent on the microbial taxa expressing these genes, the direct lack of integration between community composition and ARG patterns limits biological interpretation. It remains unclear whether the reported permafrost horizon patterns reflect shifts in active microbial taxa.

Response: We thank the reviewer for this insightful comment regarding the integration between metatranscriptomic ARG profiles and the broader microbial community.

In the revised manuscript, we strengthened the interpretation by explicitly examining relationships between ARG expression patterns, microbial abundance, MGEs, and microbial community composition. ARG expression showed significant positive correlations with microbial abundance and MGEs within both cores, whereas depth alone showed no significant relationship with ARG transcripts or MGEs. Page# 6; Lines 172-176. In addition, Spearman correlation analyses (Fig. S6) identified associations between ARG families and dominant microbial phyla across depth horizons. SEM analyses additionally evaluated microbial community variables as potential contributors to ARG variation, although these effects were weaker than those associated with environmental and mobilome-related factors. Together, these analyses indicate that depth-resolved resistome variation is more closely associated with shifts in transcriptionally active microbial communities and mobilization potential than with depth itself. Accordingly, we have clarified in the Results section that depth-dependent variation in ARG expression is associated with shifts in the transcriptionally active microbial community structure. In the Discussion, we further emphasize that these associations reflect statistical and ecological coupling rather than direct mechanistic inference, and provide a contextual framework for interpreting resistome patterns across permafrost strata.

6) The TPM normalization performed in this study allows the relative comparison of ARGs within the transcript pool, it does not capture absolute transcript abundance or total activity. Therefore, throughout the manuscript, statements claiming increased ARG activity and abundance across depth layers (permafrost horizons) may be an overinterpretation of the current data. These results

could be interpreted as shifts in relative expression instead of absolute differences. This needs to be reworked beyond adding a simple caveat.

Response: *We thank the reviewer for this important comment regarding the interpretation of TPM-normalized metatranscriptomic data. We fully agree that TPM normalization reflects the relative representation of transcripts within the sequenced pool and does not provide a direct measure of absolute transcript abundance or total transcriptional activity.*

In response, we have carefully revised the manuscript to ensure that all interpretations are consistent with this limitation and go beyond a simple caveat. Specifically, we have:

(i) ***expanded the Methods section*** *to explicitly clarify the assumptions and limitations of TPM normalization, emphasizing that it enables comparison of relative transcript representation across samples but does not quantify absolute expression levels. Page# 15: Lines 553-555.*
(ii) ***systematically revised terminology throughout the Abstract, Results, and Discussion****, replacing expressions such as "increased activity" or "higher abundance" with more accurate phrasing (e.g., "higher relative expression," "greater transcript representation," or "relative enrichment of ARG transcripts"). Page# 2: Lines 6-7; line 14: Page# 4: lines 89; 95; 104-105: Page# 6: Lines 171; 181: Page# 7; line 235: Page# 8; Lines 241; 252: Page# 13; Lines 450-451.*
(iii) ***reframed key interpretative statements*** *to ensure that depth-related patterns are consistently described as shifts in relative transcript composition, rather than absolute changes in transcriptional activity; Page# 2: Lines 6-7; line 14: Page# 4: lines 89; 95; 104-105: Page# 6: Lines 171; 181: Page# 7; line 235: Page# 8; Lines 241; 252: Page# 13; Lines 450-451*
(iv) ***added an explicit interpretative statement in the Discussion*** *highlighting that the observed patterns reflect relative differences within the transcript pool and should not be interpreted as direct measures of total microbial activity or gene expression rates. In discussion, page# 8; lines 250-255. In Supplementary Methods "S1.3 Sequencing and meta-transcriptomic analysis lines 219-222.*
Together, these revisions ensure that the interpretation of ARG transcription is fully aligned with the constraints of TPM-based metatranscriptomic data, while preserving the biological significance of the observed depth-resolved patterns.

Reviewer #3 (Remarks to the Author):

Sajjad and colleagues investigated depth-resolved metatranscriptomes from two sampling cores bridging the active layer, permafrost and sub-permafrost, focusing on MTGs/ARGs expressed in the different layers and their potential origins like viruses or Transposons. The manuscript is very well written, with a trough of detail even including additional supplementary methods and reasoned out findings and conclusions. I do not really have any major concerns about this study but have listed a few suggestions below.
Response: We sincerely thank the reviewer for the positive and encouraging assessment of our manuscript. We appreciate the recognition of the novelty and detail of our meta-transcriptomic

approach, as well as the constructive suggestions provided. We have carefully considered all comments and revised the manuscript accordingly to improve clarity, reproducibility, and presentation.

minor comments

Affiliation section – Monitoring / Institution (there are two typos in the affiliations)

Response: We thank the reviewer for noting these typographical errors. The affiliations have been carefully reviewed and corrected in the revised manuscript.

L16 - As the analysis is

Response: We thank the reviewer for noting this issue. The sentence has been revised for clarity in the updated manuscript. Page# 2: Line 17.

L86-88 – Switching between storage space size and read counts makes it more difficult to relate the read counts per sample and the total storage space. I'd consider making the measures uniform (or at least give total read length across all samples instead of storage space size) to make it easier to relate the numbers.

Response: We thank the reviewer for this helpful suggestion. We agree that consistent reporting improves clarity. We have revised the text to present sequencing output using consistent sequencing metrics based on read counts and total sequence yield (in base pairs), and have removed the use of storage size (GB) to improve interpretability. Page# 4: Lines 87-89.

L394- 396 – a link or reference to these established protocols would be good to add as without them, the analyses are not really reproducible.

Response: We thank the reviewer for this suggestion. We have clarified the soil pH measurement method by specifying the instrument manufacturer and referencing the updated standard protocol (ISO 10390:2021; Page#14; Line 509 and Reference# 103) to improve reproducibility. In addition, we have revised the Methods section (Lines 519–522) to include a reference to established microwave-assisted digestion protocols for environmental solids (EPA Method 3052; reference# 104) and clarified the use of the MARS Xpress system (CEM, USA), thereby improving methodological transparency and reproducibility.

L406 – add product/article number

Response: *We thank the reviewer for this suggestion. We have revised the Methods section to include catalog numbers for key reagents to improve methodological clarity and reproducibility. Specifically, we have added the catalog numbers for TRIzol™ Reagent (Invitrogen, Cat. No. 15596026) used for RNA extraction and the HiScript® Reverse Transcriptase kit (Vazyme Biotech, Cat. No. R101-01) used for cDNA synthesis. Page# 14; Lines 532 and 534.*

L407 – im curious as to the reasoning behind focusing on nonpolyadenylated transcripts – is that just a technical reason (i.e., the RTase you use excludes them) or is there a scientific reason?

Response: We thank the reviewer for this insightful comment. The use of random hexamer primers was a deliberate methodological and scientific choice to enable comprehensive and unbiased capture of microbial transcripts. Unlike eukaryotic mRNA, most bacterial and archaeal transcripts lack poly(A) tails and would therefore not be efficiently captured using oligo(dT)-based approaches. We have clarified this rationale in the Supplementary Methods (Section S1.3 Sequencing and meta-transcriptomic analysis; Lines 173-176) to improve transparency and ensure accurate interpretation of the metatranscriptomic approach.

L409 – add NovaSeq instrument identifier (X / 6000 / …); Also specify that you did not do any type of rRNA depletion or similar (that's my assumption as it isnt mentioned) and the library prep kit.

Response: We thank the reviewer for this helpful suggestion. We have revised the Methods section to specify the sequencing platform (Illumina NovaSeq 6000) (Page 15: Lines 538-539) and clarified that no rRNA depletion or poly(A) enrichment was performed due to the low RNA yield and the need to retain non-polyadenylated microbial transcripts (Page# 15: Lines 542-544; also, already mentioned in supplementary methods S1.3 Sequencing and meta-transcriptomic analysis; Page# 6: Lines 199-201). As library preparation was conducted by the sequencing provider, we have indicated that library preparation was performed by the sequencing provider using standard low-input RNA workflows. Page# 15; Lines 538-540. Also, provided references of our previous study "38. Wen, Q. et al. Viral communities locked in high elevation permafrost up to 100 m in depth on the Tibetan Plateau. Sci Total Environ. 932, 172829 (2024).

L417 – maybe a bit more specification than "~30-40" is possible? E.g., via mean and SD % per AMG database categories covered by your data or so? Or give a single number with "~" as it already implies a range around the given value.

Response: *We thank the reviewer for this helpful suggestion. We have replaced the approximate range ("~30–40") with a more precise value based on the SARG database (v2.0), specifying that ~38 ARG classes are defined. This improves clarity and consistency in the description of ARG classification. We did not include percentage-based coverage statistics, as this statement refers to the total number of ARG classes defined in the reference database rather than sample-level variation. Page# 15: Lines 557-558.*

L442 – please ensure that all statistical tests done within the tutools platform are described in sufficient detail to allow reproducing in more open-source platforms like R. To this end, please specify numbers of permutations, p-value adjustment, alpha error values used and so on (as I do not have an account on the tutools platform, I cannot really see what the defaults are and whether they are similar to alternatives in, e.g., R).

Response: *We thank the reviewer for this important comment. We agree that analyses conducted via platform-based tools should be described in sufficient detail to ensure reproducibility. We have now expanded the Methods section to explicitly report key statistical parameters, including the distance metric (Bray–Curtis), number of permutations for Mantel tests (9,999), use of two-tailed tests for Pearson correlations, application of Benjamini–Hochberg FDR correction for multiple*

comparisons, and the significance threshold (α = 0.05). These additions improve transparency and facilitate reproducibility in open-source environments such as R. Page# 16: Lines 580-587.

more a comment: To me it looks a lot nicer if the format of the affiliations/title/authors in the main and the SI are identical. I'd also make font+sizes uniform in the SI text passages.
Response: *We thank the reviewer for this helpful suggestion. We have revised the Supplementary Information to ensure that the formatting of the title, author list, and affiliations is consistent with the main manuscript. In addition, font styles and sizes have been standardized throughout the SI to improve clarity and presentation.*

Fig 3a – convert y axis fractions to percentages to match axis title
Response: We thank the reviewer for this helpful suggestion. The y-axis in Fig 1a, 1c, and Fig. 3a has been revised to display values as percentages, and all corresponding labels have been updated to ensure consistency and clarity.

Supplemental data file names – The file names I got when downloading the zipped files are all indistinguishable (just a sequential number counting up). That makes it rather time-consuming to cross-reference your in-text references to, e.g., Table S3, as there is no file (and I also don't see a sheet in any Excel file) with that name.
Response: *We thank the reviewer for this helpful comment. We have revised the naming and organization of all source data files to ensure clear correspondence with the figures cited in the manuscript and Supplementary Information (e.g., "Source Data for Fig1… (manuscript)", "Source Data for Fig S2… (Supplementary Information)"), and sheet names within Excel files have been labeled accordingly. We clarify that Supplementary Tables (e.g., Table S3) are provided within the Supplementary Information Word document rather than as separate Excel files. This has now been clearly indicated to improve cross-referencing and ease of navigation.*

Reviewer #4 (Remarks to the Author):

The study addresses a clear core scientific question with prominent innovations, standardised technical methods, and sufficient data support. There are no scientific flaws or methodological errors. The main issues focus on the explanation of sample representativeness and optimization of result presentation. These can be improved through revisions.
Response: *We thank the reviewer for the positive evaluation of our study and for recognizing its novelty and methodological rigor. We appreciate the constructive suggestions provided to improve the clarity of sample representativeness and the presentation of results. We have carefully revised the manuscript to address these points and highlighted them in yellow in the revised manuscript, as detailed below.*

1. Insufficient evaluation of sample size limitations and inadequate constraint on conclusion expression (Abstract: Lines 36-37; Introduction: Lines 82-83; Results: Lines 98-100; Discussion: Lines 345-347). Only two permafrost cores (TTH-04: 40 m; TTH-07: 100 m) were selected in the

study without spatial replicate samples. Although the authors repeatedly mentioned that the results were site-specific, the constraints imposed by this limitation on the study's conclusions were not consistently emphasized when presenting key findings (e.g., "antibiotic inactivation is the dominant resistance mechanism in permafrost" and "deep permafrost acts as a reservoir of genetic novelty").

Response: *We thank the reviewer for this important comment. We agree that, although the site-specific nature of the study was noted, the limitations associated with the lack of spatial replication were not consistently emphasized when presenting key findings. We have revised the Abstract, Introduction, Results, and Discussion to moderate conclusion strength and explicitly frame our findings as site-specific, depth-resolved observations. Statements implying generalizable patterns (e.g., regarding dominant resistance mechanisms and the role of deep permafrost as a reservoir) have been carefully reworded to avoid overinterpretation and to highlight the need for broader validation across multiple sites.*

We have specifically revised statements such as "deep permafrost acts as a reservoir of genetic novelty" to more cautious phrasing (e.g., "may serve as a reservoir… in these cores") to better reflect the site-specific nature of the dataset. Page# 8: Lines 261-261.

We have revised statements in the Abstract to explicitly constrain interpretations to the analyzed cores and to better reflect the site-specific nature of the dataset. Page# 2: Lines 10-11.

We have revised statements in the Discussion to constrain interpretations to the analyzed cores and avoid overgeneralization, particularly when comparing with other permafrost environments. Page# 9: Lines 290; 306, and Page# 8: Lines 261 and 256.

2.The abbreviation "MTGs" first appeared in Line 28 of the abstract without definition, and it was not explained as "metal tolerance genes" until Line 146 of the results section.

Response: *We thank the reviewer for pointing this out. We have now defined the abbreviation "MTGs" (metal tolerance genes) at its first occurrence in the Abstract to improve clarity and consistency. Page# 2: Line 15.*

3. In the transcriptome analysis, the selection basis of k-mer (21–141) for MEGAHIT assembly was not stated, and whether gene length was considered in the TPM normalization of Salmon quantification was not explained.

Response: *We thank the reviewer for this helpful comment. We have clarified that de novo assembly was performed using MEGAHIT with a multi–k-mer strategy (k = 21–141) and a minimum contig length of 500 bp, commonly used to improve recovery of both low-abundance and longer transcripts. We have also explicitly stated that TPM normalization using Salmon accounts for both gene length and sequencing depth, ensuring appropriate normalization of transcript abundances across samples. Page# 15: Lines 544-548 in main file; Page# 6: Lines 219-220; Supplementary methods S1.3 Sequencing and meta-transcriptomic analysis in supplementary materials.*

4.The number of ARG subtypes marked in the UpSet plot of Figure 3c was inconsistent with the calculation results of "40% (TTH-04) and 34.6% (TTH-07) of ARGs were shared" in Lines 165-167 of the text, and the coordinate axis was unlabeled with unclear numerical meaning. ***Response:*** *We thank the reviewer for this observation. We have verified that the reported percentages are correct (TTH-04: 122/305 = 40.0%; TTH-07: 145/419 = 34.6%). To improve clarity and consistency with Fig. 3c, we have revised the text to include both absolute counts and percentages, and we have updated the UpSet plot to include clear axis labels and annotations. Page# 6: Lines 162-165. Also, revised figure 3c, percentages are provided to each bar to indicate shared ARG proportions.*

5.Adonis P=0.28 (no statistical significance) in the PCoA analysis of TTH-04 in Figure 4a, but the text still stated that "β-diversity analysis confirmed depth-specific ARG assemblages" in Lines 162-164, which was contradictory to the figure results.
Response: *We thank the reviewer for highlighting this point. We apologize for the ambiguity and now clarify that the reported P = 0.28 corresponds to PERMDISP (dispersion test), not PERMANOVA. The PERMANOVA (Adonis) analysis showed significant differences among depth-defined groups (P = 0.006), supported by ANOSIM (R = 0.44, P = 0.037). We have revised the text to clearly distinguish these analyses and avoid ambiguity. Page# 6; Lines 165-169.*

6.There was no correlation between depth and ARG abundance in TTH-07 in Figure 5a (R=-0.075, P=0.79), but this result was not mentioned in Lines 170-172 of the text, and only the correlation in TTH-04 was emphasized, resulting in selective data reporting.
Response: *We thank the reviewer for this important comment. We agree that the previous version did not fully report the non-significant relationships between depth and ARG expression. We have revised the manuscript to explicitly include results for both cores (TTH-04: R = −0.48, P = 0.17; TTH-07: R = −0.075, P = 0.79) and now clearly state that depth was not significantly correlated with ARG expression. This ensures balanced and transparent reporting. Page# 6; Lines 174-175. The figures are labeled with TTH-04 and TTH-07 to clarify.*

REVIEWERS' COMMENTS:

Reviewer #2 (Remarks to the Author):

The authors have addressed my comments. I would like to suggest that in future studies they do sequence negative controls despite insufficient material. It is still possible to make libraries with 'undetected' nucleic acids in negative controls and sequence them (you treat it as if you have enough material).

Response: We thank the reviewer for their positive evaluation and constructive suggestion. We agree that sequencing of negative controls, even when nucleic acid concentrations are below detection limits, represents a valuable additional strategy for assessing potential background contamination in low-biomass environmental transcriptomic studies. While such sequencing was not feasible within the scope of the present study, we acknowledge this as an important consideration for future deep permafrost metatranscriptomic investigations. We appreciate the reviewer's recommendation and will incorporate this approach into future experimental designs.

Reviewer #4 (Remarks to the Author):

The authors have adequately addressed all of my previous comments. I have no further concerns and support the publication of this manuscript.

Response: We sincerely thank the reviewer for their positive evaluation of our revised manuscript and for their constructive comments throughout the review process. We are grateful that the revisions have adequately addressed all concerns and appreciate the reviewer's support for the publication of our work.
